# Supplementary material for: Mucosal sugars delineate pyrazine vs pyrazinone autoinducer signaling in Klebsiella oxytoca
Source: Nat Commun. 2024 Oct 16;15:8902. doi: 10.1038/s41467-024-53185-6 (PMC11480411; doi:10.1038/s41467-024-53185-6)

Supplementary Information

Mucosal sugars delineate pyrazine vs pyrazinone autoinducer signaling in *Klebsiella oxytoca*.

Randy Hamchand,[1,2] Kevin Wang,[2] Deguang Song,[3] Noah W. Palm,[3] Jason M. Crawford[1,2,4,*]

[1] Department of Chemistry, Yale University, New Haven, Connecticut, USA.

[2] Institute of Biomolecular Design & Discovery, Yale University, West Haven, Connecticut, USA.

[3] Department of Immunobiology, Yale University School of Medicine, New Haven, Connecticut, USA.

[4] Department of Microbial Pathogenesis, Yale University School of Medicine, New Haven, Connecticut, USA.

[*] Correspondence may be addressed to Jason M. Crawford, jason.crawford@yale.edu

Table of Contents

Table S1. DNA constructs used in this study. 6

Table S2. ^1H and ^{13}C NMR assignments of natural 6 (600 MHz, methanol-d_4). 7

Table S3. ^1H and ^{13}C NMR assignments of natural 7 (600 MHz, acetonitrile-d_3). 8

Table S4. ^1H and ^{13}C NMR assignments of isolated 9-Me (600 MHz, acetonitrile-d_3). Me substituent arose under methanolic isolation conditions of natural metabolite 9. 9

Table S5. ^1H and ^{13}C NMR assignments of synthetic 18 (400 MHz, methanol-d_4). 10

Table S6. ^1H and ^{13}C NMR assignments of synthetic 19 (400 MHz, methanol-d_4). 11

Table S7. ^1H and ^{13}C NMR assignments of synthetic 20 (400 MHz, methanol-d_4). 12

Figure S1. a. Proposed structures of compounds from the leupeptin molecular network. MS^2 analysis of proposed non-adduct features is presented in the Figure S9. b. Architecture of the nan operon from K. oxytoca. 13

Figure S2. a. Operon architecture of the yersiniabactin biosynthetic gene cluster. b. Relative production of pyrazines 5-9 and yersiniabactins 10 and 11 in wildtype K. oxytoca when supplemented with various sugar. 14

Figure S3. a. Biosynthesis of 12 via the PLP-dependent enzyme PqrA en route to the production of perquinolines.[53] b. HPLC-QTOF-MS extracted ion chromatograms of pyrazines 5-9 from wildtype K. oxytoca, Δpyr K. oxytoca, and medium control samples. Compounds 5-9 were not detected in Δpyr K. oxytoca or in growth medium controls. c. HPLC-QTOF-MS extracted ion chromatograms of yersiniabactin 10 from wildtype K. oxytoca, Δpyr K. oxytoca, and medium control samples. 15

Figure S4. Stereochemical studies of the K. oxytoca Pyr protein. 16

Figure S5. ESI^+-HRMS m/z envelope of 1. 17

Figure S6. ESI^+-HRMS/MS fragmentation spectrum of 1 (20 CE). 17

Figure S7. ESI^+-HRMS m/z envelope of 2. 17

Figure S8. ESI^+-HRMS/MS fragmentation spectrum of 2 (20 CE). 17

Figure S9. ESI^+-HRMS m/z envelope of 3. 18

Figure S10. ESI^+-HRMS/MS fragmentation spectrum of natural 3 (20 CE). 18

Figure S11. ESI^+-HRMS/MS fragmentation spectrum of synthetic 3 (20 CE). 18

Figure S12. ESI^+-HRMS m/z envelope of 4. 18

Figure S13. Proposed structures of features from the molecular network in Fig. 1c. 19

Figure S14. ESI^+-HRMS/MS fragmentation spectrum of m/z 395.2753 (N-Acetyl Leu-Val-Arginal – H_2O) from Fig. 1c (20 CE). 19

Figure S15. ESI^+-HRMS/MS fragmentation spectrum of m/z 431.2964 (N-Acetyl Leu-Val-Arginal + H_2O) from Fig. 1c (20 CE). 20

Figure S16. ESI^+-HRMS/MS fragmentation spectrum of m/z 413.2861 (N-Acetyl Leu-Val-Arginal) from Fig. 1c (20 CE). 20

Figure S17. ESI^+-HRMS/MS fragmentation spectrum of m/z 445.3137 (1 + H_2O) from Fig. 1c (20 CE). 20

Figure S18. ESI^+-HRMS/MS fragmentation spectrum of m/z 385.2814 (N-Acetyl Leu-Val-Lysinal) from Fig. 1c (20 CE). 20

Figure S19. ESI^+-HRMS/MS fragmentation spectrum of m/z 459.3269 (1 + MeOH) from Fig. 1c (20 CE). 21

Figure S20. ESI^+-HRMS/MS fragmentation spectrum of m/z 403.2922 (N-Acetyl Leu-Val-Lysinal + H_2O) from Fig. 1c (20 CE). 21

Figure S21. ESI^+-HRMS/MS fragmentation spectrum of m/z 431.3241(2 + MeOH) from Fig. 1c (20 CE). 21

Figure S22. ESI^+-HRMS/MS fragmentation spectrum of m/z 417.3094 (2 + H_2O) from Fig. 1c (20 CE). 21

Figure S23. ESI^+-HRMS/MS fragmentation spectrum of m/z 367.2690 (Daughter Ion of 1) from Fig. 1c (20 CE). 22

Figure S24. ESI^+-HRMS/MS fragmentation spectrum of m/z 381.2850 (2 – H_2O) from Fig. 1c (20 CE). 22

Figure S25. ESI^+-HRMS/MS fragmentation spectrum of m/z 443.2986 (N-Acetyl Leu-Leu-Arg) from Fig. 1c (20 CE). 22

Figure S26. ESI^+-HRMS/MS fragmentation spectrum of m/z 429.2828 (N-Acetyl Leu-Val-Arg) from Fig. 1c (20 CE). 22

Figure S27. ESI^+-HRMS/MS fragmentation spectrum of m/z 401.2765 (N-Acetyl Leu-Val-Lys) from Fig. 1c (20 CE). 23

Figure S28. ESI^+-HRMS/MS fragmentation spectrum of m/z 415.2913 (N-Acetyl Leu-Leu-Lys) from Fig. 1c (20 CE). 23

Figure S29. ESI^+-HRMS/MS fragmentation spectrum of m/z 287.1946 (N-Acetyl Leu-Leu) from Fig. 1c (20 CE). 23

Figure S30. ESI^+-HRMS/MS fragmentation spectrum of m/z 273.1788 (N-Acetyl Leu-Val) from Fig. 1c (20 CE). 23

Figure S31. ESI^+-HRMS analysis showing Neu5Ac (top), but not Neu5Gc (bottom), can induce leupeptin 1 production. 24

Figure S32. Mucin-mediated production of leupeptins 1 and 2 measured via ESI^+-HRMS. 24

Figure S33. The nanA mutant cannot produce leupeptin 1 regardless of mucin or Neu5Ac supplementation. The symbol n.d. stands for "not detected." 26

Figure S34. The nanA mutant cannot produce pyrazinone 3 regardless of Neu5Ac supplementation. Additional peaks likely result from isomeric (pro)pyrazinones which are produced as intermediates (scheme shown). ... 27

Figure S35. ESI^+-HRMS analysis of compounds 10a, 10b, 10-Fe, and 11 between wildtype and Δyer K. oxytoca strains. 28

Figure S36. UV-Vis spectra of compounds 5, 6, 7, 8, and 9. 30

Figure S37. ESI^+-HRMS m/z envelope of 5. 31

Figure S38. ESI^+-HRMS/MS fragmentation spectrum of 5 (20 CE). 31

Figure S39. ESI^+-HRMS m/z envelope of 6. 31

Figure S40. ESI^+-HRMS/MS fragmentation spectrum of 6 (20 CE). 31

Figure S41. ESI^+-HRMS m/z envelope of 7. 32

Figure S42. ESI^+-HRMS/MS fragmentation spectrum of 7 (20 CE). 32

Figure S43. ESI$^+$-HRMS m/z envelope of 8. 32
Figure S44. ESI$^+$-HRMS/MS fragmentation spectrum of 8 (20 CE). 32
Figure S45. ESI$^+$-HRMS m/z envelope of 9. 33
Figure S46. ESI$^+$-HRMS/MS fragmentation spectrum of 9 (20 CE). 33
Figure S47. ESI$^+$-HRMS m/z envelope of 10. 33
Figure S48. ESI$^+$-HRMS m/z envelope of 11. 33
Figure S49. ESI$^+$-HRMS m/z envelope of 12. 34
Figure S50. ESI$^+$-HRMS/MS fragmentation spectrum of 12 (20 CE). 34
Figure S51. ESI$^+$-HRMS m/z envelope of 13. 34
Figure S52. ESI$^+$-HRMS/MS fragmentation spectrum of 13 (20 CE). 34
Figure S53. ESI$^+$-HRMS/MS fragmentation of m/z 329.1500 from Fig. 2b (20 CE). 35
Figure S54. ESI$^+$-HRMS/MS fragmentation spectrum of m/z 439.1773 from Fig. 2b (20 CE). 35
Figure S55. ESI$^+$-HRMS/MS fragmentation spectrum of m/z 345.1447 from Fig. 2b (20 CE). 35
Figure S56. ESI$^+$-HRMS/MS fragmentation spectrum of m/z 329.1500 from Fig. 2b (20 CE). 35
Figure S57. ESI$^+$-HRMS/MS fragmentation spectrum of m/z 315.1339 from Fig. 2b (20 CE). 36
Figure S58. ESI$^+$-HRMS/MS fragmentation spectrum of m/z 363.1707 from Fig. 2b (20 CE). 36
Figure S59. ESI$^+$-HRMS/MS fragmentation spectrum of m/z 347.1758 from Fig. 2b (20 CE). 36
Figure S60. ESI$^+$-HRMS/MS fragmentation spectrum of m/z 361.1907 from Fig. 2b (20 CE). 36
Figure S61. ESI$^+$-HRMS/MS fragmentation spectrum of m/z 271.1442 from Fig. 2b (20 CE). 37
Figure S62. ESI$^+$-HRMS/MS fragmentation spectrum of m/z 287.1393 from Fig. 2b (20 CE). 37
Figure S63. ESI$^+$-HRMS/MS fragmentation spectrum of m/z 285.1601 from Fig. 2b (20 CE). 37
Figure S64. ESI$^+$-HRMS/MS fragmentation spectrum of m/z 301.1553 from Fig. 2b (20 CE). 37
Figure S65. ESI$^+$-HRMS/MS fragmentation spectrum of m/z 321.1593 from Fig. 2b (20 CE). 38
Figure S66. ESI$^+$-HRMS/MS fragmentation spectrum of m/z 264.1248 from Fig. 2b (20 CE). 38
Figure S67. ESI$^+$-HRMS/MS fragmentation spectrum of m/z 222.0600 from Fig. 2b (20 CE). 38
Figure S68. ESI$^+$-HRMS/MS fragmentation spectrum of m/z 246.0616 from Fig. 2b (20 CE). 38
Figure S69. ESI$^+$-HRMS/MS fragmentation spectrum of m/z 204.0618 from Fig. 2b (20 CE). 39
Figure S70. ESI$^+$-HRMS/MS fragmentation spectrum of m/z 246.1120 from Fig. 2b (20 CE). 39
Figure S71. ESI$^+$-HRMS/MS fragmentation spectrum of 12 (10 CE) from in vitro reactions. 39
Figure S72. ESI$^+$-HRMS/MS fragmentation spectrum of 13 (10 CE) from in vitro reactions. 39
Figure S73. ESI$^+$-HRMS m/z envelope of 14 from in vitro reactions. 40
Figure S74. ESI$^+$-HRMS/MS fragmentation spectrum of 14 (10 CE) from in vitro reactions. 40
Figure S75. ESI$^+$-HRMS m/z envelope of 15 from in vitro reactions. 40
Figure S77. ESI$^+$-HRMS m/z envelope of 16 from in vitro reactions. 41

Figure S78. ESI^+-HRMS/MS fragmentation spectrum of 16 (10 CE) from in vitro reactions. *41*

Figure S79. ESI^+-HRMS m/z envelope of 17 from in vitro reactions. *41*

Figure S80. ESI^+-HRMS/MS fragmentation spectrum of 17 (10 CE) from in vitro reactions. *41*

Figure S81. ^{1}H NMR spectrum of 6 (600 MHz, methanol-d_4). *42*

Figure S82. gCOSY spectrum of 6 (600 MHz, methanol-d_4). *43*

Figure S83. gHSQC spectrum of 6 (600 MHz, methanol-d_4). *44*

Figure S84. gHMBC spectrum of 6 (600 MHz, methanol-d_4). *45*

Figure S85. ^{1}H, ^{15}N-gHMBC spectrum of 6 (600 MHz, methanol-d_4). *46*

Figure S86. ^{1}H NMR spectrum of 7 (600 MHz, acetonitrile-d_3). *47*

Figure S87. gCOSY spectrum of 7 (600 MHz, acetonitrile-d_3). *48*

Figure S88. gHSQC spectrum of 7 (600 MHz, acetonitrile-d_3). *49*

Figure S89. gHMBC spectrum of 7 (600 MHz, acetonitrile-d_3). *50*

Figure S90. ^{1}H, ^{15}N-gHMBC spectrum of 7 (600 MHz, acetonitrile-d_3). *51*

Figure S91. ^{1}H NMR spectrum of 9-Me (600 MHz, acetonitrile-d_3). *52*

Figure S92. gCOSY spectrum of 9-Me (600 MHz, acetonitrile-d_3). *53*

Figure S93. gHSQC spectrum of 9-Me (600 MHz, acetonitrile-d_3). *54*

Figure S94. gHMBC spectrum of 9-Me (600 MHz, acetonitrile-d_3). *55*

Figure S95. ^{1}H, ^{15}N-gHMBC spectrum of 9-Me (600 MHz, acetonitrile-d_3). *56*

Figure S96. ^{1}H NMR spectrum of 18 (400 MHz, methanol-d_4). *57*

Figure S97. ^{13}C NMR spectrum of 18 (400 MHz, methanol-d_4). *58*

Figure S98. ^{1}H NMR spectrum of 19 (400 MHz, methanol-d_4). *59*

Figure S99. ^{13}C NMR spectrum of 19 (400 MHz, methanol-d_4). *60*

Figure S100. ^{1}H NMR spectrum of 20 (400 MHz, methanol-d_4). *61*

Figure S101. ^{13}C NMR spectrum of 20 (400 MHz, methanol-d_4). *62*

Figure S102. ^{1}H NMR spectrum of 12 (400 MHz, methanol-d_4). *63*

Figure S103. RNA-Seq volcano plot analysis of genes differentially regulated by pyrazine 6 relative to a DMSO vehicle control. Points marked in blue have a -log(P_{adj}) > 2 and abs(log_2FC) > 1. Points marked in red are related to iron acquisition pathways. *64*

Figure S104. Relative abundances of enterobactin monomer, dimer, and linear trimer between wildtype and ΔnanA K. oxytoca strains when supplemented with Neu5Ac (n = 3). *65*

Table S1. DNA constructs used in this study.

Primer/Cassette	Sequence (5'-3')
Spectinomycin Cassette	cagccaggacagaaatgcctcgacttcgctgctgcccaaggttgccgggtgacgcacaccgtggaaacggatgaaggcacgaacccagtggacataagcctgttcggttcgta<br>agctgtaatgcaaGTAGCGTATGCGCTCACGCAACTGGTCCAGAACCTTGACCGAACGCAGCGGTGGTAACGGCGC<br>AGTGGCGGTTTTCATGGCTTGTTATGACTGTTTTTTTGGGGTACAGTCTATGCCTCGGGCATCCAAGCAGCAA<br>GCGCGTTACGCCGTGGGTCGATGTTTGATGTTATGGAGCAGCAACGATGTTACGCAGCAGGGCAGTCGCCCT<br>AAAACAAAGTTAAACATCATGAGGGAAGCGGTGATCGCCGAAGTATCGACTCAACTATCAGAGGTAGTTGG<br>CGTCATCGAGCGCCATCTCGAACCGACGTTGCTGGCCGTACATTTGTACGGCTCCGCAGTGGATGGCGGCCT<br>GAAGCCACACAGTGATATTGATTTGCTGGTTACGGTGACCGTAAGGCTTGATGAAACAACGCGGCGAGCTTT<br>GATCAACGACCTTTTGGAAACTTCGGCTTCCCCTGGAGAGAGCGAGATTCTCCGCGCTGTAGAAGTCACCAT<br>TGTTGTGCACGACGACATCATTCCGTGGCGTTATCCAGCTAAGCGCGAACTGCAATTTGGAGAATGGCAGCG<br>CAATGACATTCTTGCAGGTATCTTCGAGCCAGCCACGATCGACATTGATCTGGCTATCTTGCTGACAAAAGC<br>AAGAGAACATAGCGTTGCCTTGGTAGGTCCAGCGGCGGAGGAACTCTTTGATCCGGTTCCTGAACAGGATCT<br>ATTTGAGGCGCTAAATGAAACCTTAACGCTATGGAACTCGCCGCCCGACTGGGCTGGCGATGAGCGAAATG<br>TAGTGCTTACGTTGTCCCGCATTTGGTACAGCGCAGTAACCGGCAAAATCGCGCCGAAGGATGTCGCTGCCG<br>ACTGGGCAATGGAGCGCCTGCCGGCCCAGTATCAGCCCGTCATACTTGAAGCTAGACAGGCTTATCTTGGAC<br>AAGAAGAAGATCGCTTGGCCTCGCGCGCAGATCAGTTGGAAGAATTTGTCCACTACGTGAAAGGCGAGATC<br>ACCAAGGTAGTCGGCAAATAAagctttactgagctaataacaggactgctggtaatcgcaggcctttttatttctgca
GFP_KOx_F	GGCCATTAATCACTCACGGACGACTTTTTATTAAGGATTAATAAAATGAGCAAGGGCGAAGAAC
GFP_KOx_R	ACTGTCGCTGAATATTACAAAATCAAGGTGTTATGAGCCTTTATACAGT
Spec_KOx_F	CACCTTGATTTTGTAATATTCAGCGACAGTcagccaggacagaaatgcctcgacttcgct
Spec_KOx_R	TCTGGCGTGAGCGAGGAATAGTTTTTTCCCTGAATGAAATACATAtgcagaaataaaaaggcct
nanAko_KOx_F	GTATAAAGGTACATAGTTAATCGTAAAAGTTCAGATGAGGTCGTTcagccaggacagaaatgcc
nanAko_KOx_R	AGGTAAATCGCTGAGCGGATAGCGTTTTCGCCACCCGACCACCGTtgcagaaataaaaaggcct
leupko_KOx_F	AGTGATATAAAAGCTAAATAATGGAATATGTTAATAACAGGGTTTTcagccaggacagaaatgcc
leupko_KOx_R	CTGGGCCTTCGGTGTGCCAAAAACAGGCGTCAGATAAAAGGGATTGtgcagaaataaaaaggcct
pyrko_KOx_F	CCAGCGGGCGTGAGCCCCGGCGGTTAATTTTGTTCAGCATATCCTcagccaggacagaaatgcc
pyrko_KOx_R	GGCAATTCTGTCTCTTCTGGTAGTCACGACAATGAAGGTAACGCAtgcagaaataaaaaggcct
tdhko_KOx_F	CCCGCATCGGTAAACAACTGGGCGTCATTGCCTGAGGGTGTGAGcagccaggacagaaatgcc
tdhko_KOx_R	AAAAGCGCCCGTGGGCGCTTTTTTACCATCCTGACCGGGGCGGAAtgcagaaataaaaaggcct
yerko_KOx_F	TCATATAAGCAATGCTTTTGGTATGACGTGCCATCAGGAGGAAGAcagccaggacagaaatgcc
yerko_KOx_R	CCATGCGTTGGCGATGGCGTCCGGGAAAAGTCAGTTTATTTCGCGtgcagaaataaaaaggcct

Table S2. ^{1}H and ^{13}C NMR assignments of natural **6** (600 MHz, methanol-d_4).

	**6**	
Position	δ_H, mult. (J in Hz)	δ_C
1		154.80
2	6.65, d (8.59), 4H	114.90 (CH, CH_3)
3	6.97, d (8.59) 4H	129.35 (CH, CH_3)
4		129.26
5	4.07, s, 4H	38.34 (CH_2)
6		151.05
7		150.00
8	2.99, t (7.11), 4H	27.70 (CH_2)
9	2.61, t (7.11), 4H	31.12 (CH_2)
10		175.43

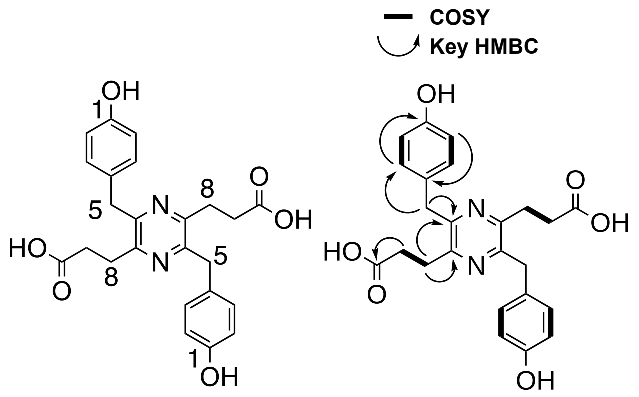

Table S3. ^{1}H and ^{13}C NMR assignments of natural **7** (600 MHz, acetonitrile-d_3).

	**7**	
Position	δ_H, mult. (J in Hz)	δ_C
1		173.32
2	2.67, t (6.93), 2H	30.53 (CH_2)
3	3.02, t (6.93) 2H	27.70 (CH_2)
4		151.05
5		152.46
6	4.24, s, 2H	39.71 (CH_2)
7		138.05
8	7.20 d (7.46), 2H	128.77 (CH, CH_3)
9	7.28, t (7.55), 2H	128.57 (CH, CH_3)
10	7.22, t (7.41), 1H	126.52 (CH, CH_3)
11	8.33, s, 1H	141.17 (CH, CH_3)
12		150.23
13	2.50, s, 3H	19.69 (CH, CH_3)

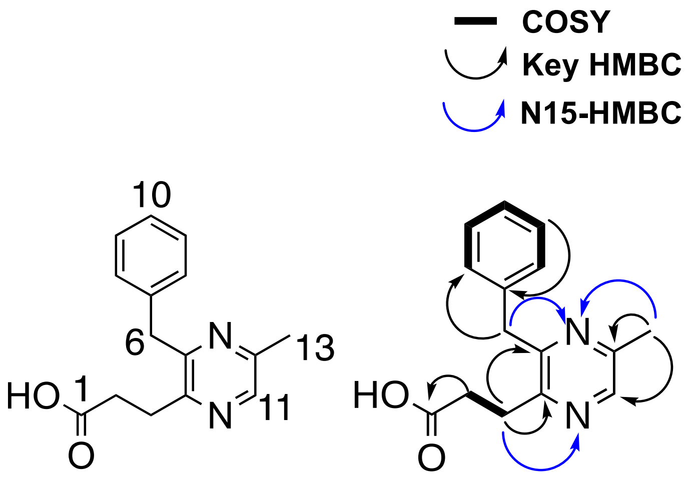

Table S4. ^{1}H and ^{13}C NMR assignments of isolated **9-Me** (600 MHz, acetonitrile-d_3). Me substituent arose under methanolic isolation conditions of natural metabolite **9**.

	**9-Me**	
Position	δ_H, mult. (J in Hz)	δ_C
1<br>1'		173.43<br>173.43
2<br>2'	2.68, m, 2H<br>2.70, m, 2H	30.63 (CH_2)<br>30.63 (CH_2)
3<br>3'	3.00, m, 2H<br>3.02, m, 2H	27.70 (CH_2)<br>27.70 (CH_2)
4<br>4'		149.76<br>149.76
5<br>5'		150.47<br>150.47
6<br>6'	4.13, s, 2H<br>4.14, s, 2H	39.81 (CH_2)<br>39.81 (CH_2)
7<br>7'		138.75<br>138.75
8<br>8'	7.24, m, 2H<br>7.24, m, 2H	128.77 (CH, CH_3)<br>128.77 (CH, CH_3)
9<br>9'	7.27, m, 2H<br>7.27, m, 2H	128.46 (CH, CH_3)<br>128.46 (CH, CH_3)
10<br>10'	7.20, m, 1H<br>7.20, m, 1H	126.33 (CH, CH_3)<br>126.33 (CH, CH_3)
11	3.56, s, 3H	51.04 (CH, CH_3)

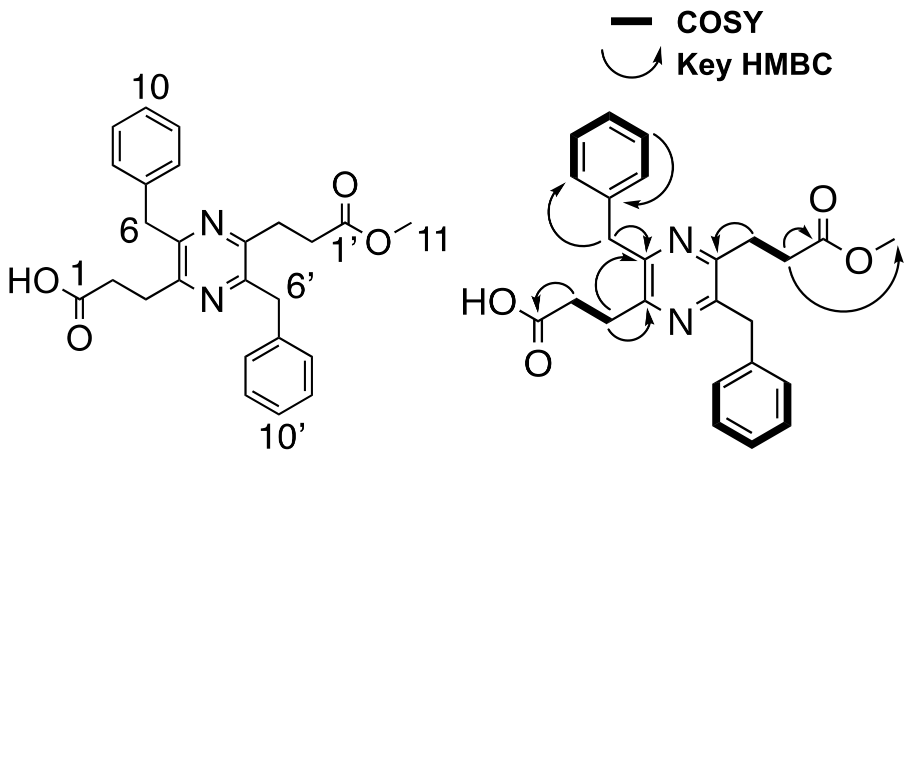

Table S5. ^{1}H and ^{13}C NMR assignments of synthetic **18** (400 MHz, methanol-d_4).

	**18**	
Position	δ_H, mult. (J in Hz)	δ_C
1	4.84 (overlapped with HDO)	62.09
2	3.01, dd (5.57, 13.49)<br>2.80, broad dd	38.85
3		138.63
4	7.23, m	129.39
5	7.24, m	130.39
6	7.27, m	127.75
7		157.66
1’		157.64
2’		80.51
3’	1.38, s	28.66
4’	3.16, s	53.65
5’	3.72, s	62.02

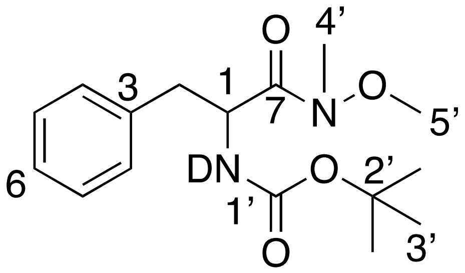

Table S6. ^{1}H and ^{13}C NMR assignments of synthetic **19** (400 MHz, methanol-d_4).

	**19**	
Position	δ_H, mult. (J in Hz)	δ_C
1	4.34, m	62.32
2	3.12, dd (5.46, 13.89)<br>2.82, dd (9.14, 13.89)	37.54
3		138.82
4	7.24, m	129.45
5	7.25, m	130.30
6	7.31, m	127.64
7		209.55
1'		157.85
2'		80.57
3'	1.41, s	28.66
4'	2.30 broad t	40.00
5'	2.64, dt (7.38, 34.31)	28.49
6'	5.82, m	138.43
7'	5.05, m<br>5.01, m	115.53

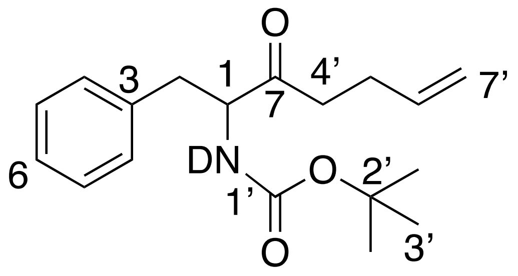

Table S7. ^{1}H and ^{13}C NMR assignments of synthetic **20** (400 MHz, methanol-d_4).

	**20**	
Position	δ_H, mult. (J in Hz)	δ_C
1	4.36, m	62.38
2	3.18, dd (5.07, 14.13)<br>2.84, m	37.41
3		138.96
4	7.24, m	129.42
5	7.26, m	130.33
6	7.30, m	127.59
7		210.09
1’		157.88
2’		80.59
3’	1.40, s	28.66
4’	2.84, m	35.42
5’	2.56, t (6.30)	28.40
6’		176.29

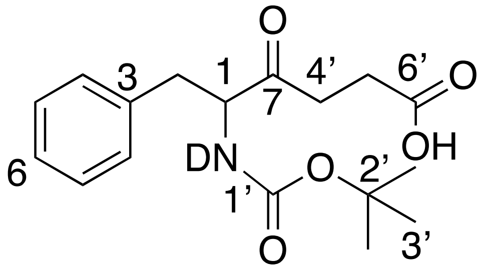

Figure S1. **a**. Proposed structures of compounds from the leupeptin molecular network. MS^2 analysis of proposed non-adduct features is presented in the Figure S9. **b**. Architecture of the *nan* operon from *K. oxytoca*.

a

Feature (*m/z*)	Proposed Structure	Theoretical $[M+H]^+$ (*m/z*)
445.3137	**1** + H_2O	445.3133
459.3269	**1** + MeOH	459.3289
443.2986	*N*-Acetyl Leu-Leu-Arg	443.2976
367.2690	Daughter ion of **1**	not determined
413.2861	*N*-Acetyl Leu-Val-Arginal	413.2871
431.2964	*N*-Acetyl Leu-Val-Arginal + H_2O	431.2976
395.2753	*N*-Acetyl Leu-Val-Arginal - H_2O	395.2765
429.2828	*N*-Acetyl Leu-Val-Arg	429.2820
417.3094	**2** + H_2O	417.3071
381.2850	**2** - H_2O	381.2860
431.3241	**2** + MeOH	431.3228
415.2913	*N*-Acetyl Leu-Leu-Lys	415.2915
385.2814	*N*-Acetyl Leu-Val-Lysinal	385.2809
403.2922	*N*-Acetyl Leu-Val-Lysinal + H_2O	403.2915
401.2765	*N*-Acetyl Leu-Val-Lys	401.2758
287.1946	*N*-Acetyl Leu-Leu	287.1965
273.1788	*N*-Acetyl Leu-Val	273.1809

b

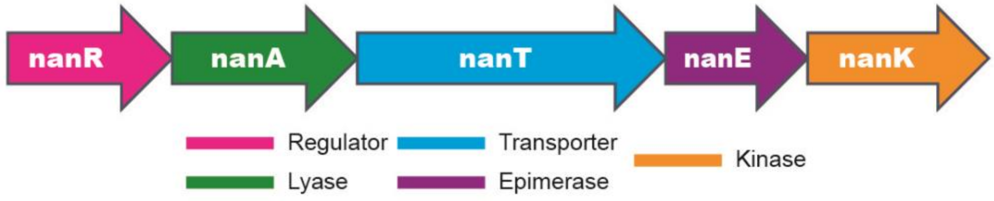

Figure S2. **a**. Operon architecture of the yersiniabactin biosynthetic gene cluster. **b**. Relative production of pyrazines **5-9** and yersiniabactins **10** and **11** in wildtype *K. oxytoca* when supplemented with various sugars. Data are represented as mean values +/- SD from n = 2 biological replicates.

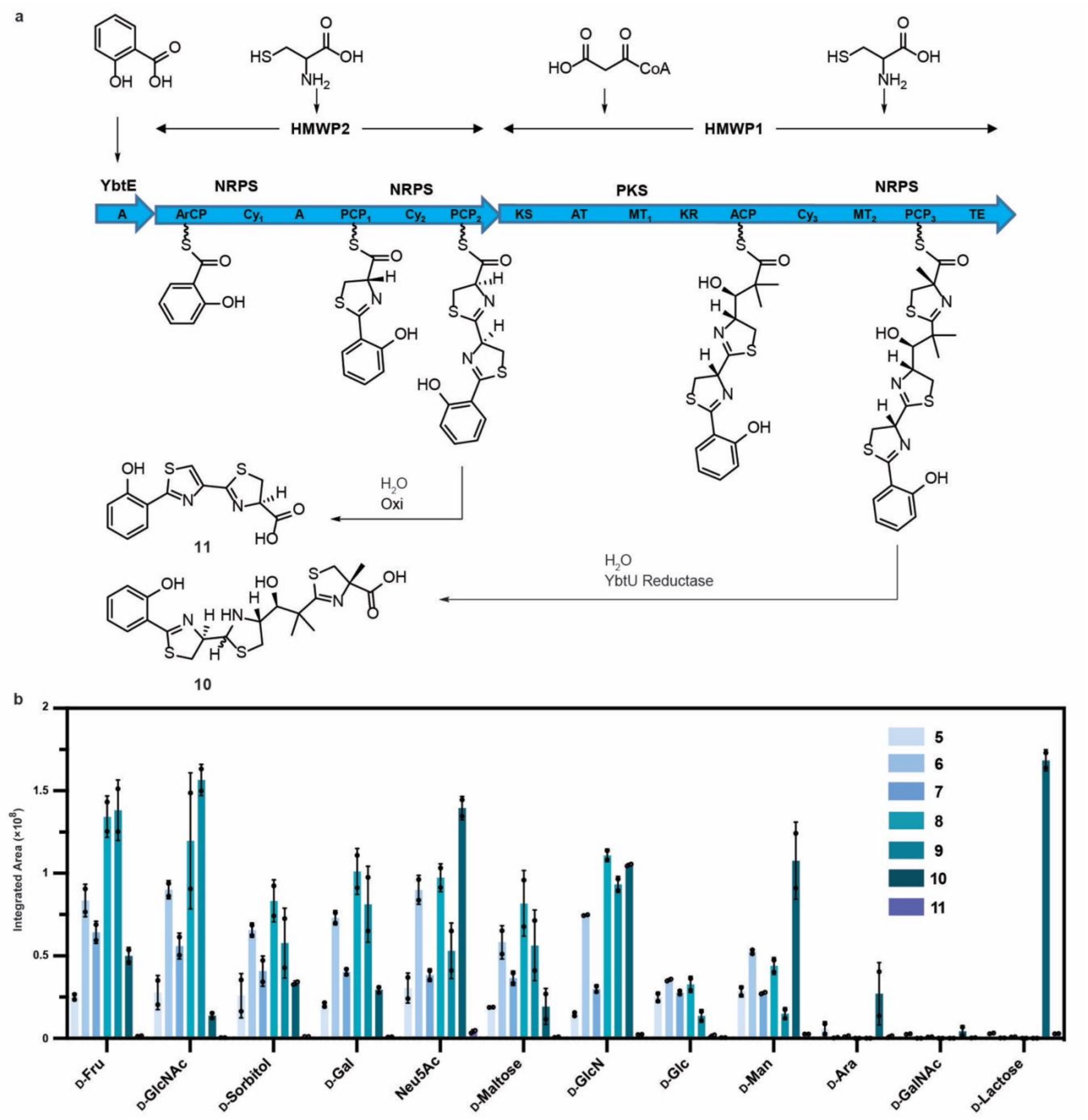

Figure S3. **a**. Biosynthesis of **12** via the PLP-dependent enzyme PqrA *en route* to the production of perquinolines.[53] **b**. HPLC-QTOF-MS extracted ion chromatograms of pyrazines **5-9** from wildtype *K. oxytoca*, Δ*pyr K. oxytoca*, and medium control samples. Compounds **5-9** were not detected in Δ*pyr K. oxytoca* or in growth medium controls. **c**. HPLC-QTOF-MS extracted ion chromatograms of yersiniabactin **10** from wildtype *K. oxytoca*, Δ*pyr K. oxytoca*, and medium control samples. **d.** Relative abundances of pyrazines **5-9** between wildtype and Δ*tdh K. oxytoca* strains supplemented with D-Galactose (n = 3). Pyrazines were not detected in the growth medium control samples. Statistical analyses performed through an unpaired two-tailed t-test; n.s. indicates a non-significant difference, * indicates $p < 0.05$; ** indicates $p < 0.01$, and *** indicates $p < 0.001$. For panels b and c, representative traces of three biological replicates (n = 3) are shown. Data are represented as mean values +/- SD.

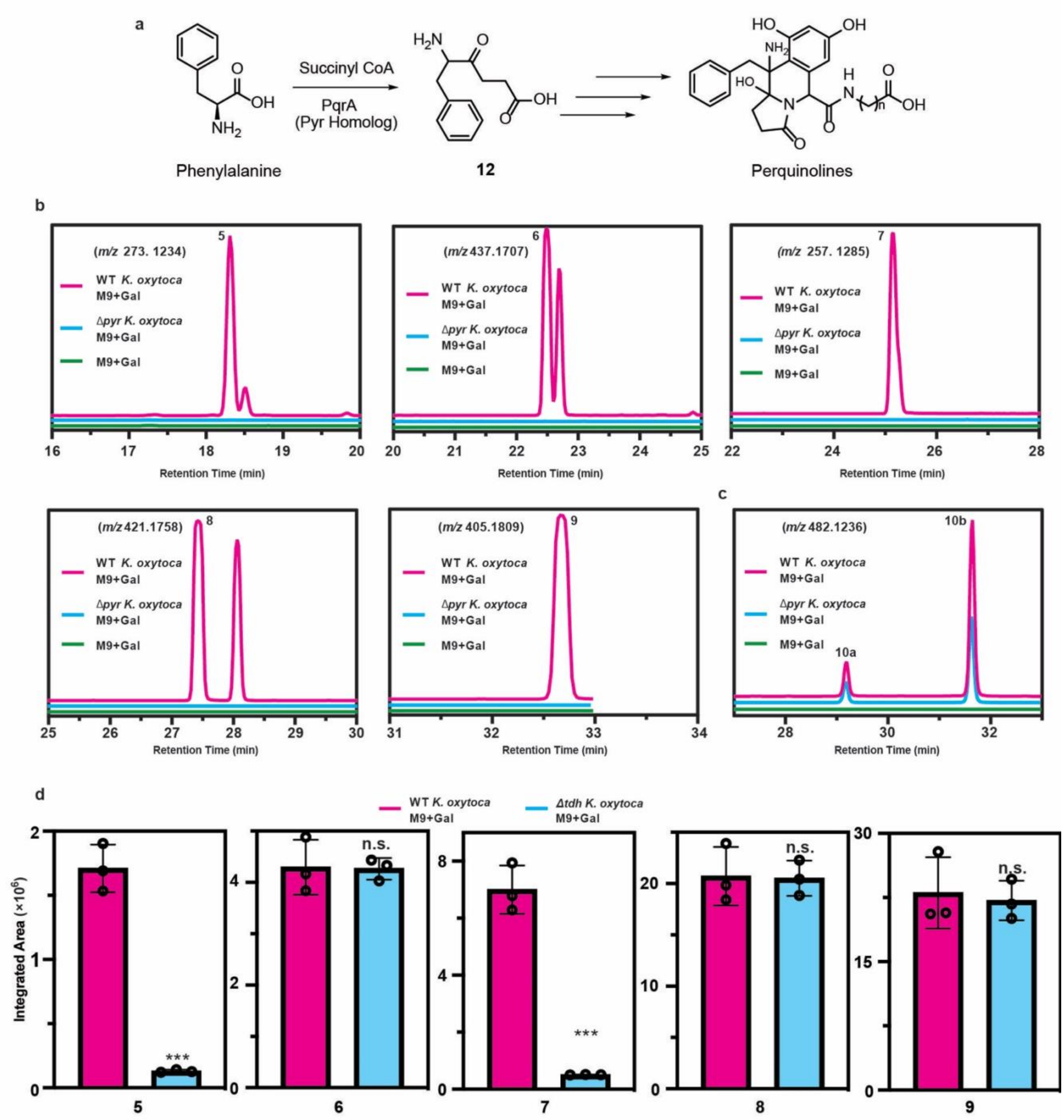

Figure S4. Stereochemical studies of the *K. oxytoca* Pyr protein. **a**. HPLC-QTOF-MS total ion chromatograms of the *in vitro* Pyr reaction with either L- or D-Phe (top left) or L- or D-Tyr (bottom left). Reactions were supplemented with purified Pyr, succinyl CoA, PLP, and appropriate amino acid. Representative traces of three biological replicates (n = 3) are shown. Relative abundances of compounds **12**, **14**, and **16** as a function of L- or D-Phe are shown in the top right and relative abundances of compounds **13**, **15**, and **17** as a function of L- or D-Tyr are shown in the bottom right. The left axes plot the integrated areas of the L-isomer substrates and the right axes plot the integrated areas for the D-isomer substrates. **b**. Proposed chemical structures of compounds **12-17**. **c.** Synthetic route towards **12**. Synthesis was performed separately with each stereoisomer of *N*-Boc-(L- or D-)phenylalanine. **d**. Marfey's analysis of functionalized (Func.) synthetic **12*S*** and **12*R*** against functionalized *K. oxytoca* extract. Non-functionalized (Raw) extract is shown as a negative control. Data are represented as mean values +/- SD.

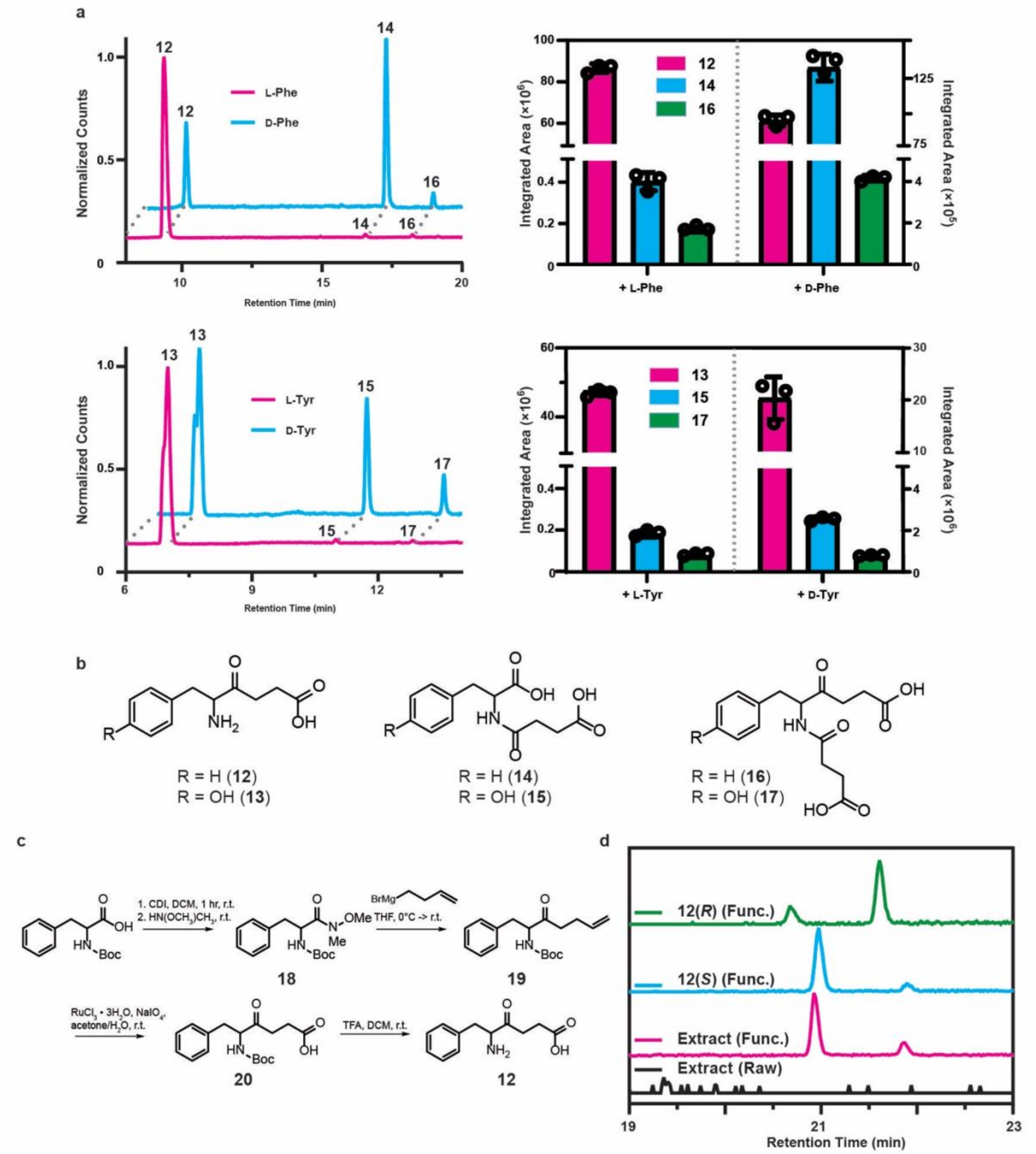

Figure S5. ESI^{+}-HRMS *m/z* envelope of **1**.

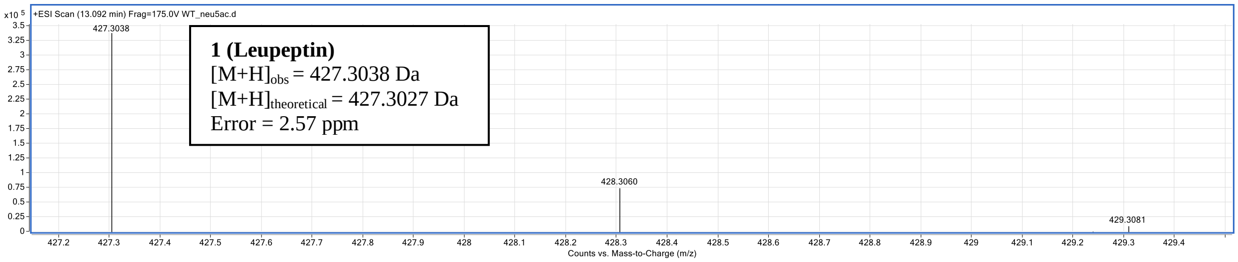

Figure S6. ESI^{+}-HRMS/MS fragmentation spectrum of **1** (20 CE).

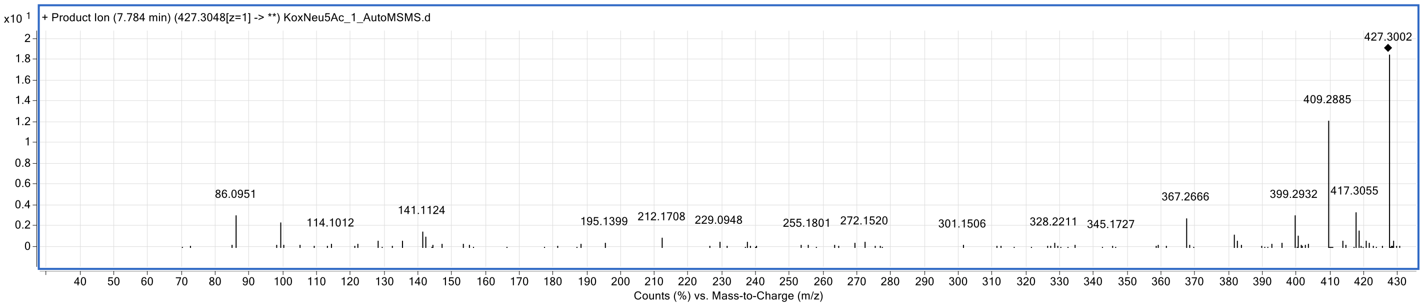

Figure S7. ESI^{+}-HRMS *m/z* envelope of **2**.

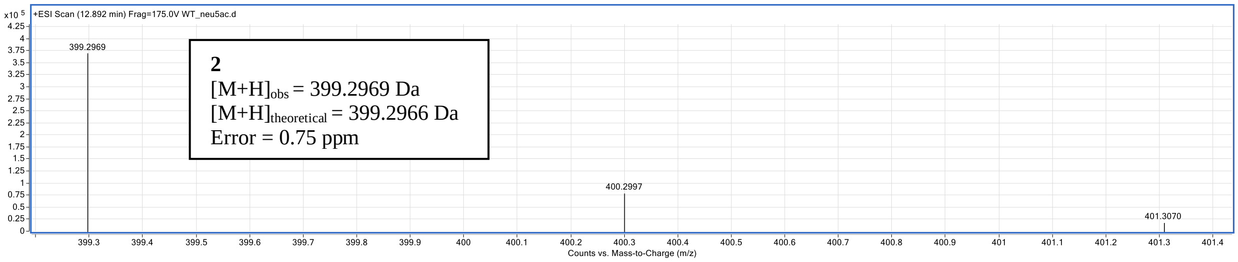

Figure S8. ESI^{+}-HRMS/MS fragmentation spectrum of **2** (20 CE).

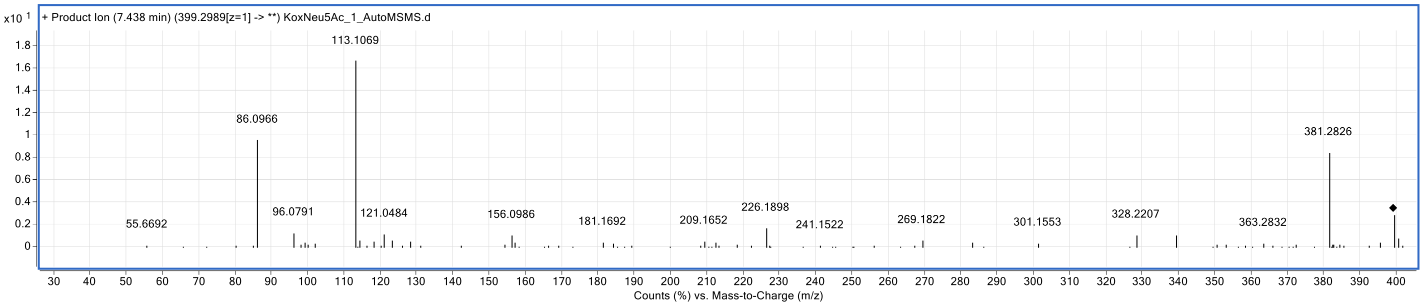

Figure S9. ESI$^+$-HRMS *m/z* envelope of **3**.

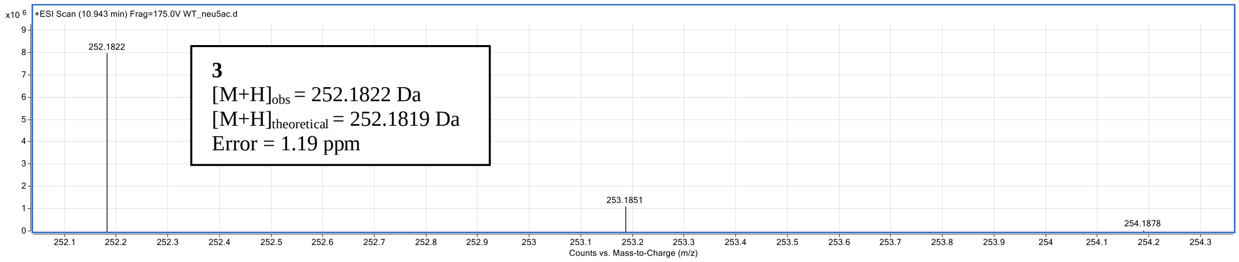

Figure S10. ESI$^+$-HRMS/MS fragmentation spectrum of natural **3** (20 CE).

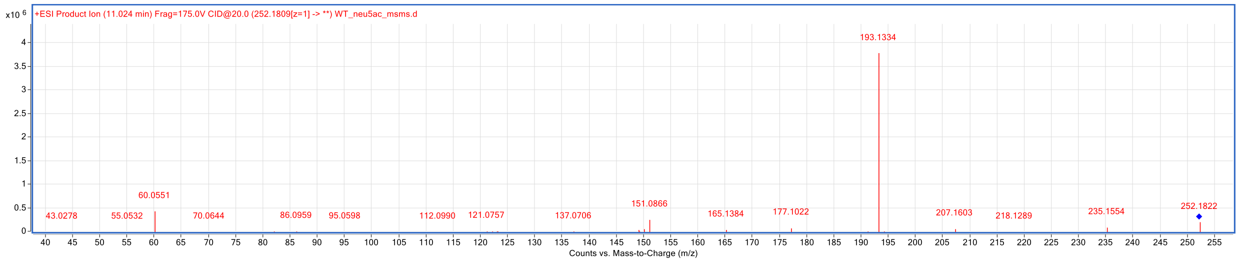

Figure S11. ESI$^+$-HRMS/MS fragmentation spectrum of synthetic **3** (20 CE).

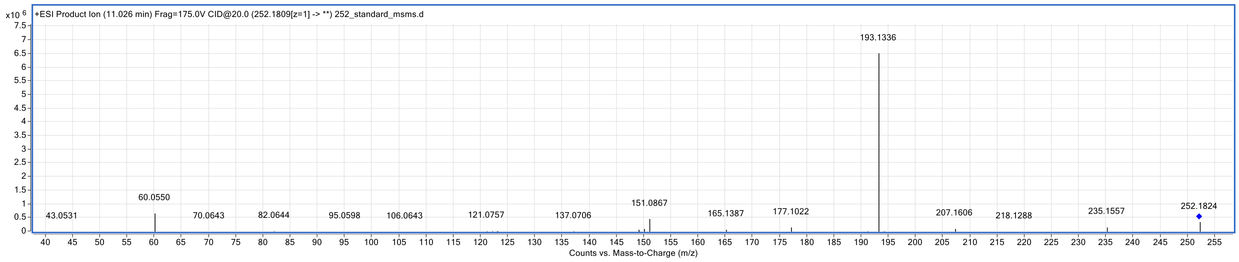

Figure S12. ESI$^+$-HRMS *m/z* envelope of **4**.

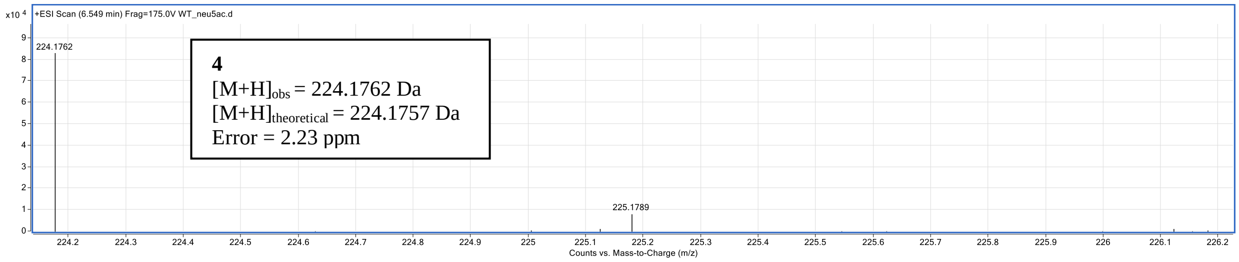

Figure S13. Proposed structures of features from the molecular network in **Fig. 1c**.

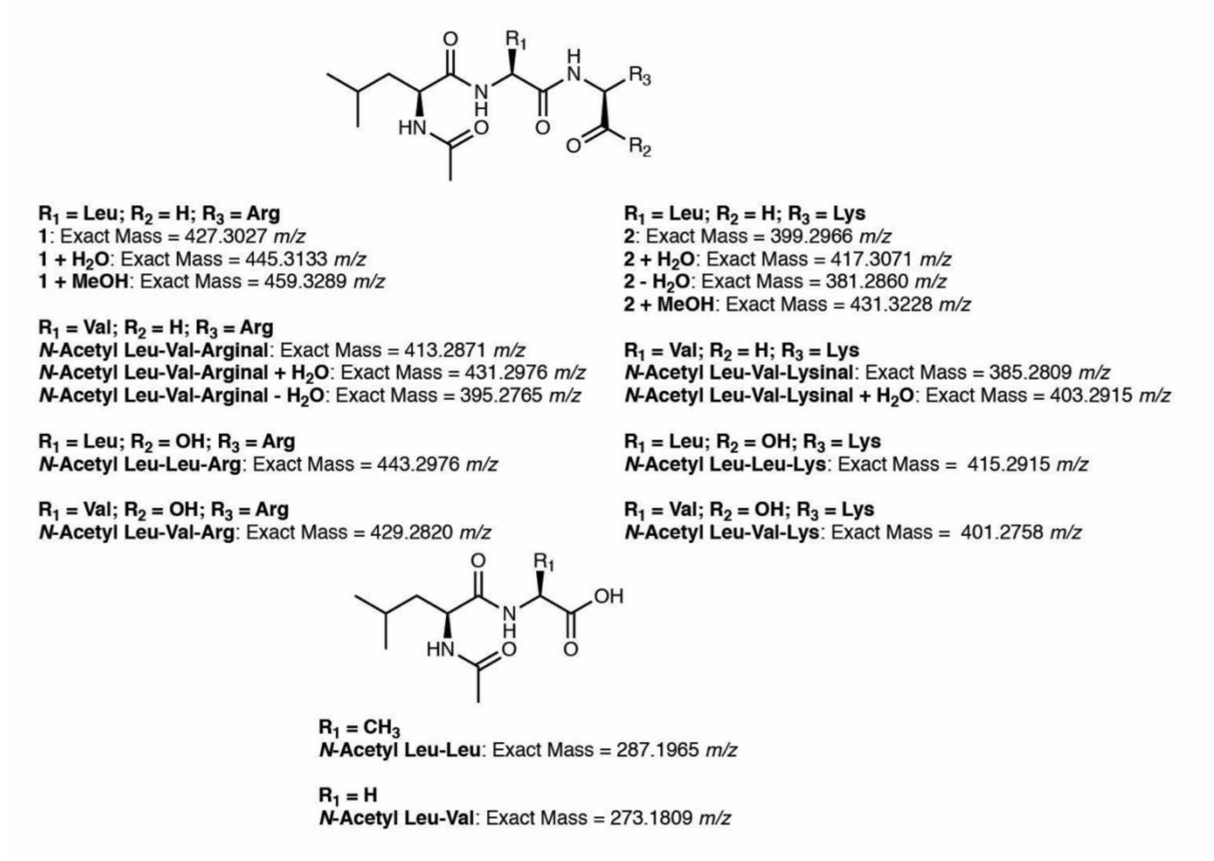

Figure S14. ESI$^+$-HRMS/MS fragmentation spectrum of *m/z* 395.2753 (*N*-Acetyl Leu-Val-Arginal – H_2O) from **Fig. 1c** (20 CE).

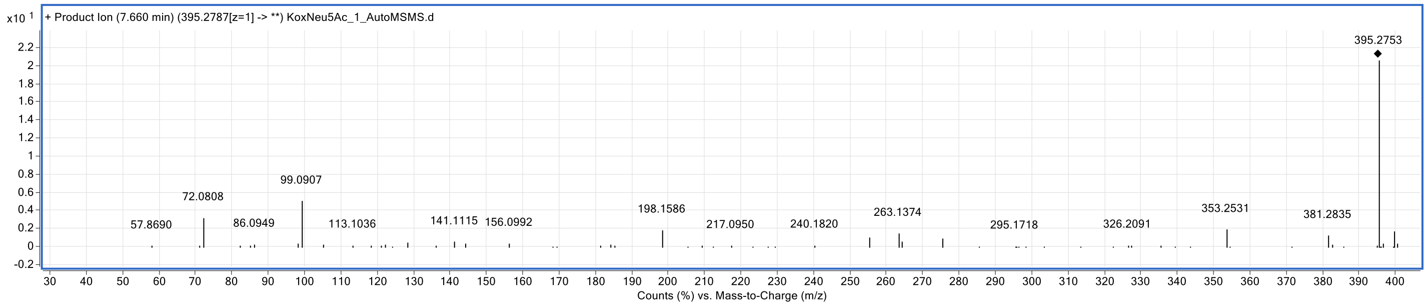

Figure S15. ESI[+]-HRMS/MS fragmentation spectrum of *m/z* 431.2964 (*N*-Acetyl Leu-Val-Arginal + H_2O) from **Fig. 1c** (20 CE).

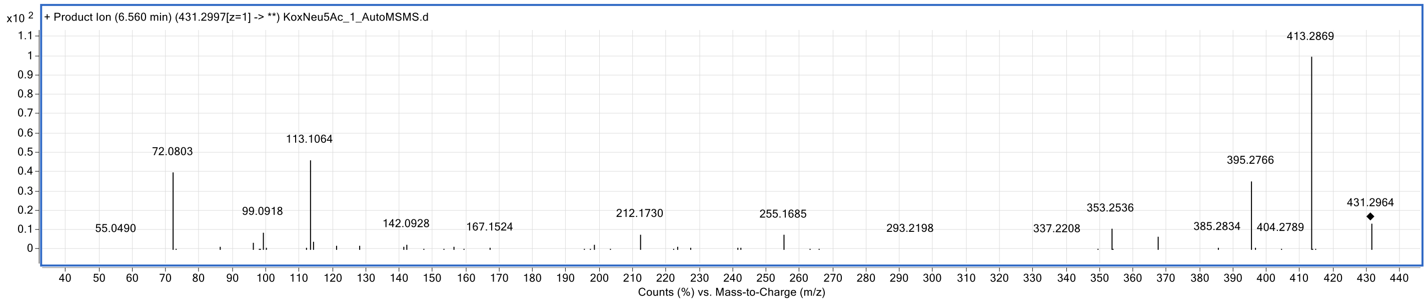

Figure S16. ESI[+]-HRMS/MS fragmentation spectrum of *m/z* 413.2861 (*N*-Acetyl Leu-Val-Arginal) from **Fig. 1c** (20 CE).

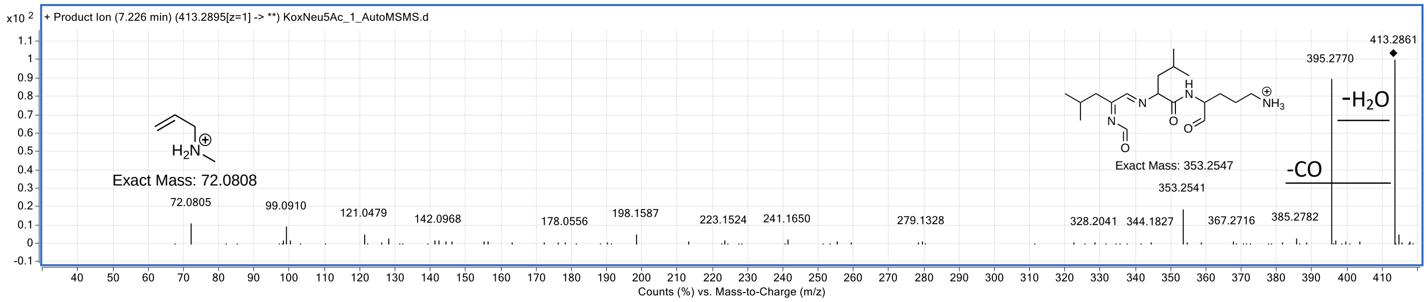

Figure S17. ESI[+]-HRMS/MS fragmentation spectrum of *m/z* 445.3137 (**1** + H_2O) from **Fig. 1c** (20 CE).

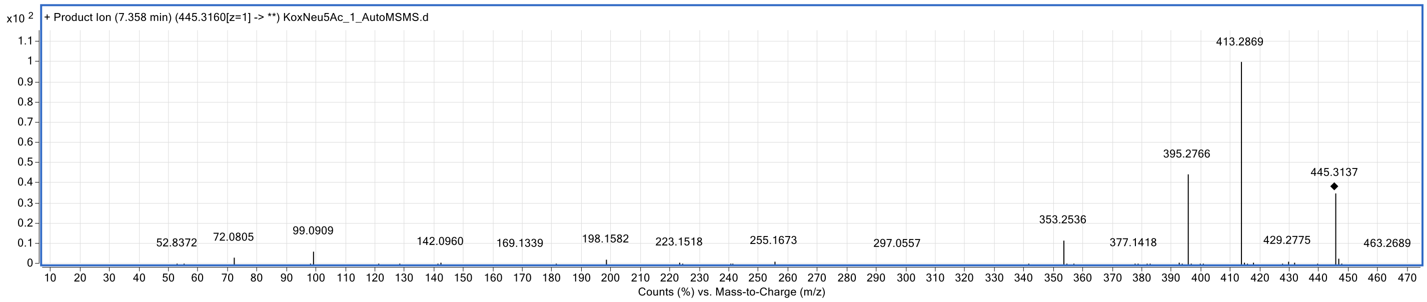

Figure S18. ESI[+]-HRMS/MS fragmentation spectrum of *m/z* 385.2814 (*N*-Acetyl Leu-Val-Lysinal) from **Fig. 1c** (20 CE).

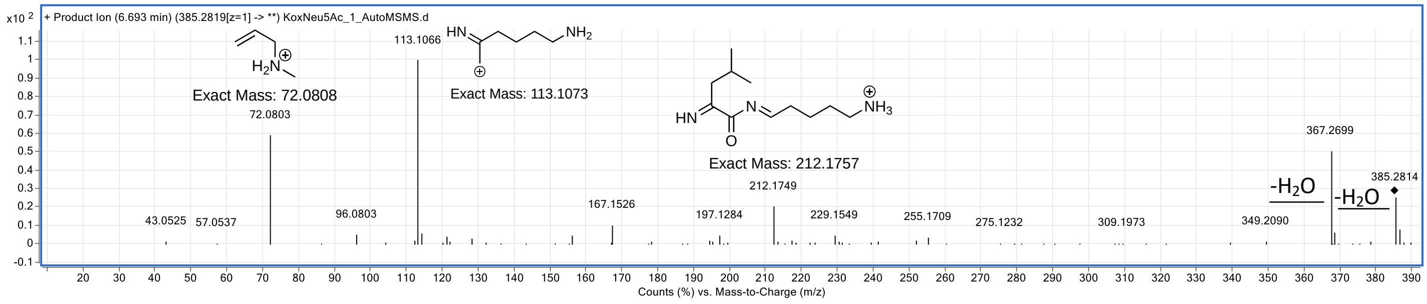

Figure S19. ESI$^+$-HRMS/MS fragmentation spectrum of *m/z* 459.3269 (**1** + MeOH) from **Fig. 1c** (20 CE).

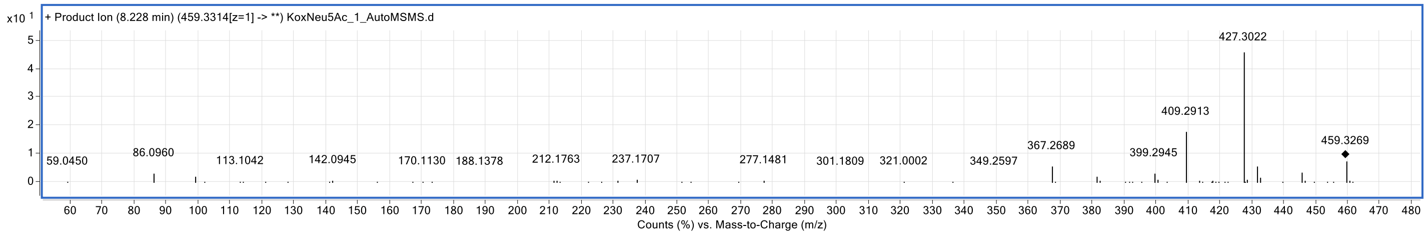

Figure S20. ESI$^+$-HRMS/MS fragmentation spectrum of *m/z* 403.2922 (*N*-Acetyl Leu-Val-Lysinal + H_2O) from **Fig. 1c** (20 CE).

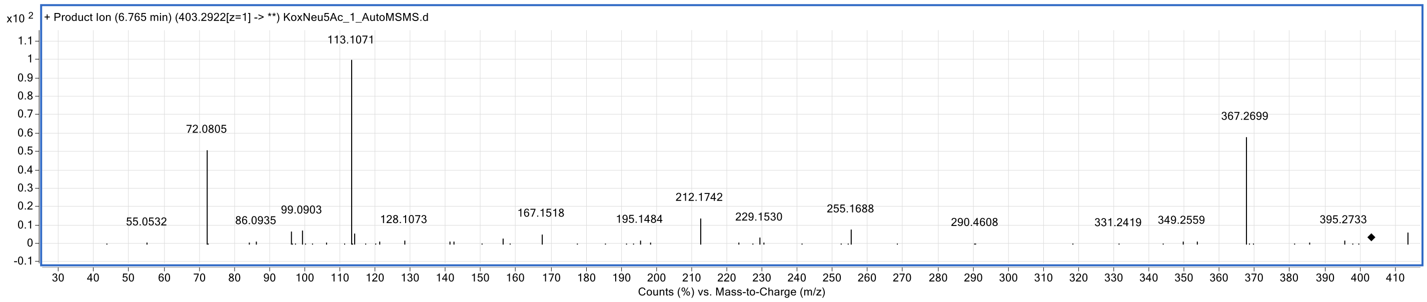

Figure S21. ESI$^+$-HRMS/MS fragmentation spectrum of *m/z* 431.3241(**2** + MeOH) from **Fig. 1c** (20 CE).

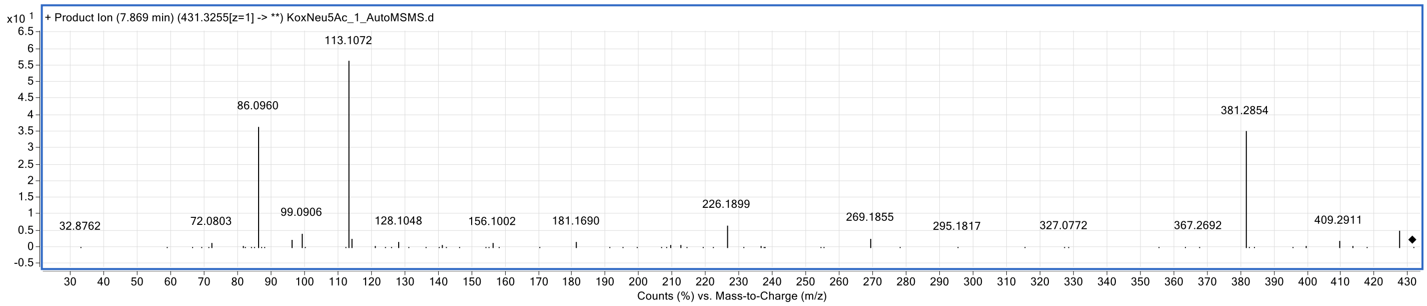

Figure S22. ESI$^+$-HRMS/MS fragmentation spectrum of *m/z* 417.3094 (**2** + H_2O) from **Fig. 1c** (20 CE).

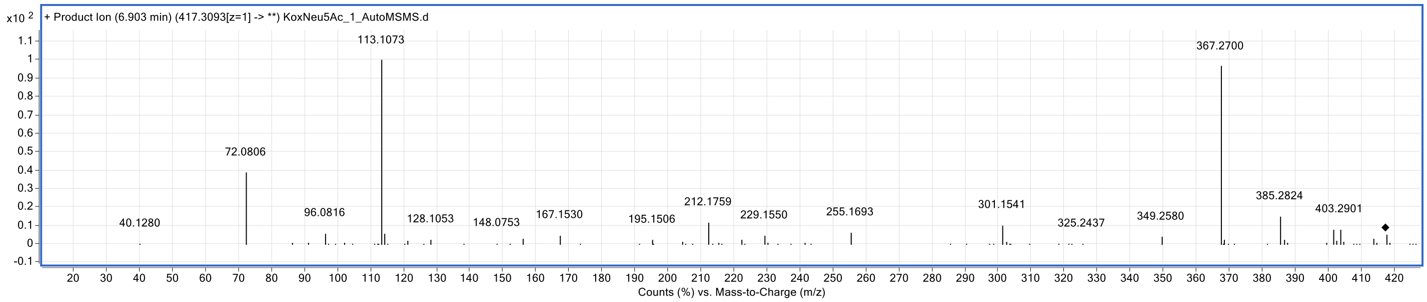

Figure S23. ESI$^+$-HRMS/MS fragmentation spectrum of *m/z* 367.2690 (Daughter Ion of **1**) from **Fig. 1c** (20 CE).

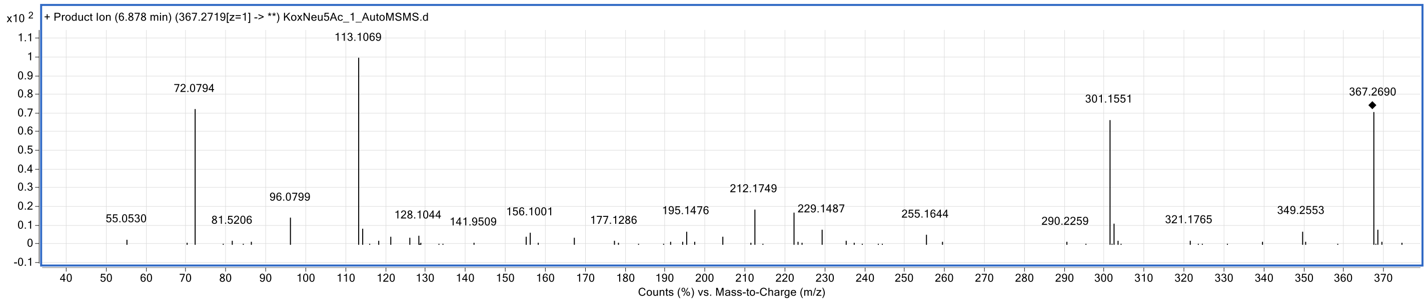

Figure S24. ESI$^+$-HRMS/MS fragmentation spectrum of *m/z* 381.2850 (**2** – H_2O) from **Fig. 1c** (20 CE).

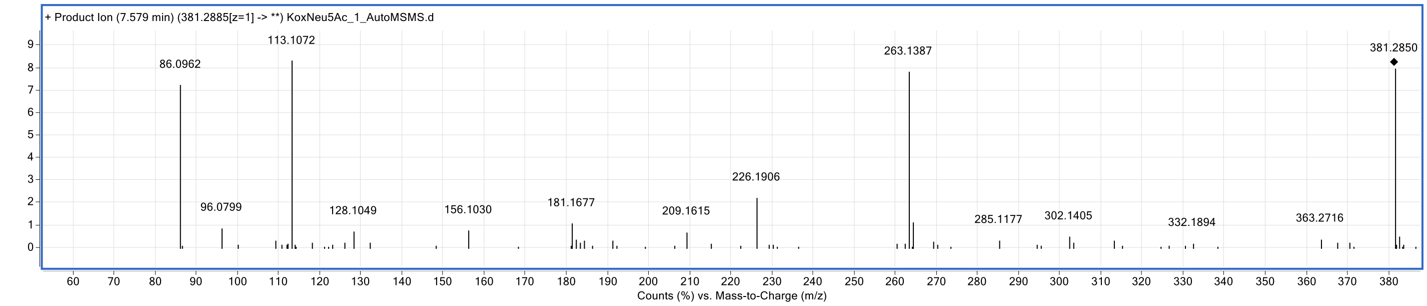

Figure S25. ESI$^+$-HRMS/MS fragmentation spectrum of *m/z* 443.2986 (*N*-Acetyl Leu-Leu-Arg) from **Fig. 1c** (20 CE).

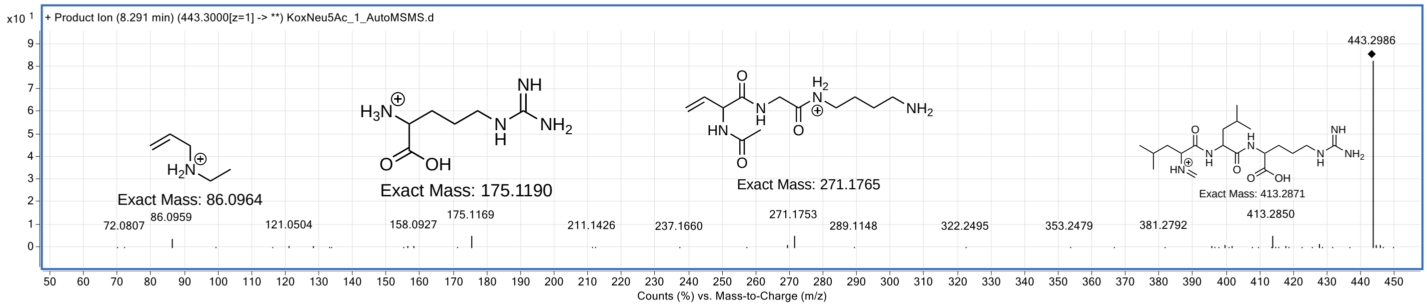

Figure S26. ESI$^+$-HRMS/MS fragmentation spectrum of *m/z* 429.2828 (*N*-Acetyl Leu-Val-Arg) from **Fig. 1c** (20 CE).

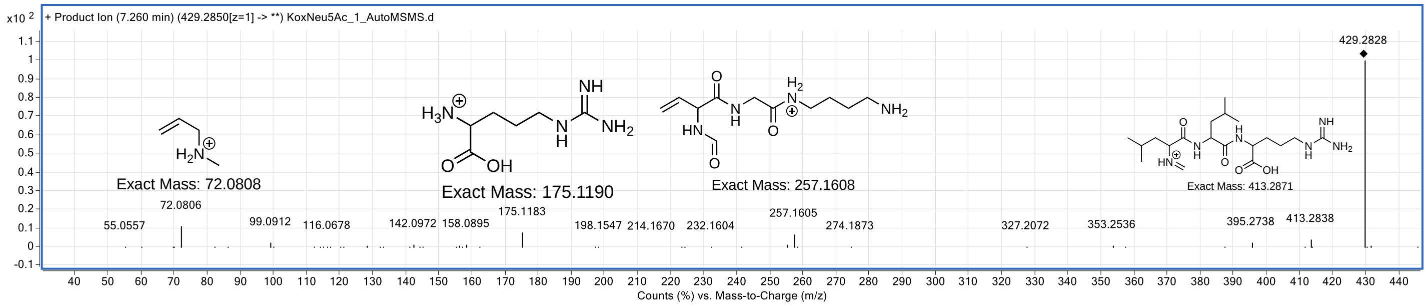

Figure S27. ESI$^+$-HRMS/MS fragmentation spectrum of *m/z* 401.2765 (*N*-Acetyl Leu-Val-Lys) from **Fig. 1c** (20 CE).

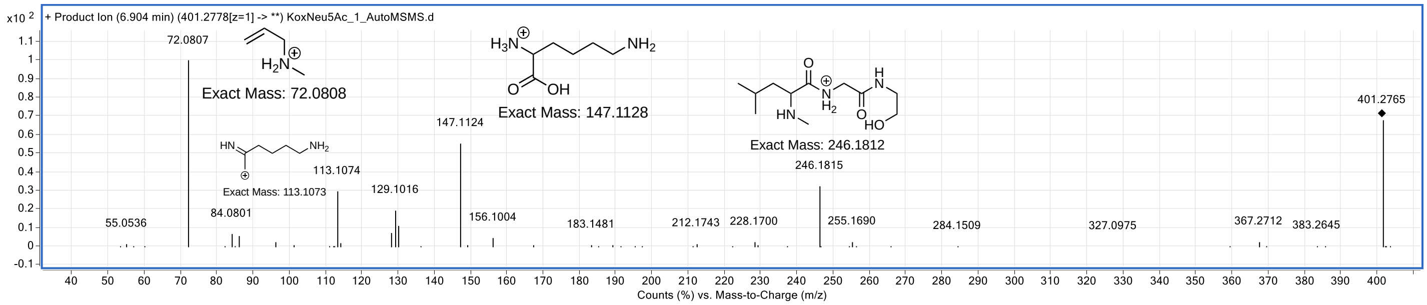

Figure S28. ESI$^+$-HRMS/MS fragmentation spectrum of *m/z* 415.2913 (*N*-Acetyl Leu-Leu-Lys) from **Fig. 1c** (20 CE).

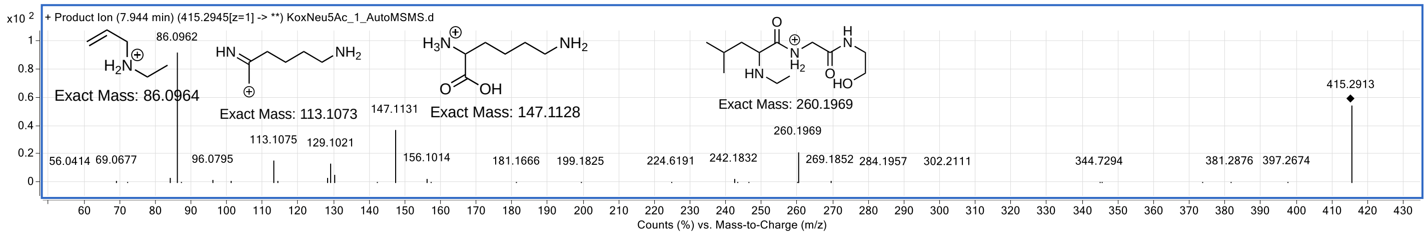

Figure S29. ESI$^+$-HRMS/MS fragmentation spectrum of *m/z* 287.1946 (*N*-Acetyl Leu-Leu) from **Fig. 1c** (20 CE).

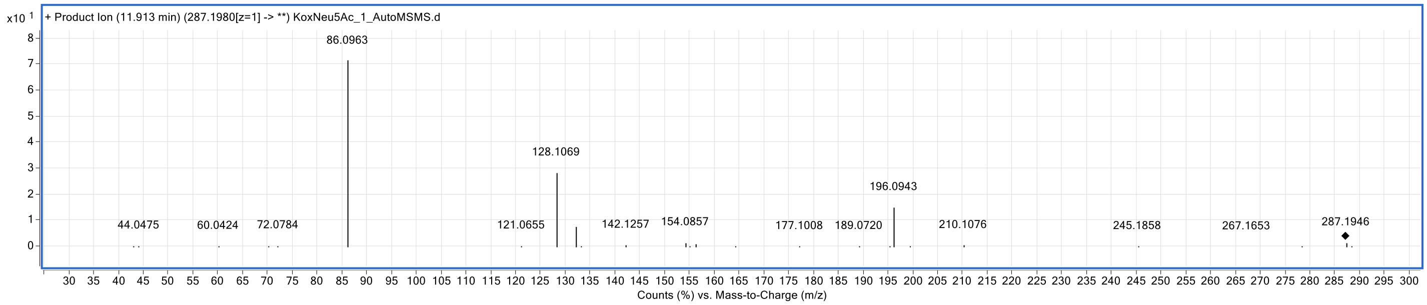

Figure S30. ESI$^+$-HRMS/MS fragmentation spectrum of *m/z* 273.1788 (*N*-Acetyl Leu-Val) from **Fig. 1c** (20 CE).

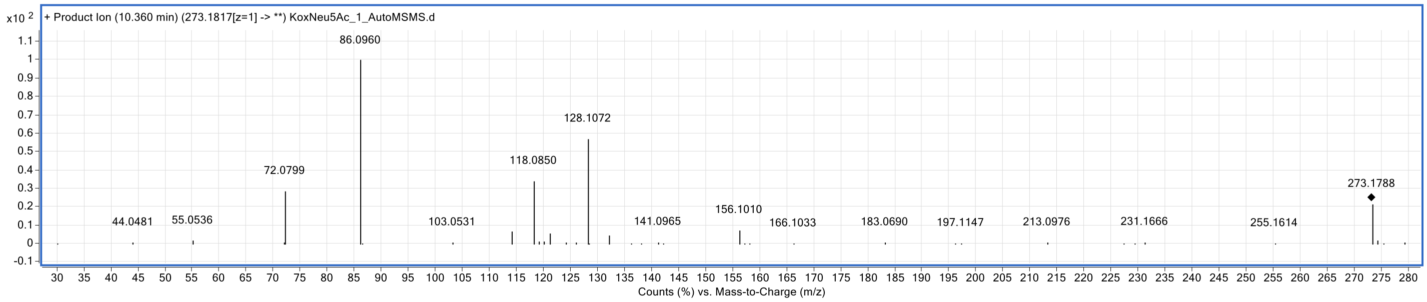

Figure S31. ESI$^+$-HRMS analysis showing Neu5Ac (top), but not Neu5Gc (bottom), can induce leupeptin **1** production.

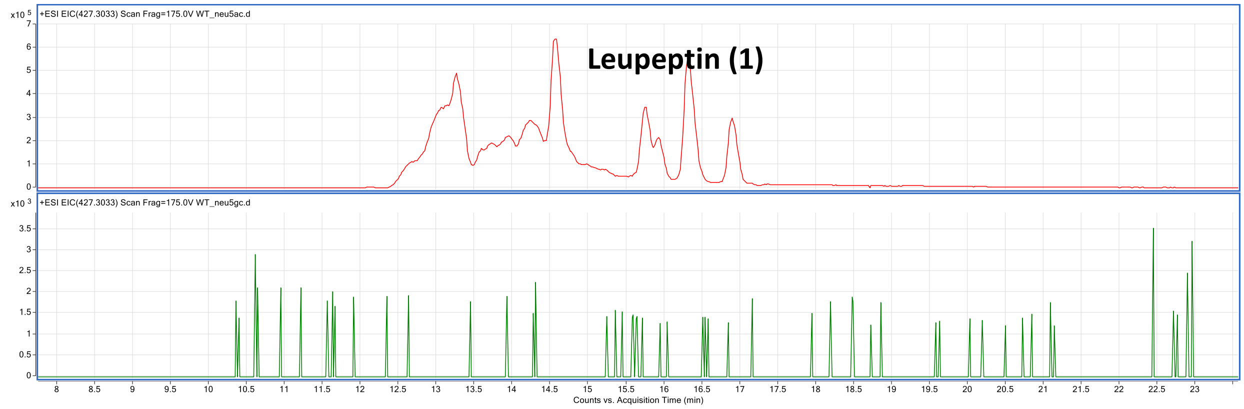

Figure S32. Mucin-mediated production of leupeptins **1** and **2** measured via ESI$^+$-HRMS.

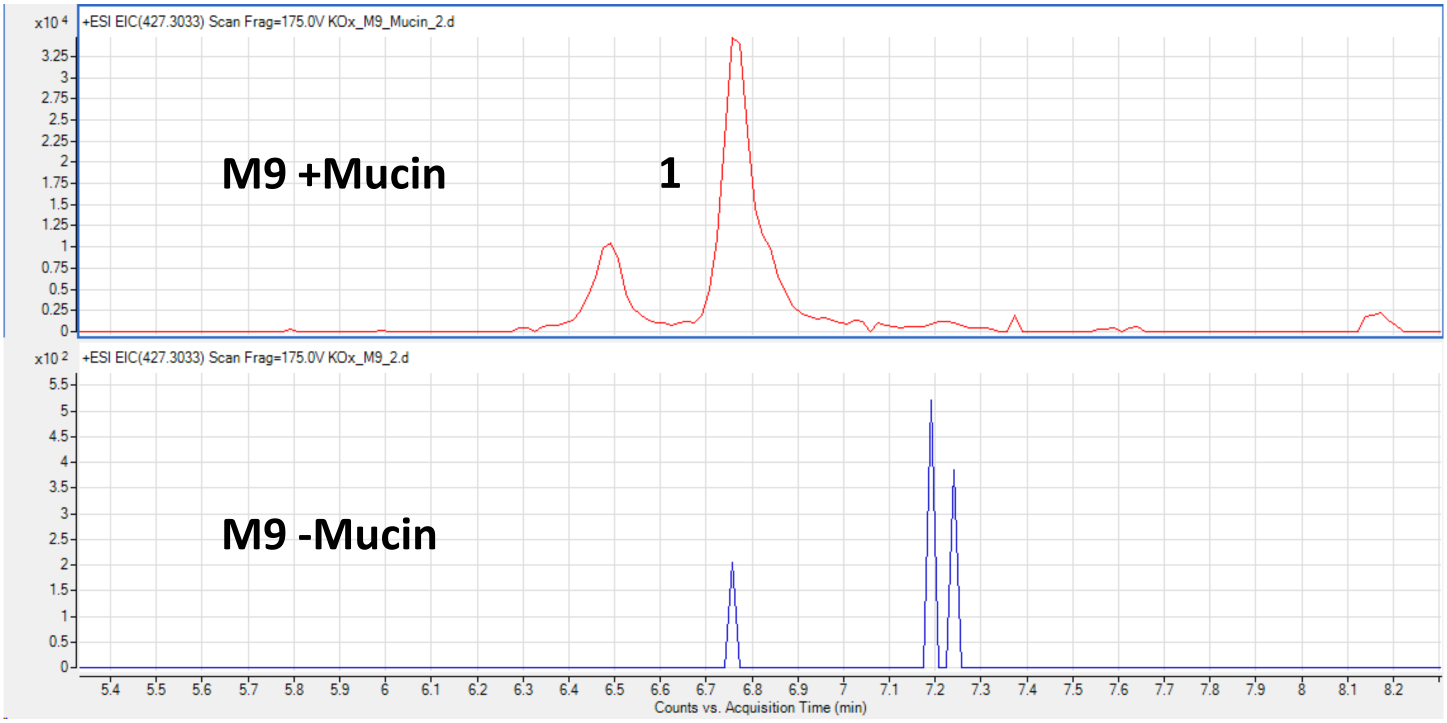

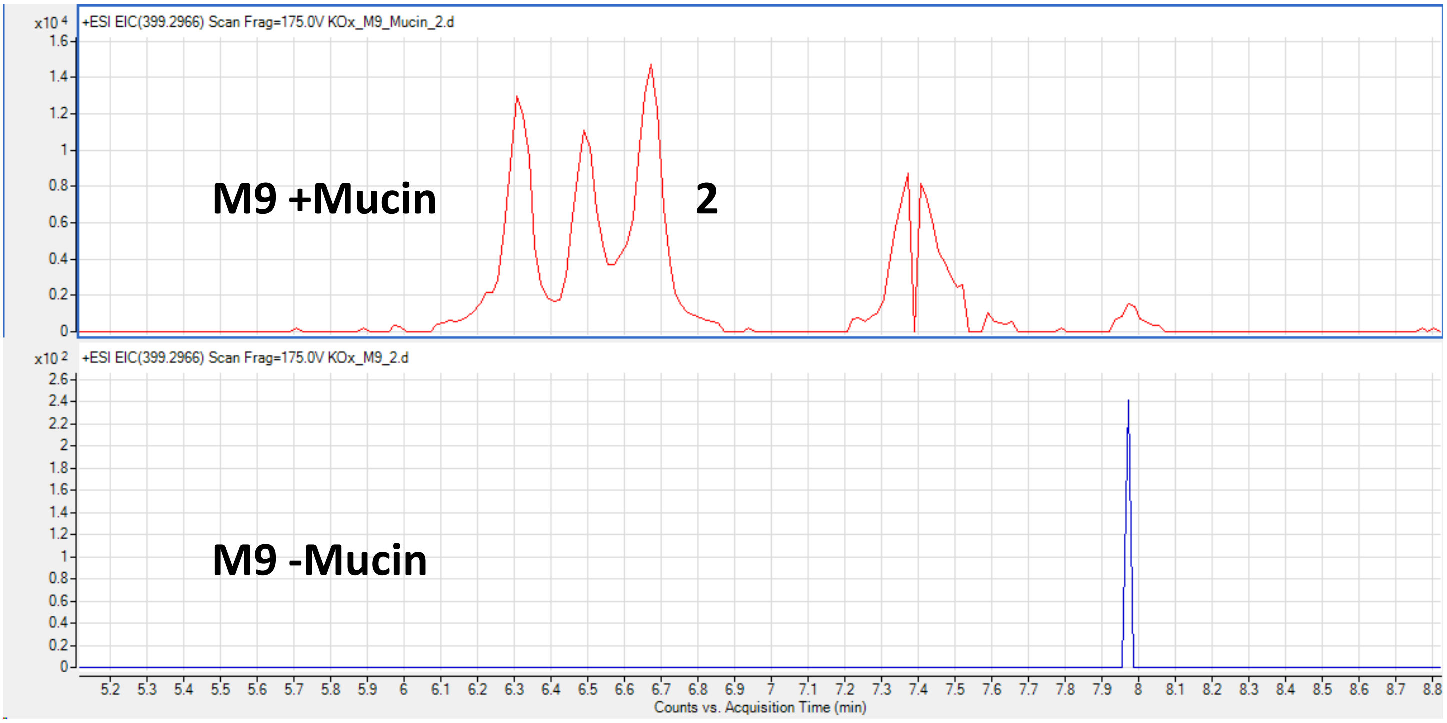

x10 4
+ESI EIC(399.2966) Scan Frag=175.0V KOx_M9_Mucin_2.d
M9 +Mucin
2
x10 2
+ESI EIC(399.2966) Scan Frag=175.0V KOx_M9_2.d
M9 -Mucin
Counts vs. Acquisition Time (min)

Figure S33. The *nanA* mutant cannot produce leupeptin **1** regardless of mucin or Neu5Ac supplementation. The symbol n.d. stands for "not detected."

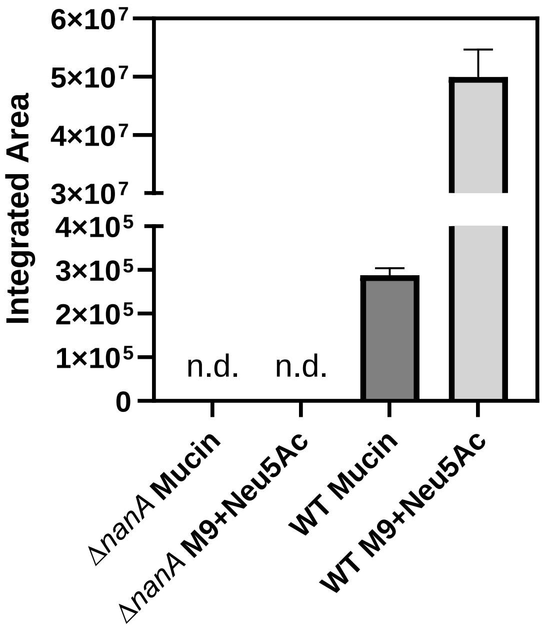

Figure S34. The *nanA* mutant cannot produce pyrazinone **3** regardless of Neu5Ac supplementation. Additional peaks likely result from isomeric (pro)pyrazinones which are produced as intermediates (scheme shown).

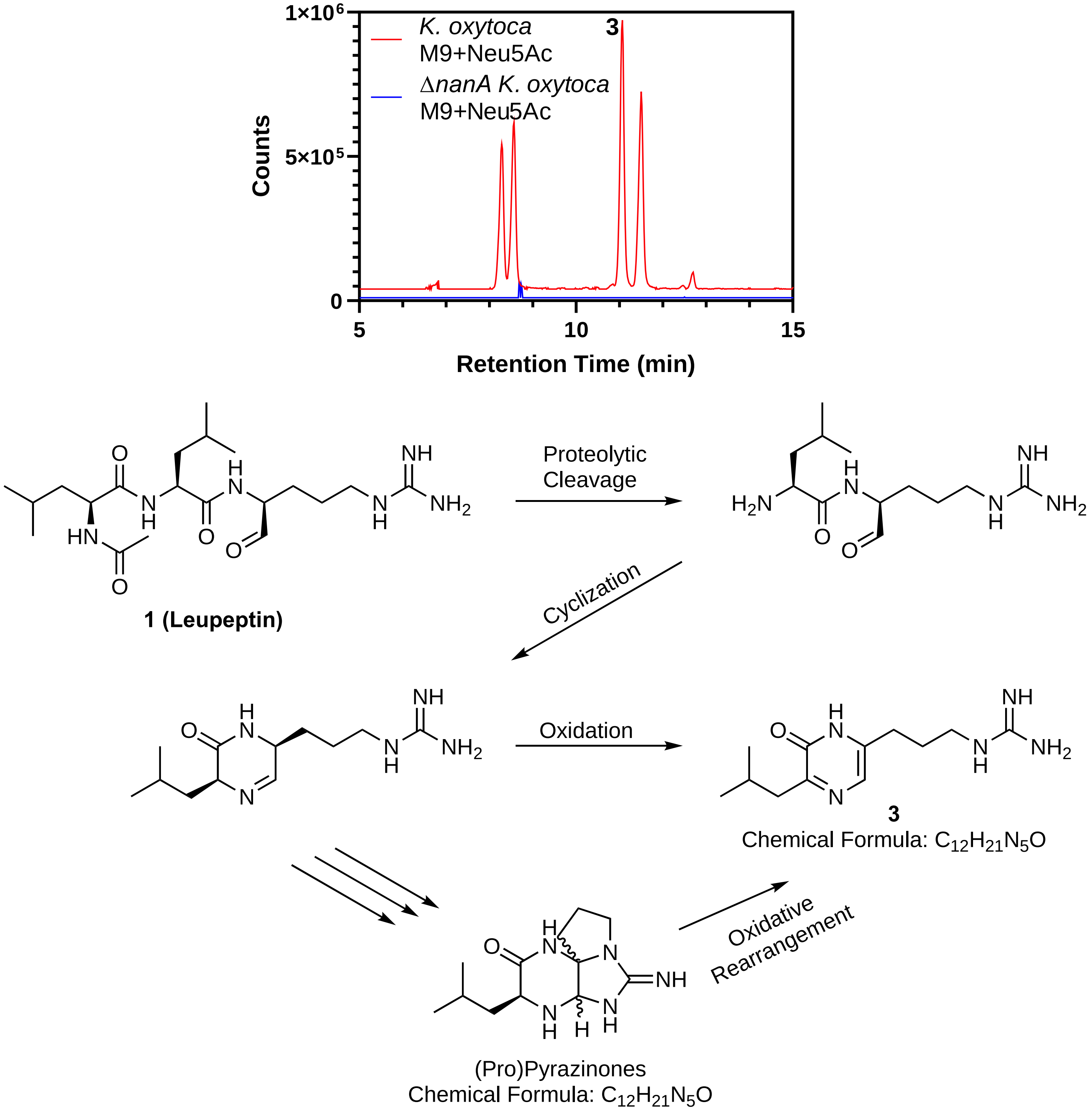

Figure S35. ESI^{+}-HRMS analysis of compounds **10a**, **10b**, **10-Fe**, and **11** between wildtype and Δ*yer K. oxytoca* strains.

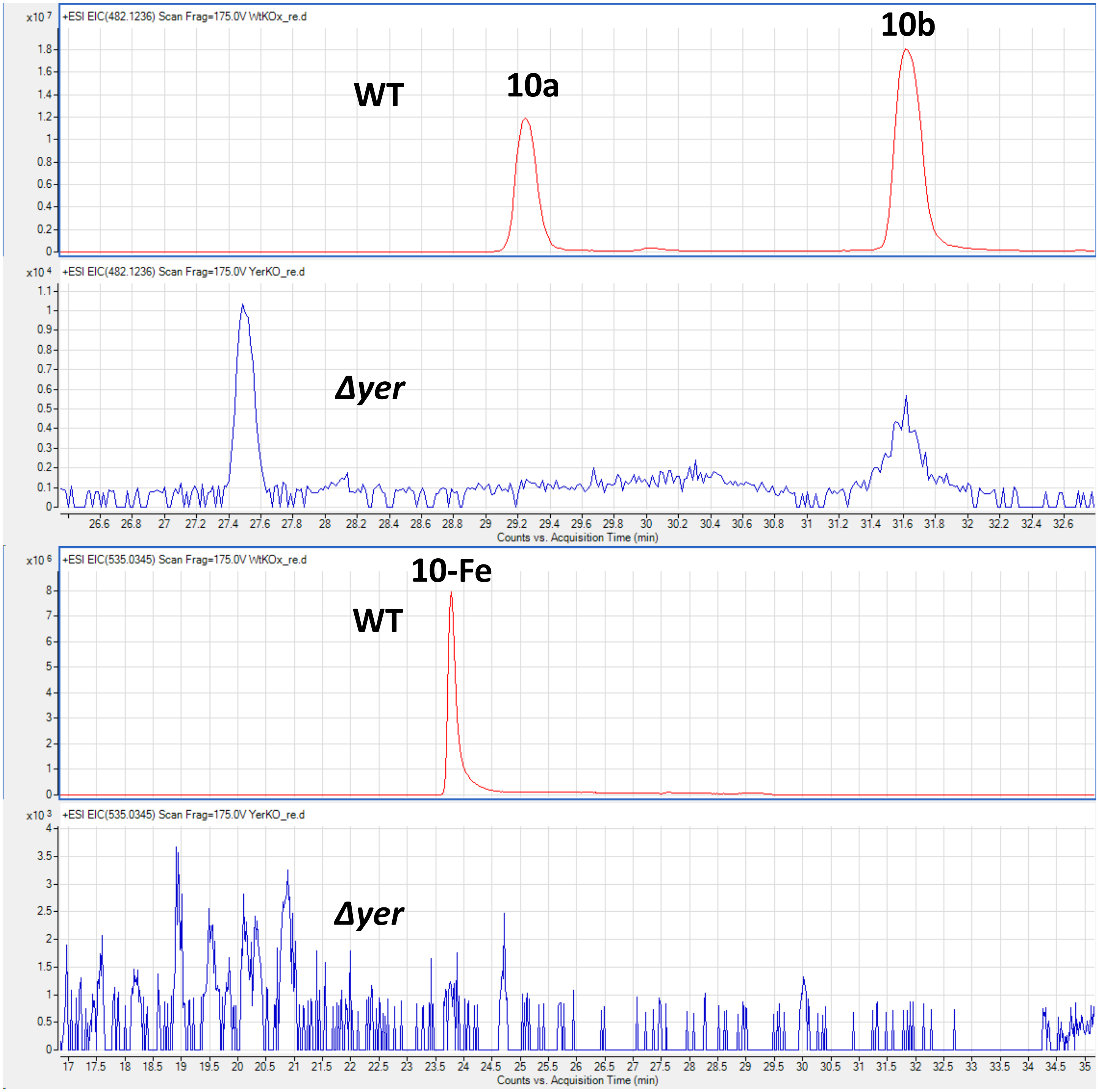

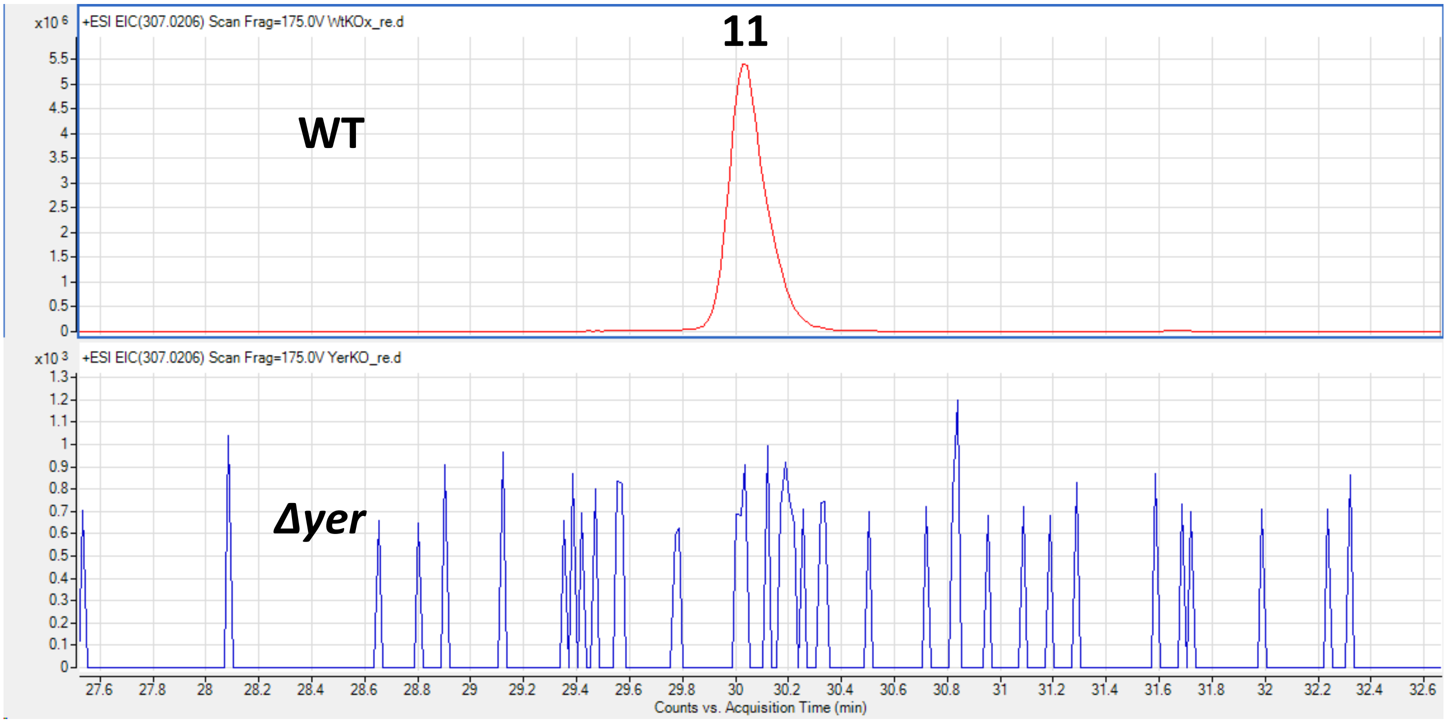

11
WT
x10 6
+ESI EIC(307.0206) Scan Frag=175.0V WtKOx_re.d
x10 3
+ESI EIC(307.0206) Scan Frag=175.0V YerKO_re.d
Δyer
Counts vs. Acquisition Time (min)

Figure S36. UV-Vis spectra of compounds **5**, **6**, **7**, **8**, and **9**.

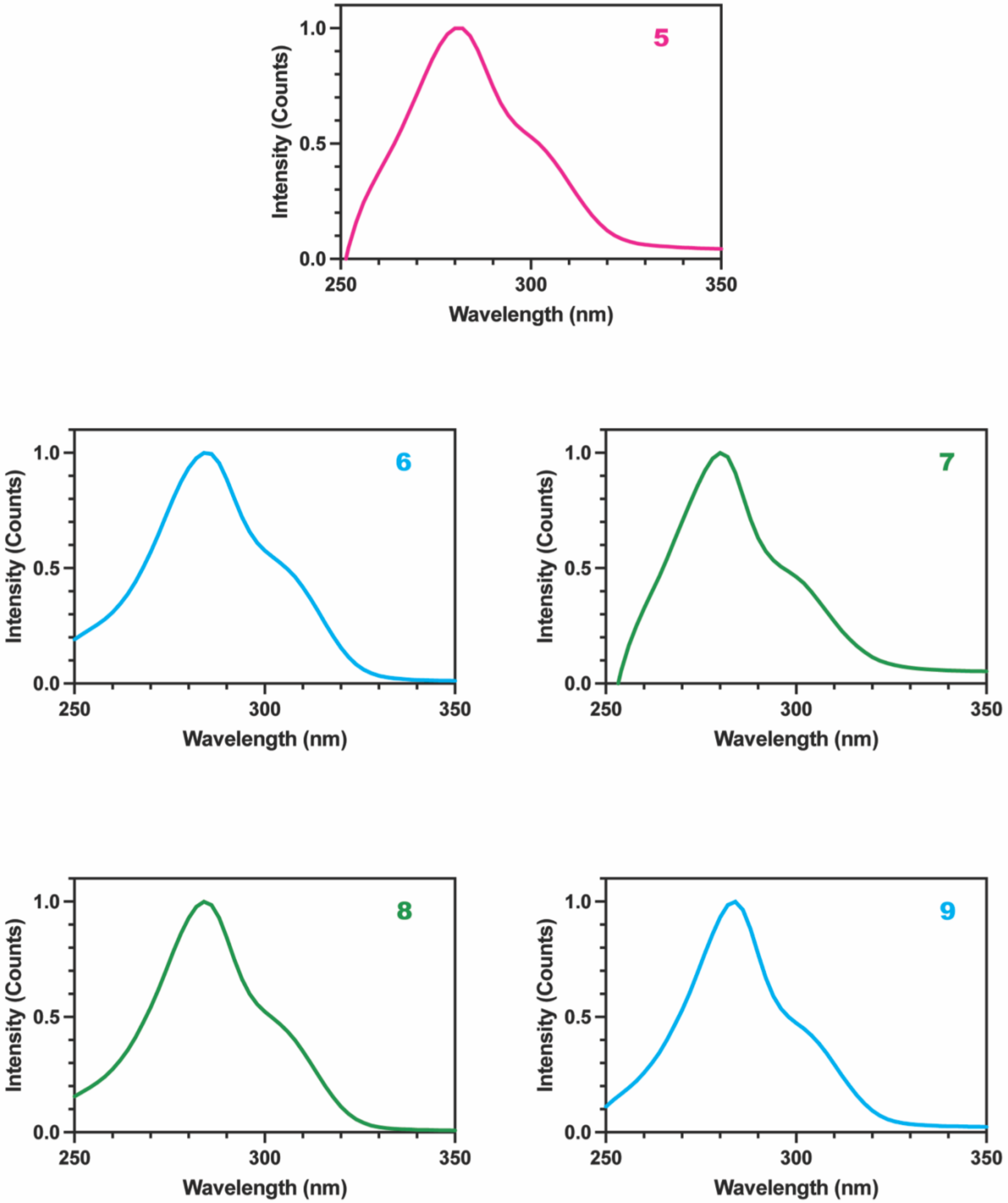

Figure S37. ESI^{+}-HRMS *m/z* envelope of **5**.

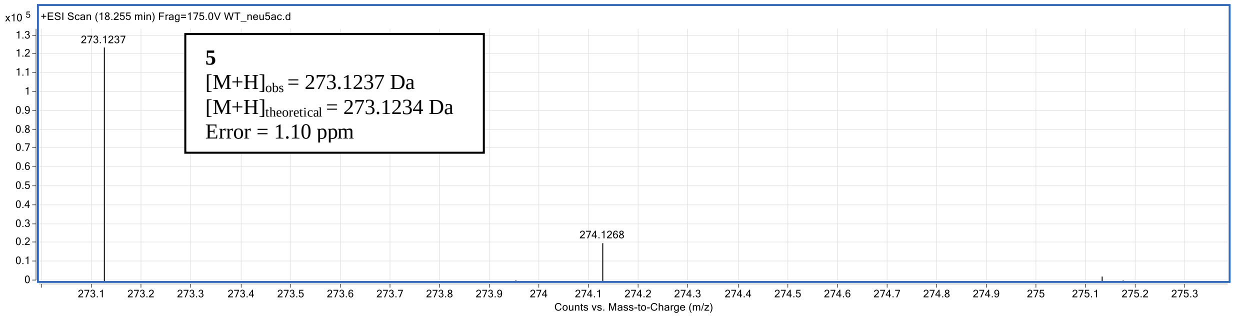

Figure S38. ESI^{+}-HRMS/MS fragmentation spectrum of **5** (20 CE).

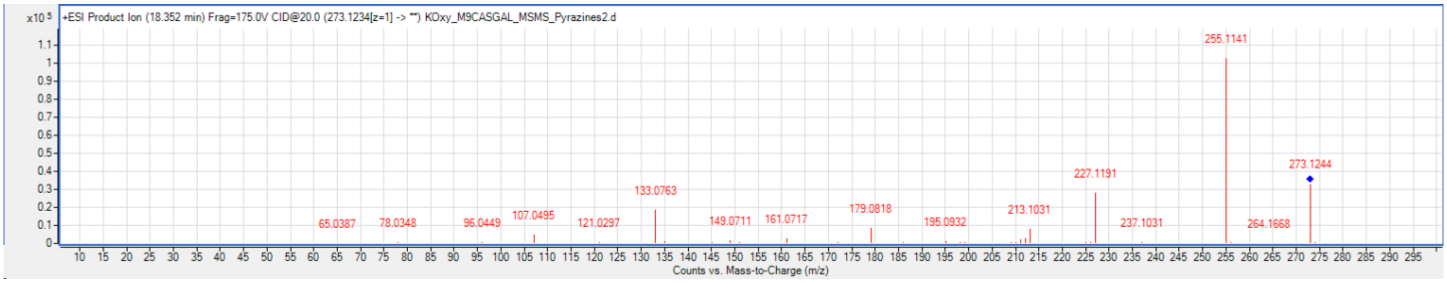

Figure S39. ESI^{+}-HRMS *m/z* envelope of **6**.

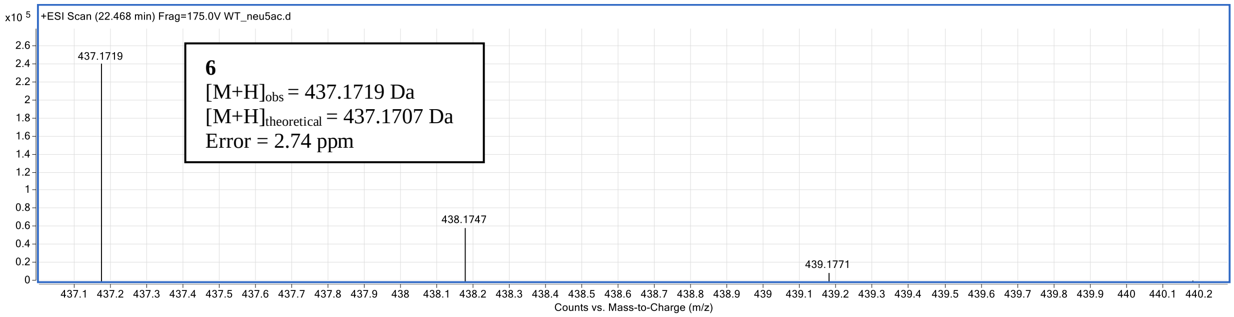

Figure S40. ESI^{+}-HRMS/MS fragmentation spectrum of **6** (20 CE).

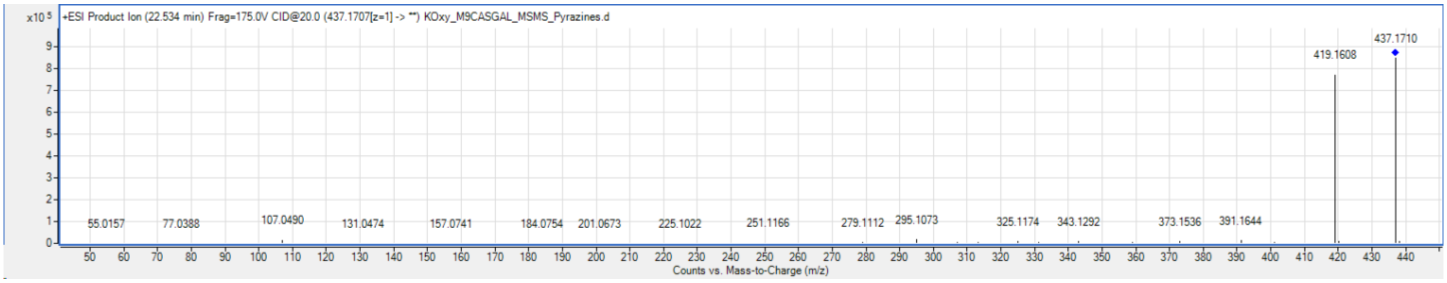

Figure S41. ESI$^+$-HRMS *m/z* envelope of **7**.

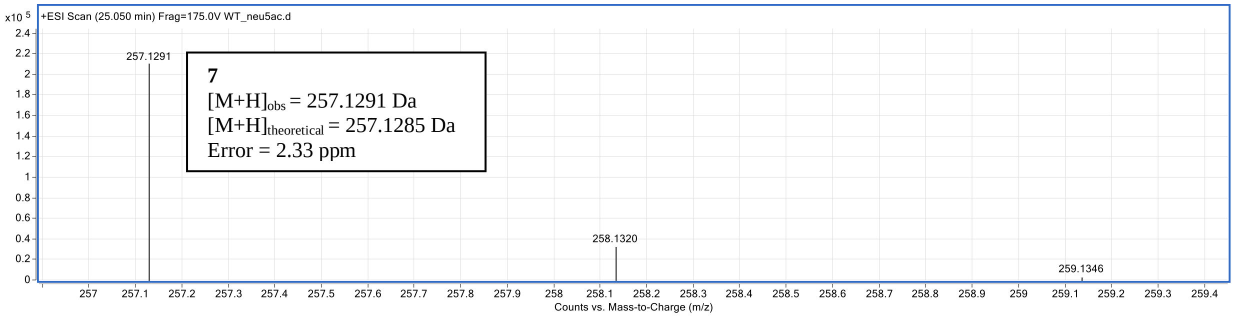

Figure S42. ESI$^+$-HRMS/MS fragmentation spectrum of **7** (20 CE).

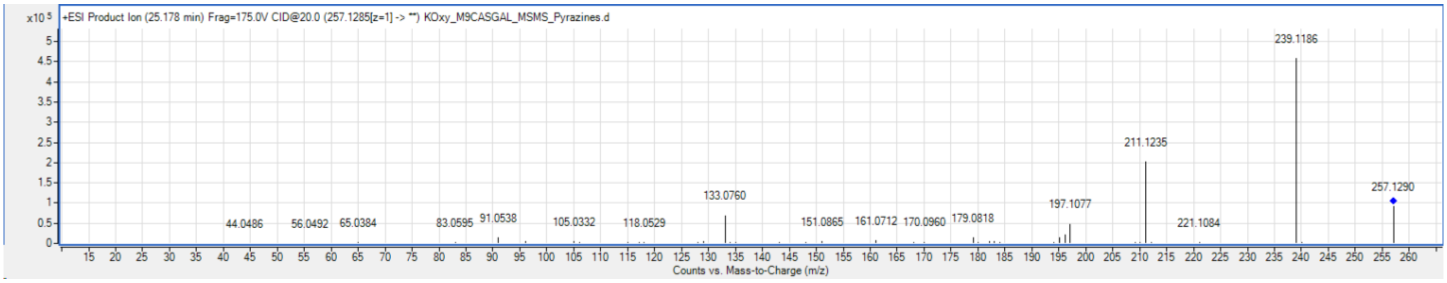

Figure S43. ESI$^+$-HRMS *m/z* envelope of **8**.

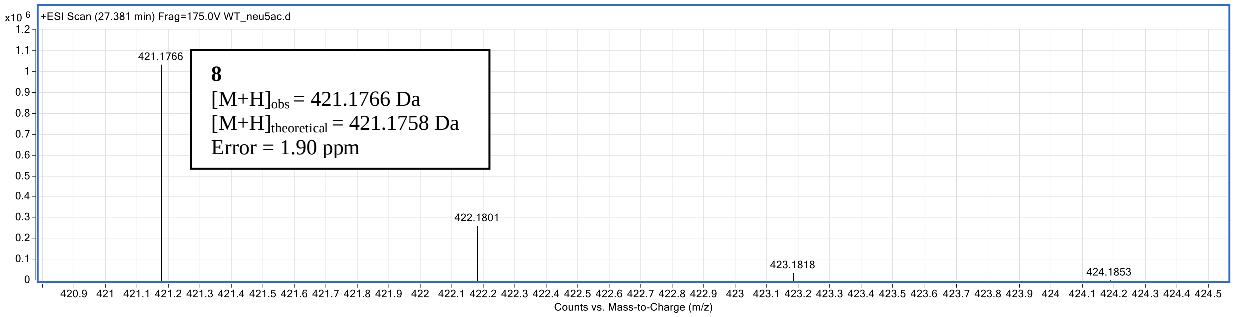

Figure S44. ESI$^+$-HRMS/MS fragmentation spectrum of **8** (20 CE).

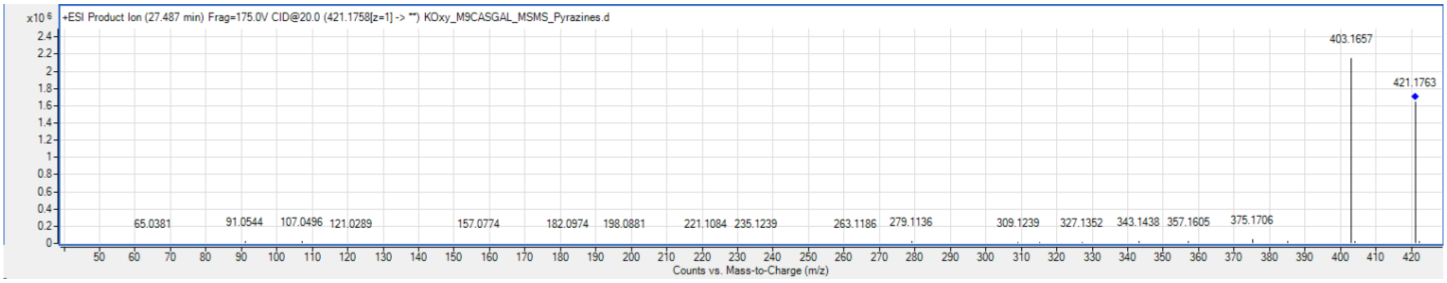

Figure S45. ESI$^+$-HRMS *m/z* envelope of **9**.

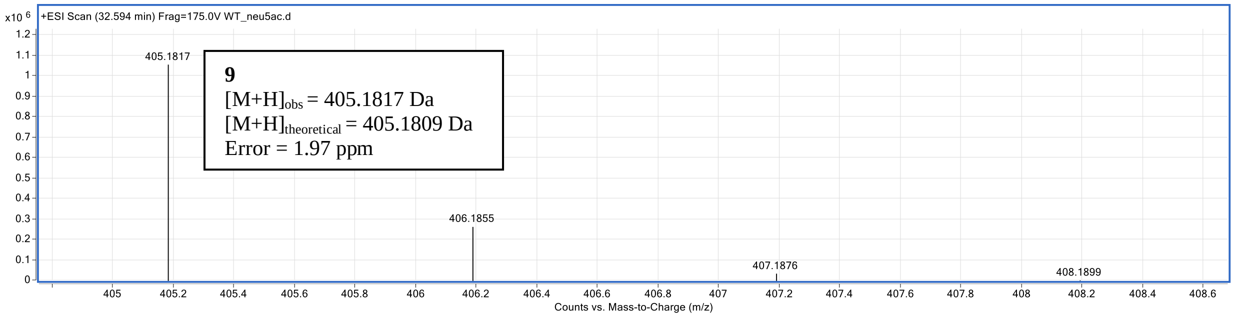

Figure S46. ESI$^+$-HRMS/MS fragmentation spectrum of **9** (20 CE).

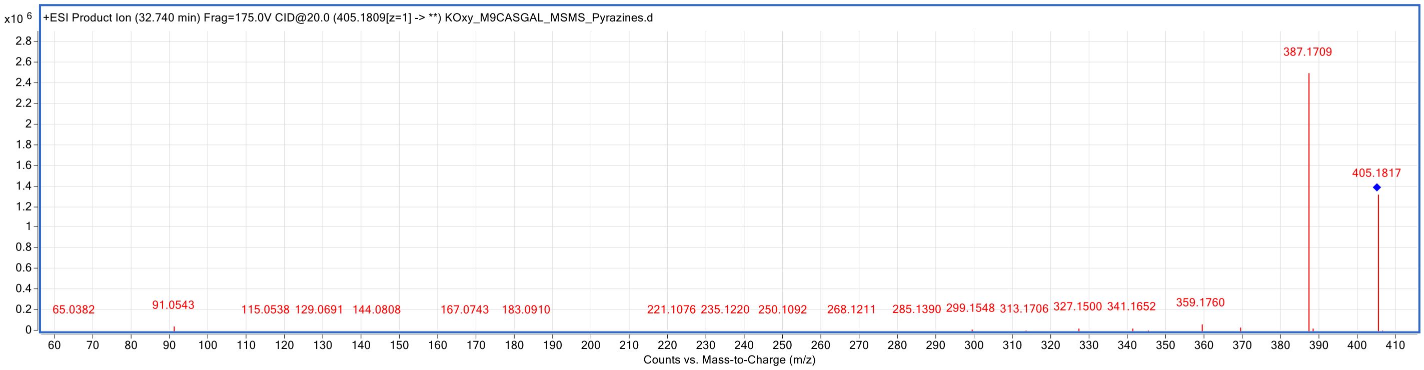

Figure S47. ESI$^+$-HRMS *m/z* envelope of **10**.

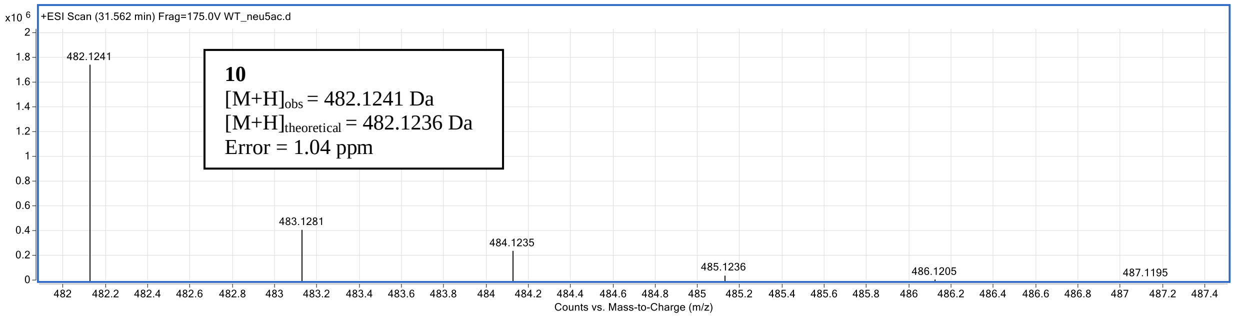

Figure S48. ESI$^+$-HRMS *m/z* envelope of **11**.

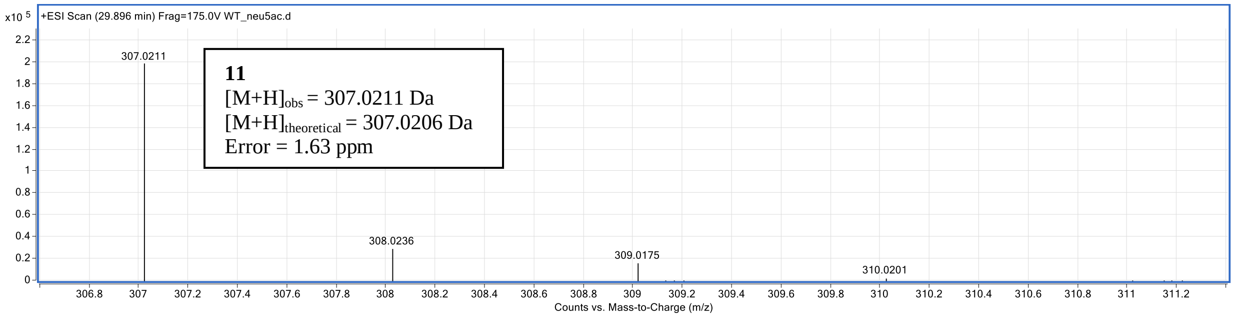

Figure S49. ESI$^+$-HRMS *m/z* envelope of **12**.

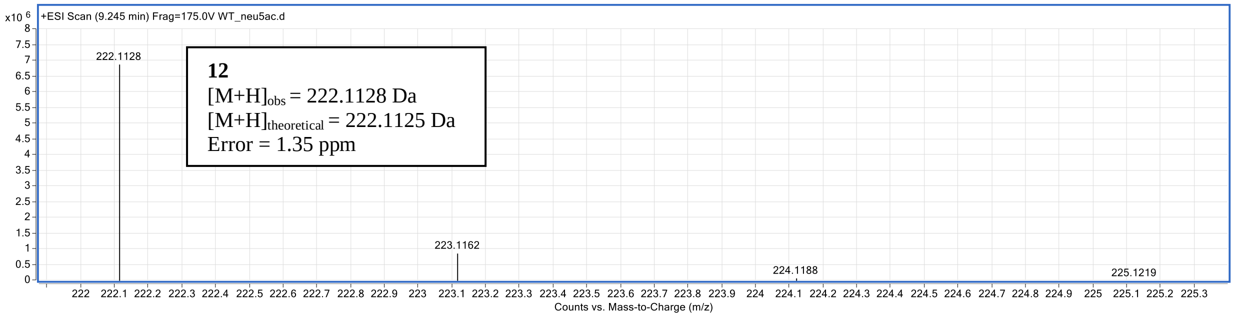

Figure S50. ESI$^+$-HRMS/MS fragmentation spectrum of **12** (20 CE).

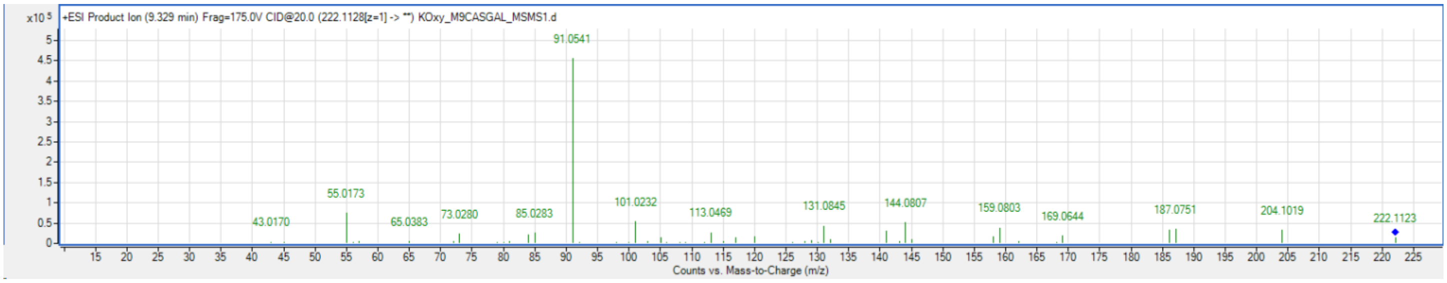

Figure S51. ESI$^+$-HRMS *m/z* envelope of **13**.

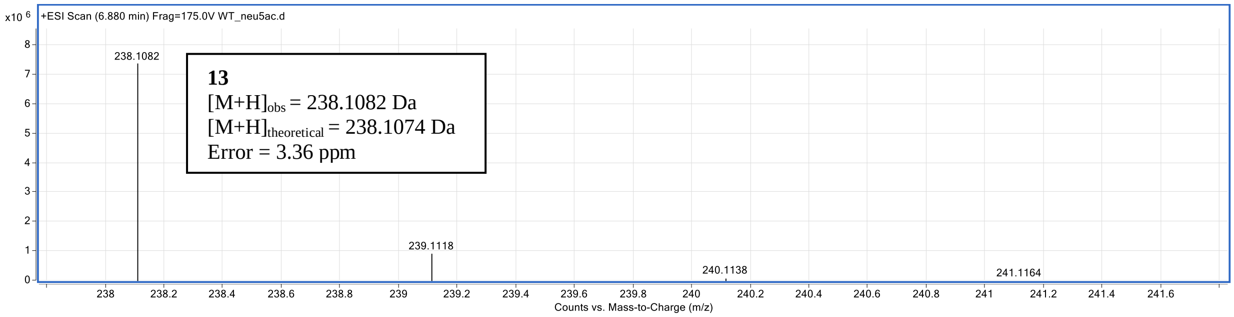

Figure S52. ESI$^+$-HRMS/MS fragmentation spectrum of **13** (20 CE).

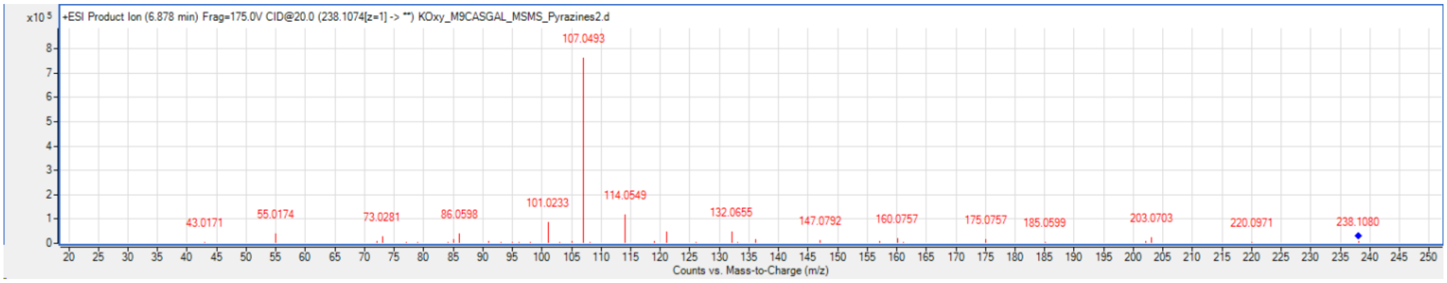

Figure S53. ESI$^+$-HRMS/MS fragmentation of *m/z* 329.1500 from **Fig. 2b** (20 CE).

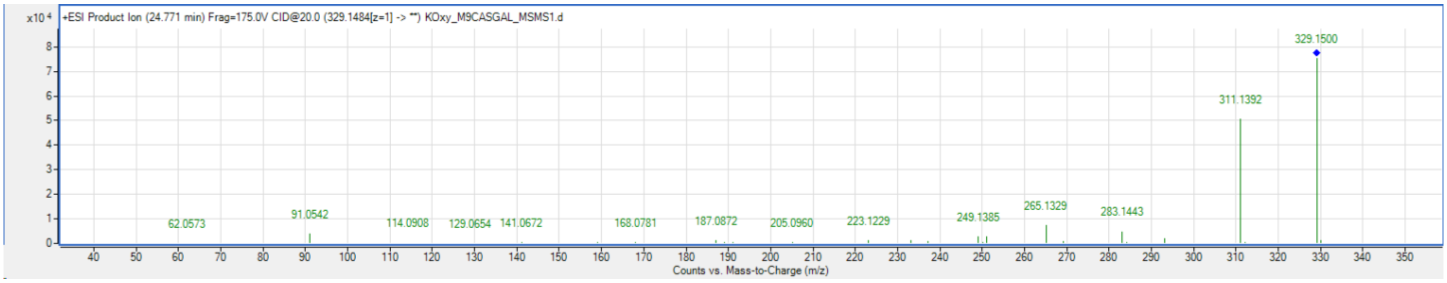

Figure S54. ESI$^+$-HRMS/MS fragmentation spectrum of *m/z* 439.1773 from **Fig. 2b** (20 CE).

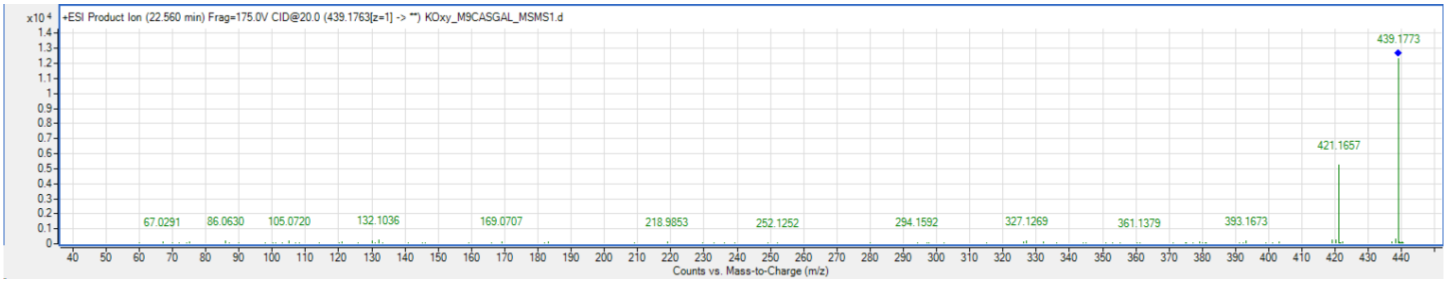

Figure S55. ESI$^+$-HRMS/MS fragmentation spectrum of *m/z* 345.1447 from **Fig. 2b** (20 CE).

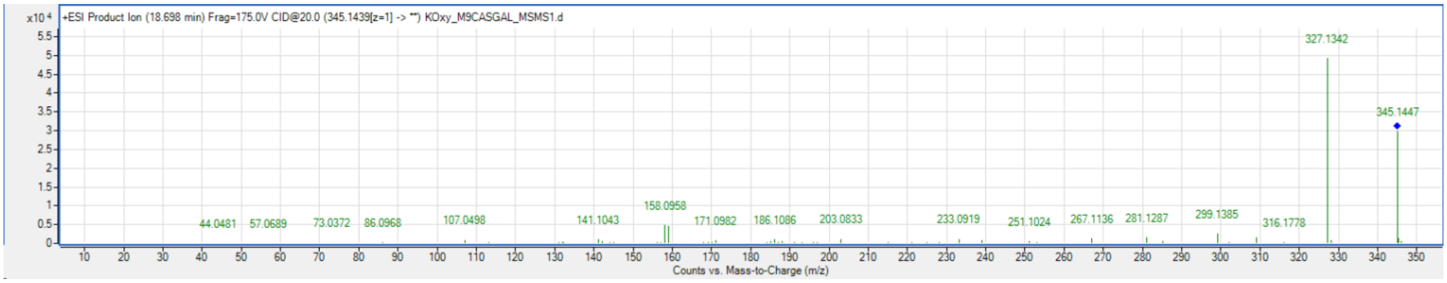

Figure S56. ESI$^+$-HRMS/MS fragmentation spectrum of *m/z* 329.1500 from **Fig. 2b** (20 CE).

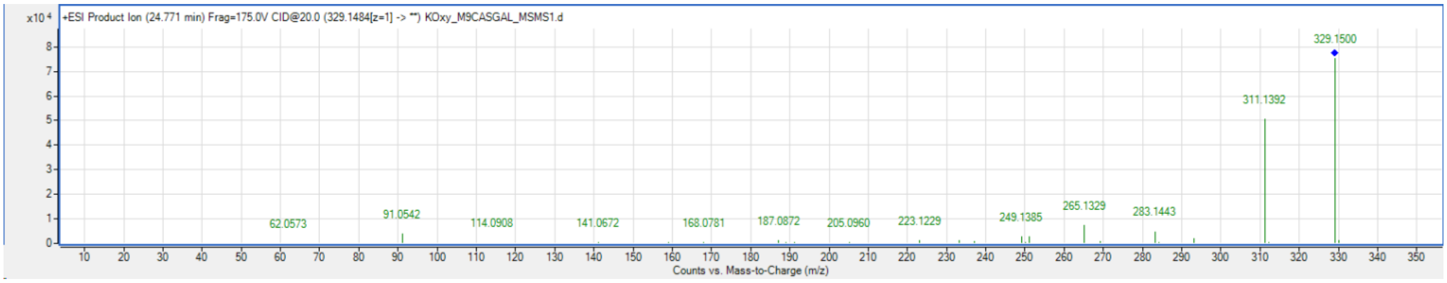

Figure S57. ESI$^+$-HRMS/MS fragmentation spectrum of *m/z* 315.1339 from **Fig. 2b** (20 CE).

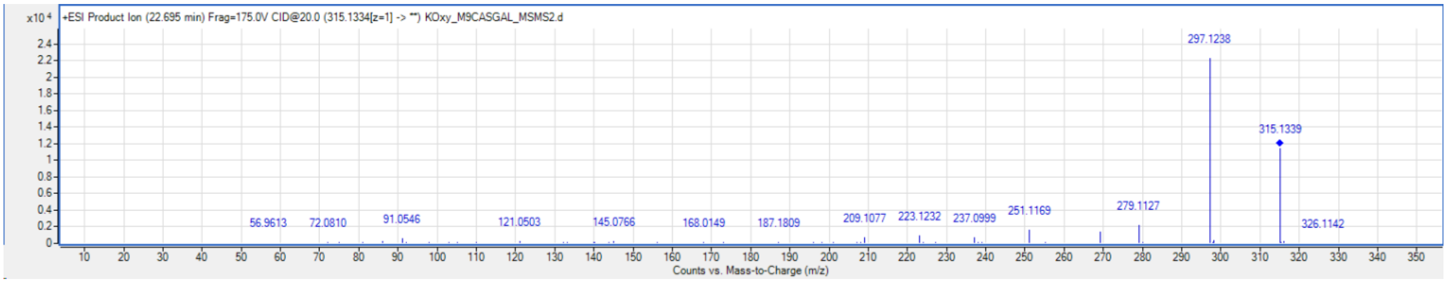

Figure S58. ESI$^+$-HRMS/MS fragmentation spectrum of *m/z* 363.1707 from **Fig. 2b** (20 CE).

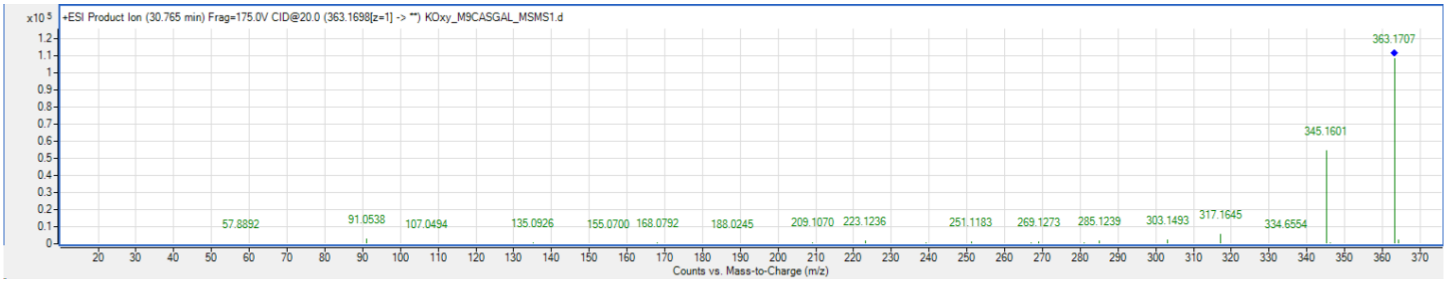

Figure S59. ESI$^+$-HRMS/MS fragmentation spectrum of *m/z* 347.1758 from **Fig. 2b** (20 CE).

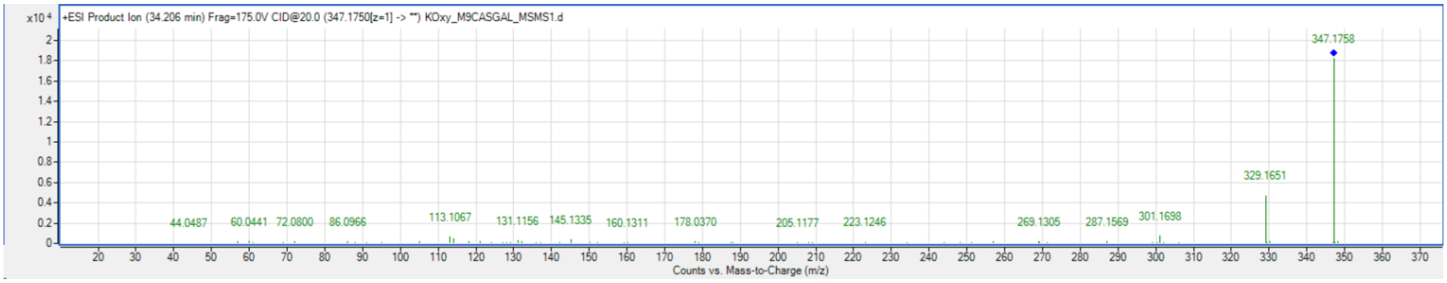

Figure S60. ESI$^+$-HRMS/MS fragmentation spectrum of *m/z* 361.1907 from **Fig. 2b** (20 CE).

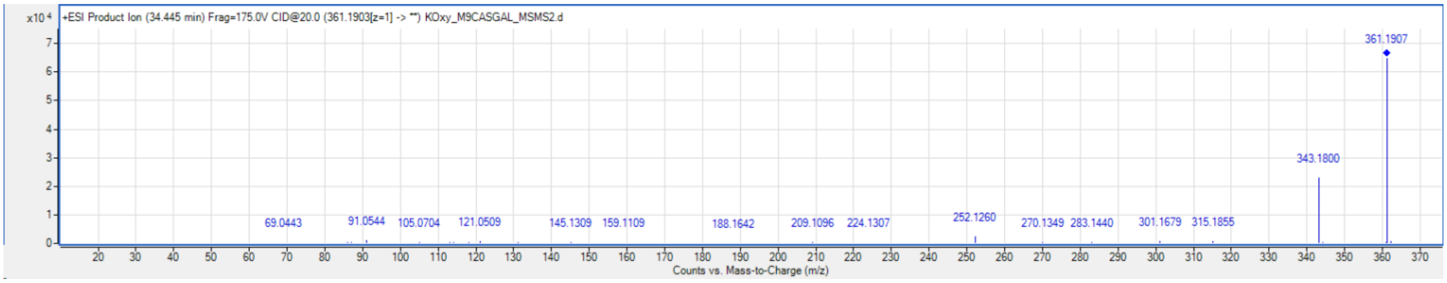

Figure S61. ESI$^+$-HRMS/MS fragmentation spectrum of *m/z* 271.1442 from **Fig. 2b** (20 CE).

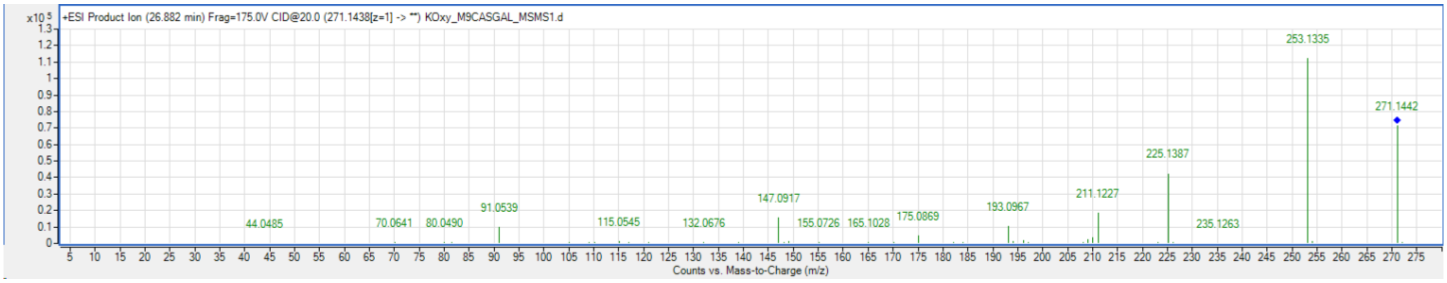

Figure S62. ESI$^+$-HRMS/MS fragmentation spectrum of *m/z* 287.1393 from **Fig. 2b** (20 CE).

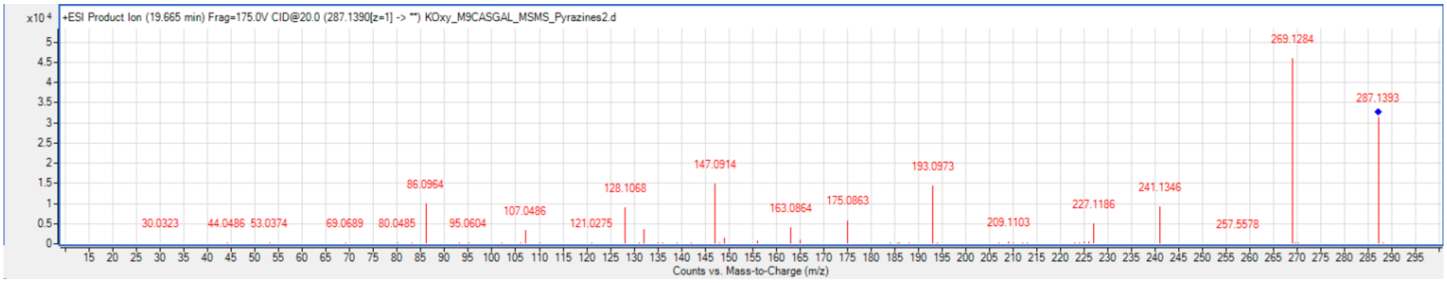

Figure S63. ESI$^+$-HRMS/MS fragmentation spectrum of *m/z* 285.1601 from **Fig. 2b** (20 CE).

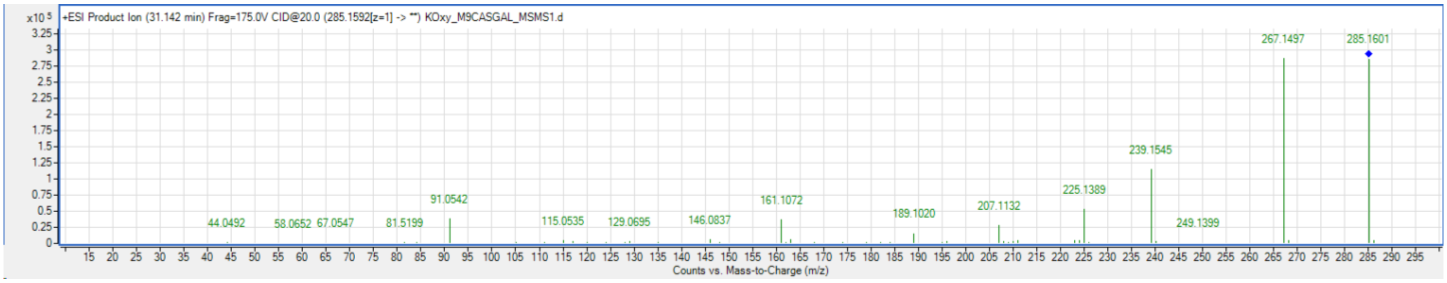

Figure S64. ESI$^+$-HRMS/MS fragmentation spectrum of *m/z* 301.1553 from **Fig. 2b** (20 CE).

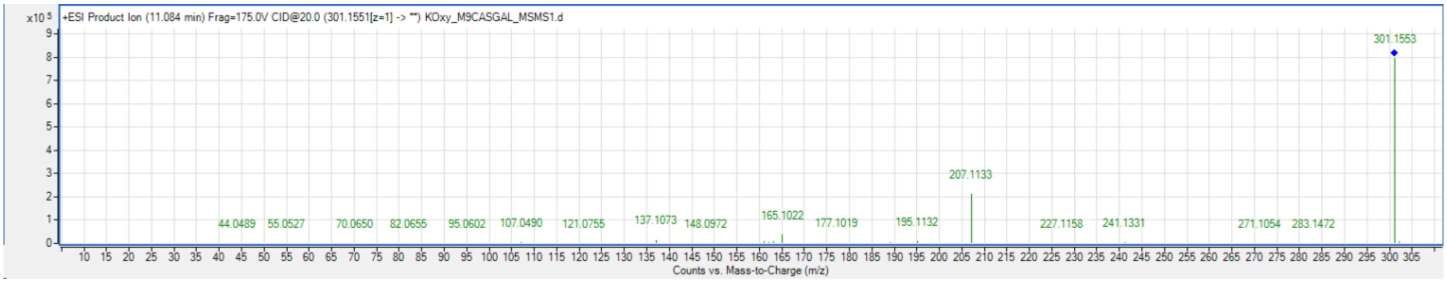

Figure S65. ESI$^+$-HRMS/MS fragmentation spectrum of *m/z* 321.1593 from **Fig. 2b** (20 CE).

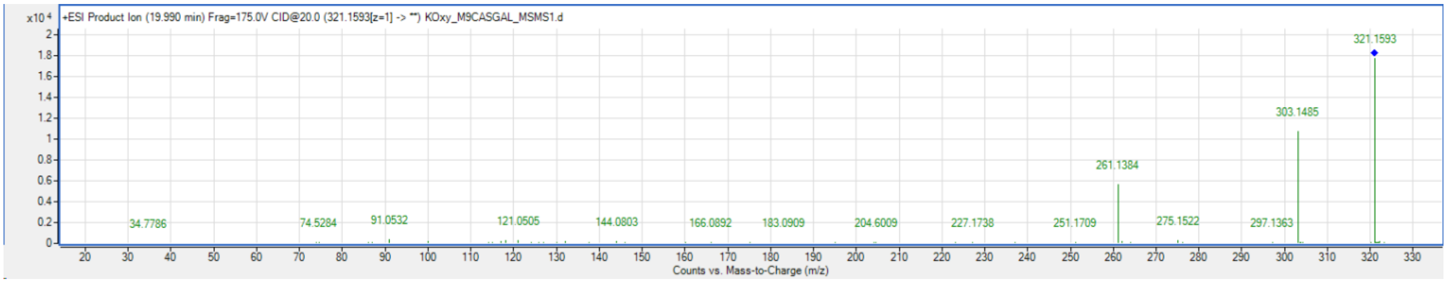

Figure S66. ESI$^+$-HRMS/MS fragmentation spectrum of *m/z* 264.1248 from **Fig. 2b** (20 CE).

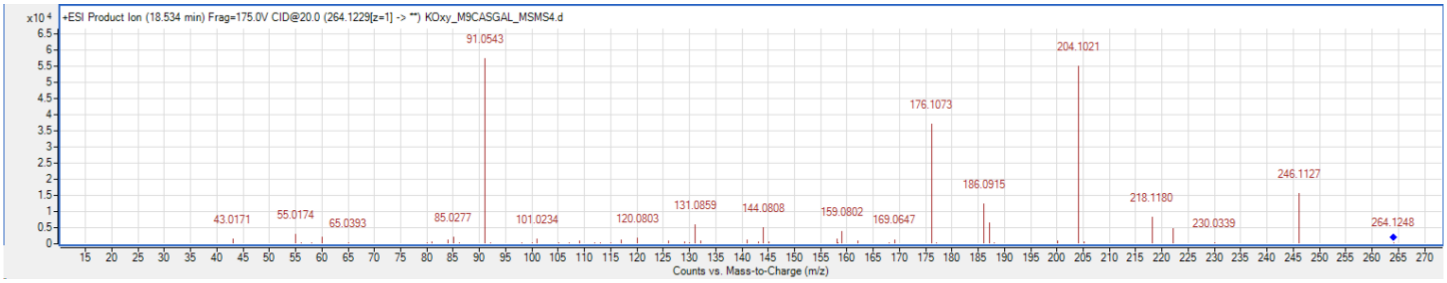

Figure S67. ESI$^+$-HRMS/MS fragmentation spectrum of *m/z* 222.0600 from **Fig. 2b** (20 CE).

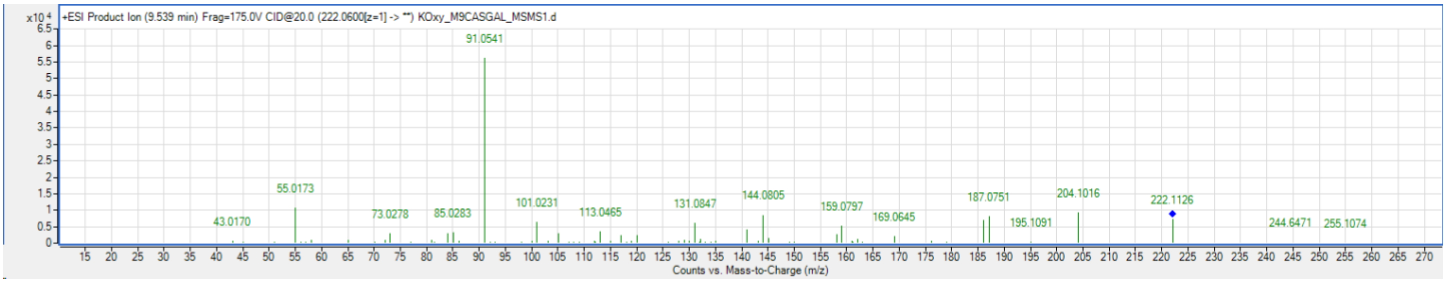

Figure S68. ESI$^+$-HRMS/MS fragmentation spectrum of *m/z* 246.0616 from **Fig. 2b** (20 CE).

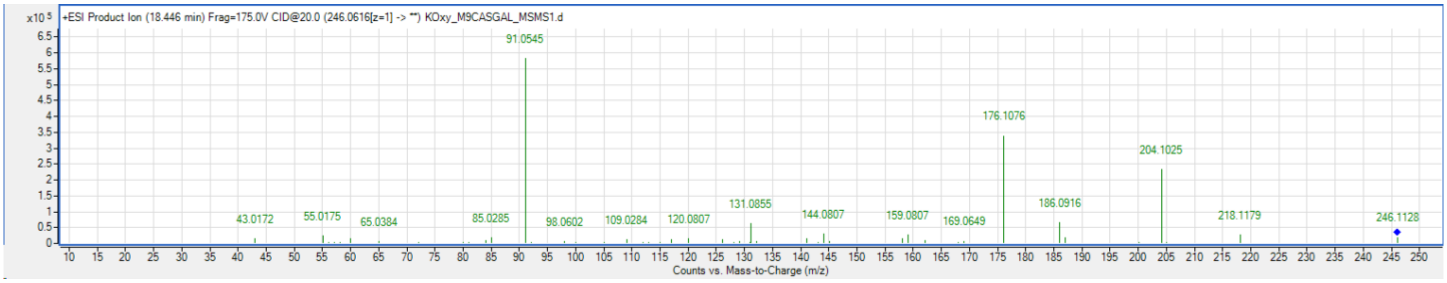

Figure S69. ESI$^+$-HRMS/MS fragmentation spectrum of *m/z* 204.0618 from **Fig. 2b** (20 CE).

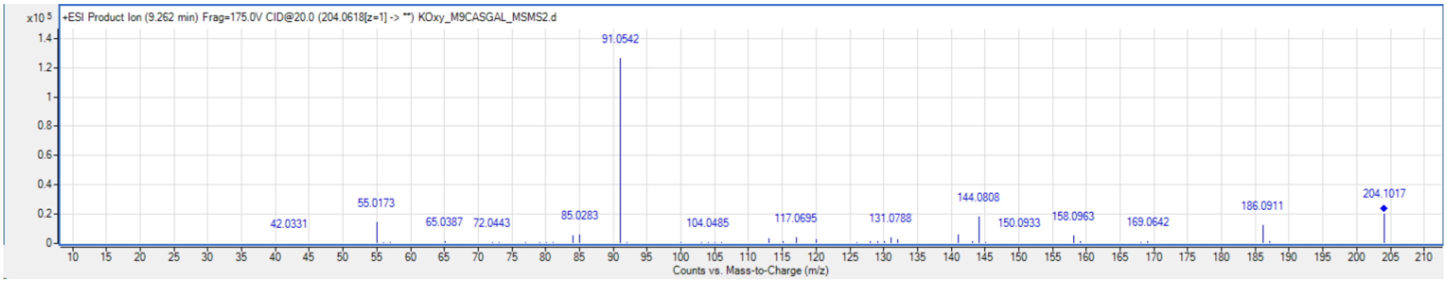

Figure S70. ESI$^+$-HRMS/MS fragmentation spectrum of *m/z* 246.1120 from **Fig. 2b** (20 CE).

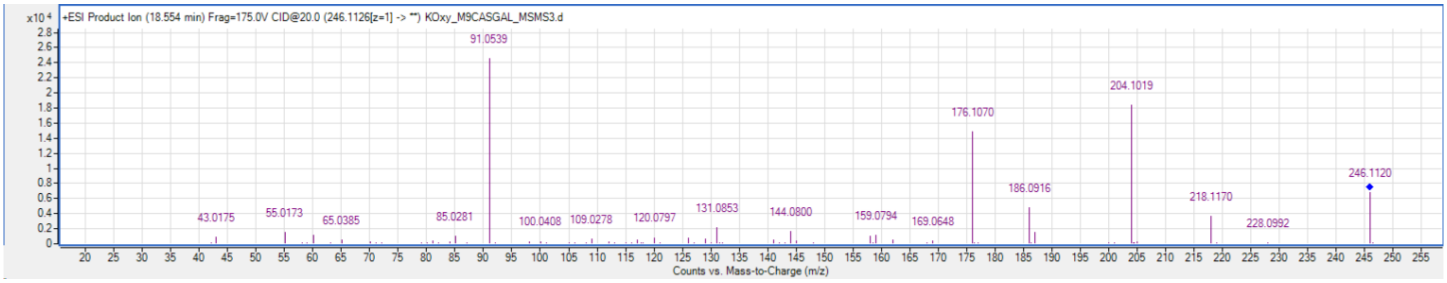

Figure S71. ESI$^+$-HRMS/MS fragmentation spectrum of **12** (10 CE) from in vitro reactions.

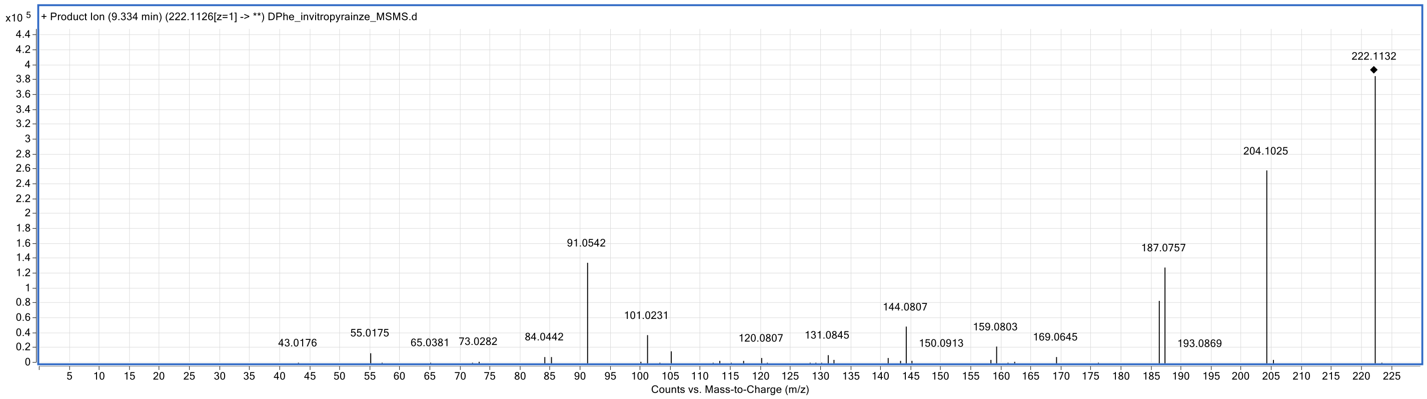

Figure S72. ESI$^+$-HRMS/MS fragmentation spectrum of **13** (10 CE) from in vitro reactions.

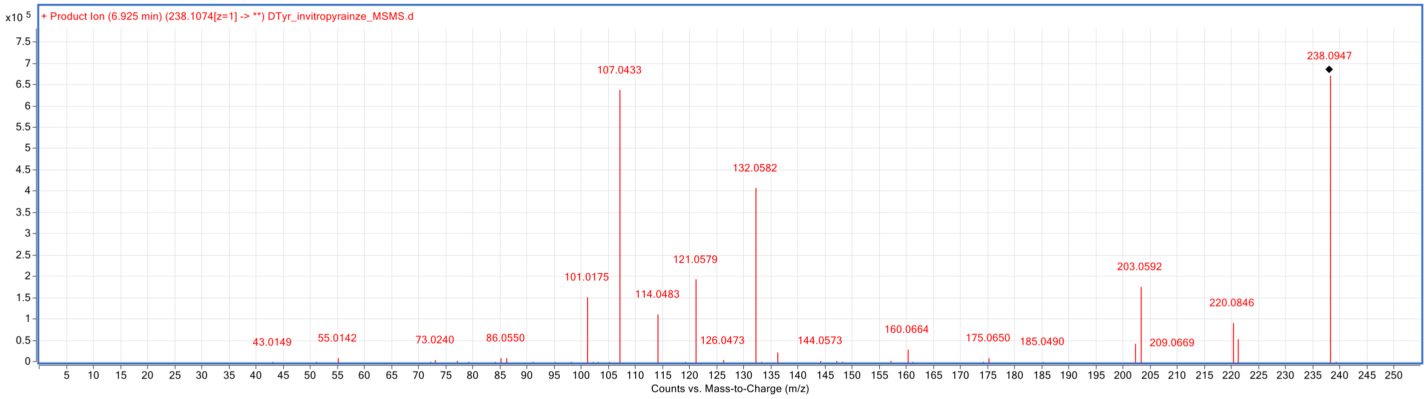

Figure S73. ESI$^+$-HRMS *m/z* envelope of **14** from in vitro reactions.

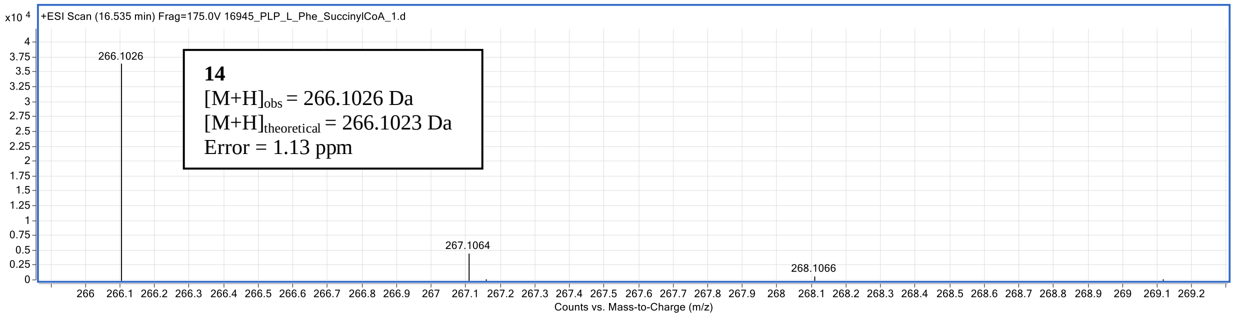

Figure S74. ESI$^+$-HRMS/MS fragmentation spectrum of **14** (10 CE) from in vitro reactions.

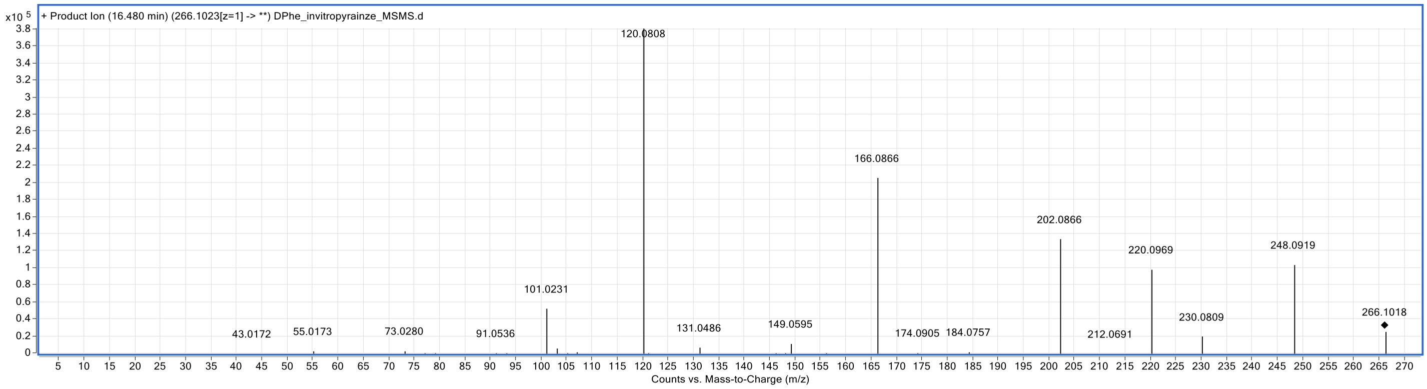

Figure S75. ESI$^+$-HRMS *m/z* envelope of **15** from in vitro reactions.

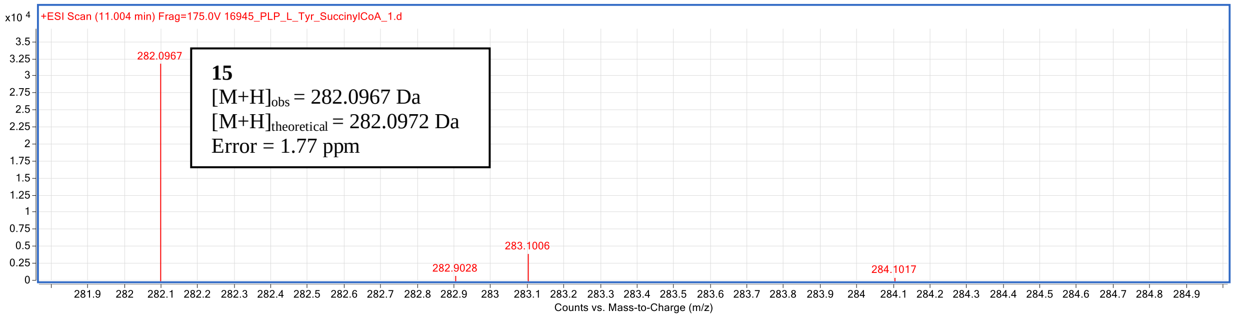

Figure S76. ESI$^+$-HRMS/MS fragmentation spectrum of **15** (10 CE) from in vitro reactions.

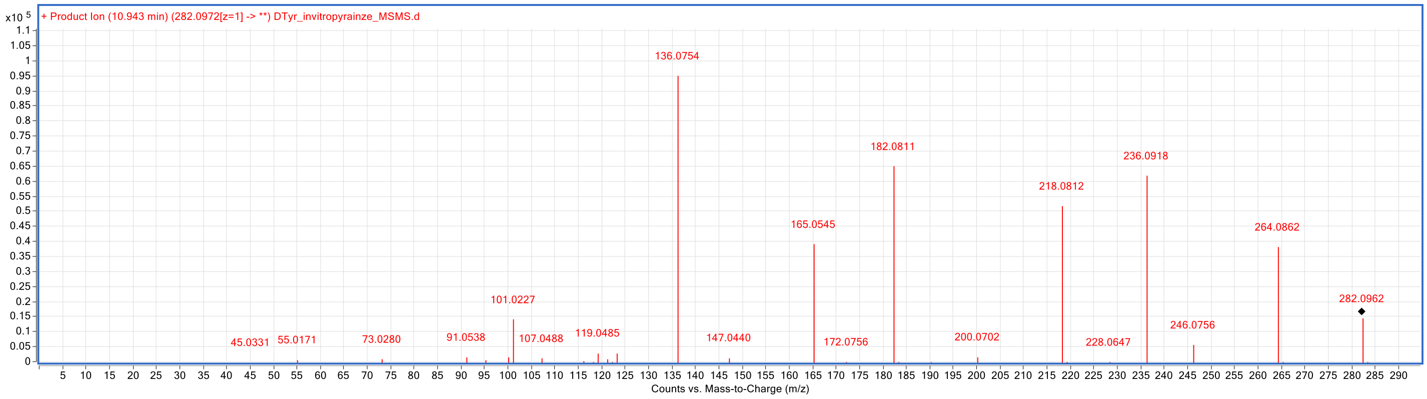

Figure S77. ESI$^+$-HRMS *m/z* envelope of **16** from in vitro reactions.

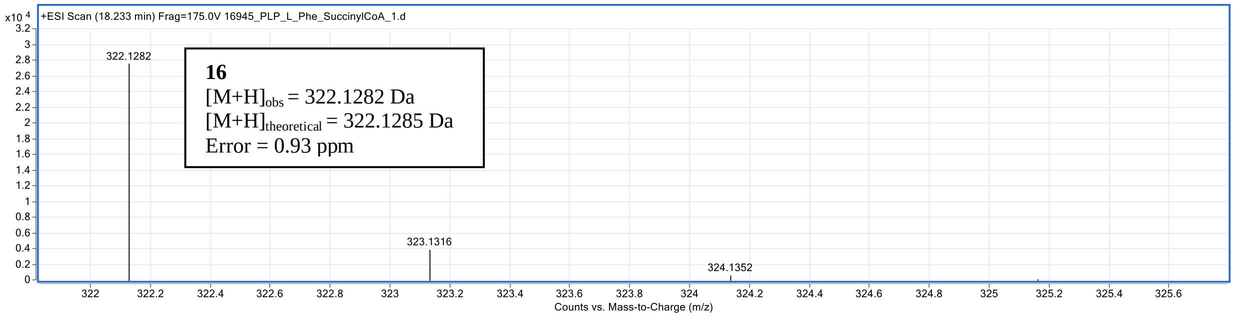

Figure S78. ESI$^+$-HRMS/MS fragmentation spectrum of **16** (10 CE) from in vitro reactions.

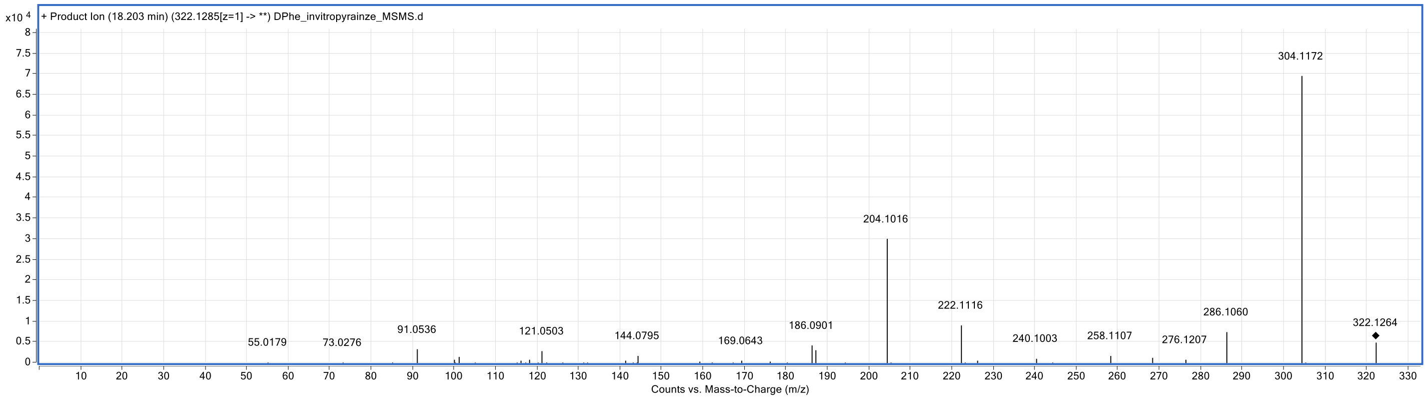

Figure S79. ESI$^+$-HRMS *m/z* envelope of **17** from in vitro reactions.

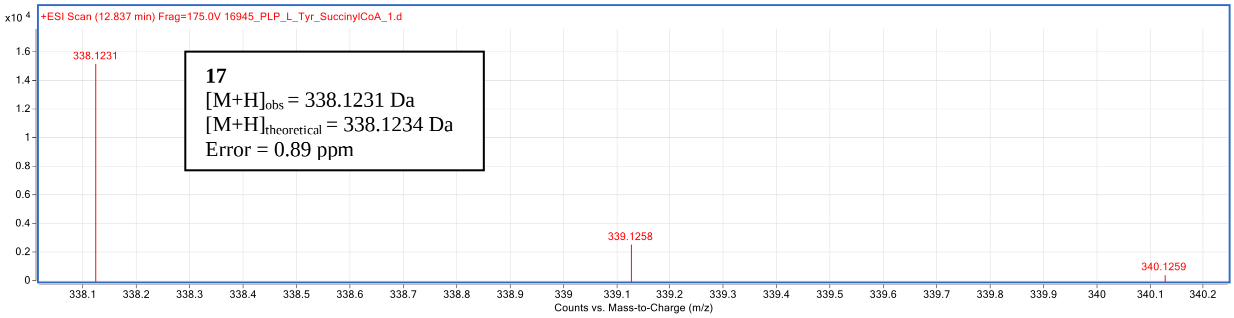

Figure S80. ESI$^+$-HRMS/MS fragmentation spectrum of **17** (10 CE) from in vitro reactions.

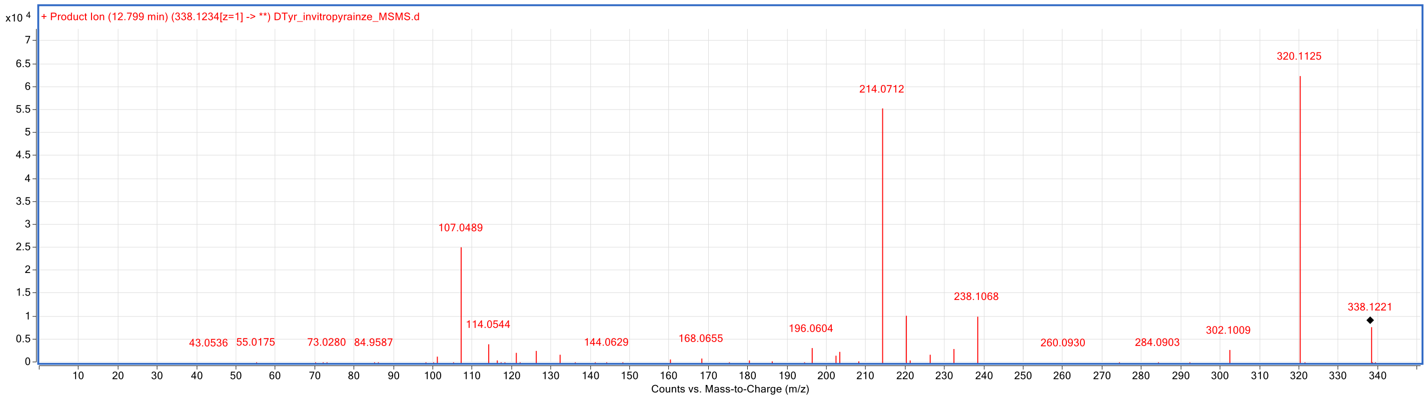

Figure S81. ^{1}H NMR spectrum of **6** (600 MHz, methanol-d_4).

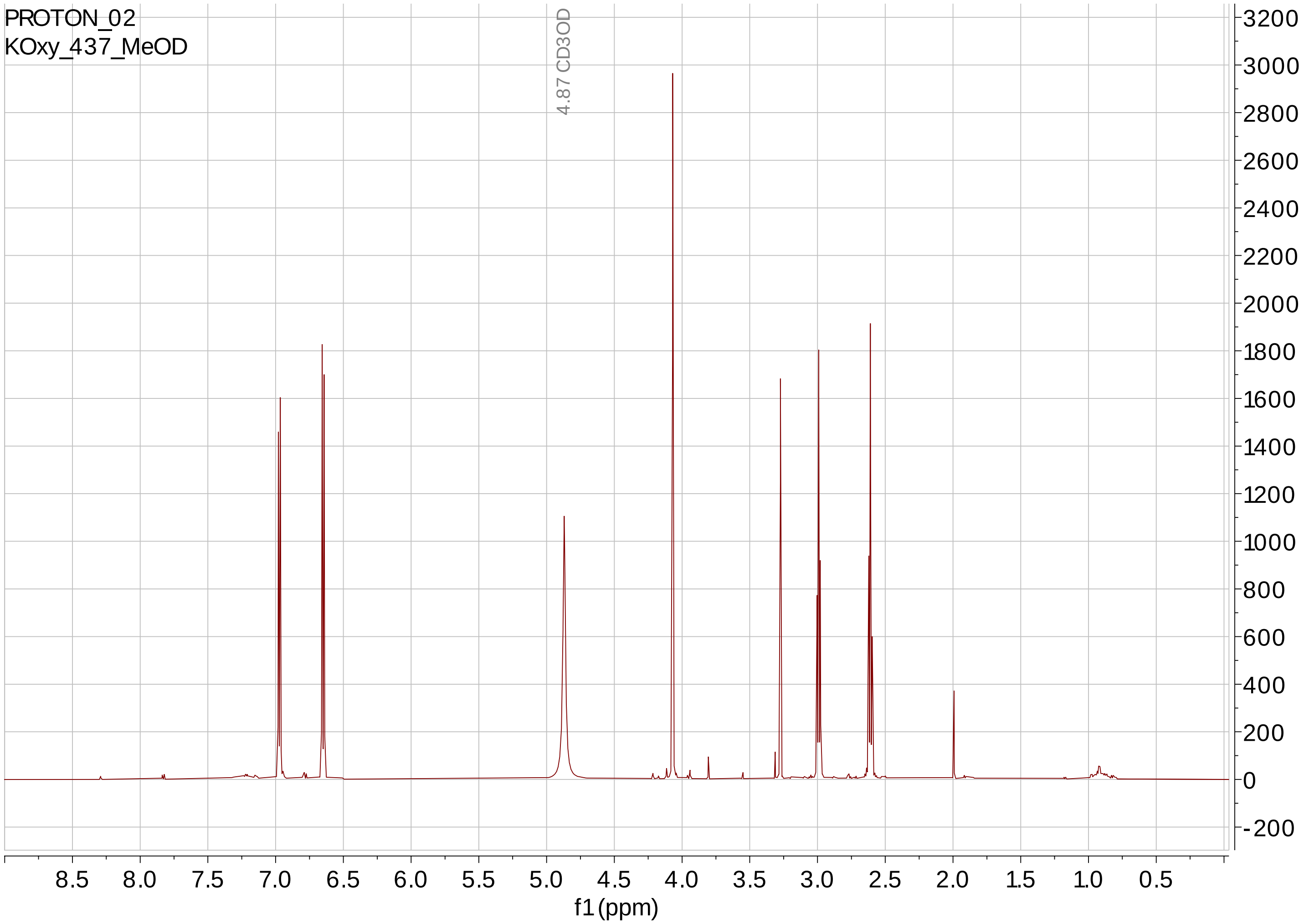

Figure S82. gCOSY spectrum of **6** (600 MHz, methanol-d_4).

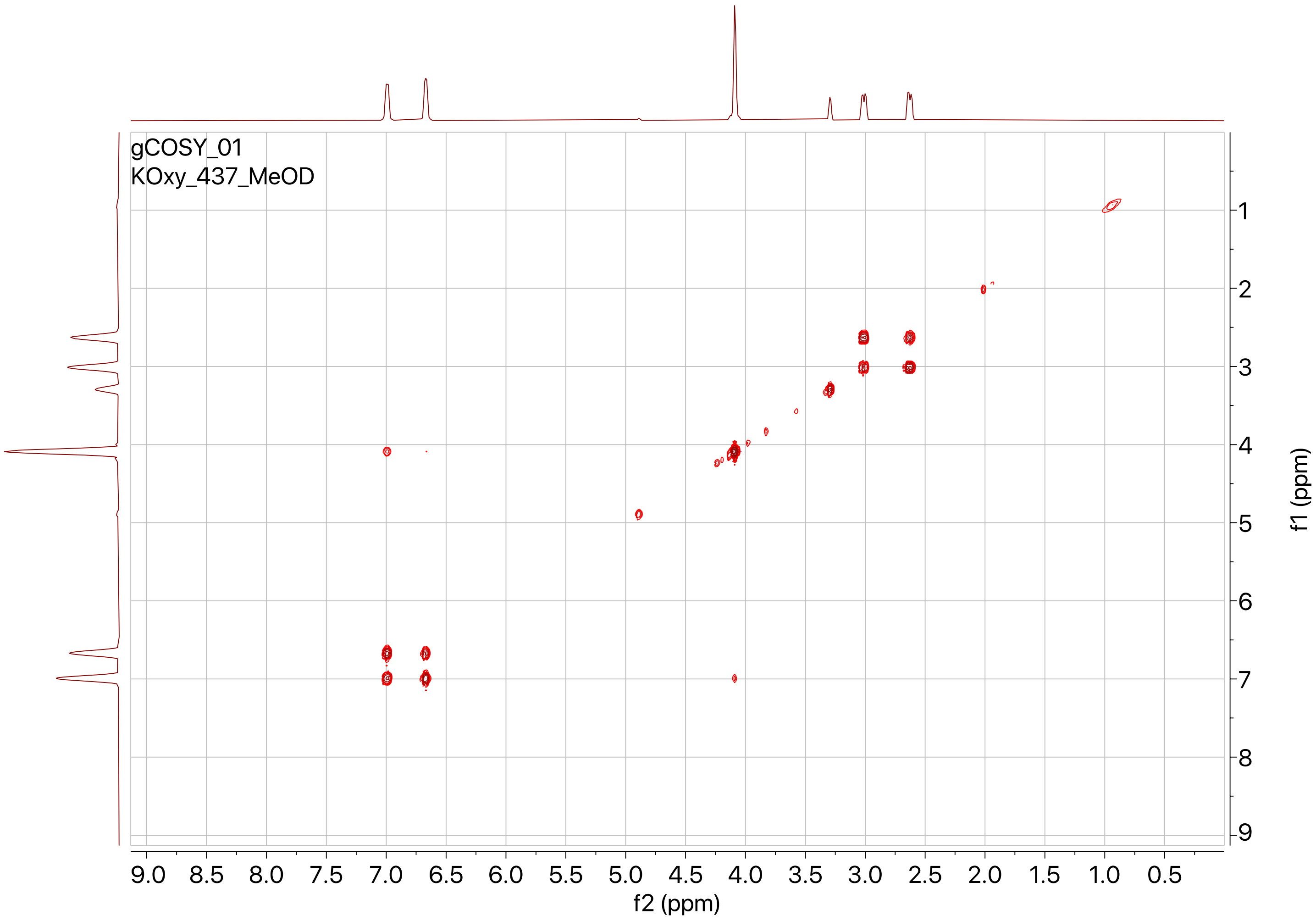

Figure S83. gHSQC spectrum of **6** (600 MHz, methanol-d_4).

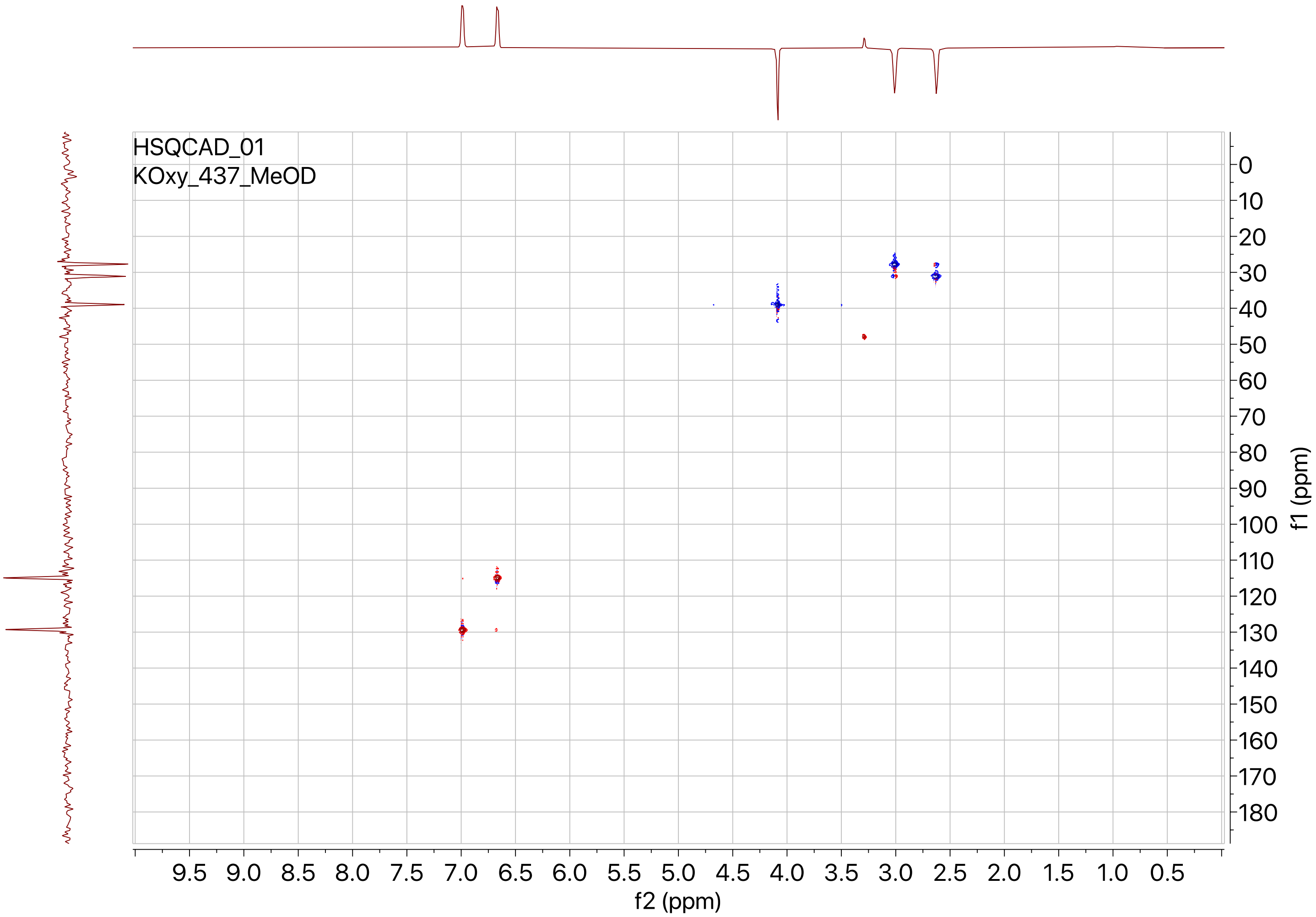

Figure S84. gHMBC spectrum of **6** (600 MHz, methanol-d_4).

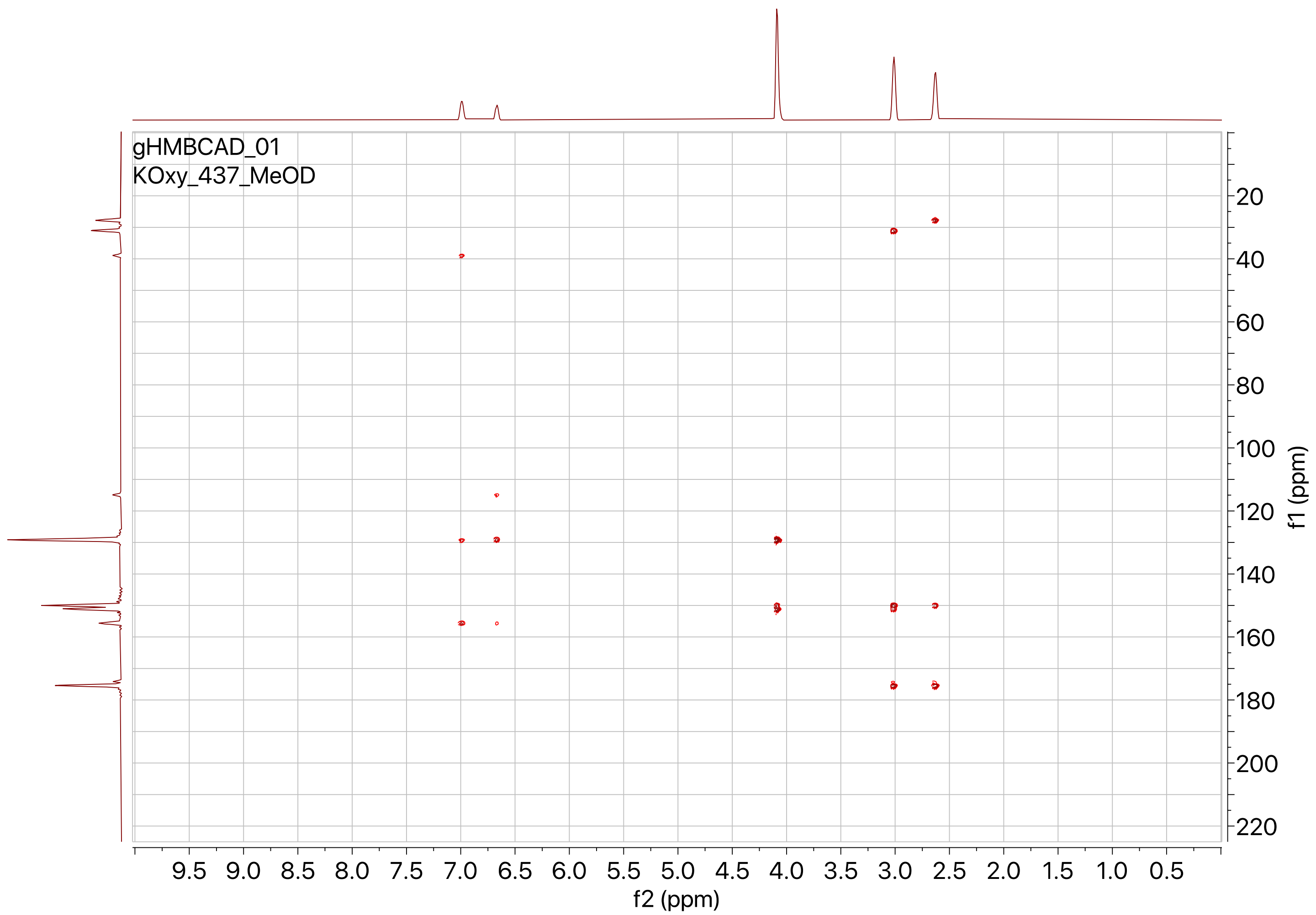

Figure S85. ^{1}H, ^{15}N-gHMBC spectrum of **6** (600 MHz, methanol-d_4).

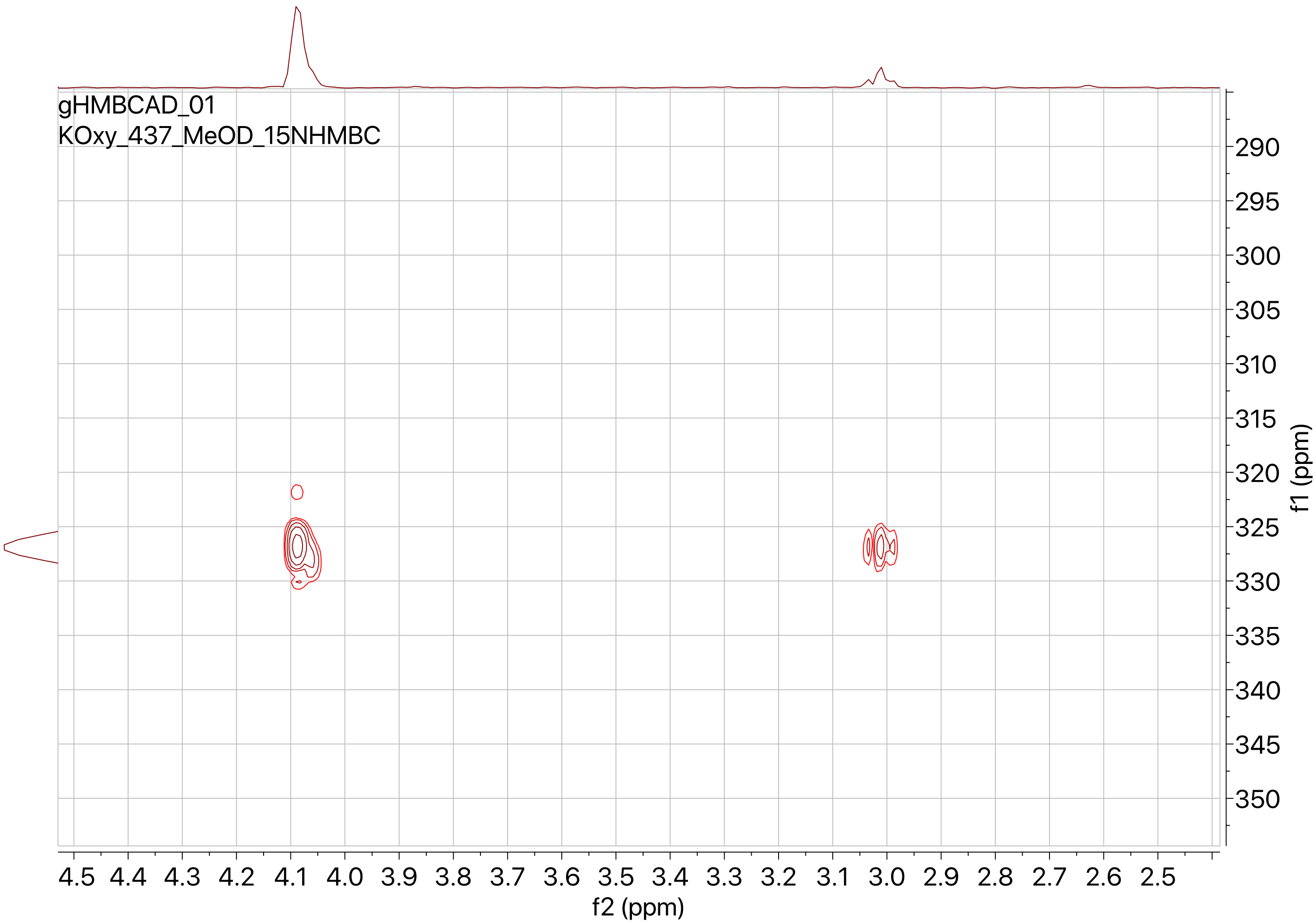

Figure S86. ^{1}H NMR spectrum of **7** (600 MHz, acetonitrile-d_3).

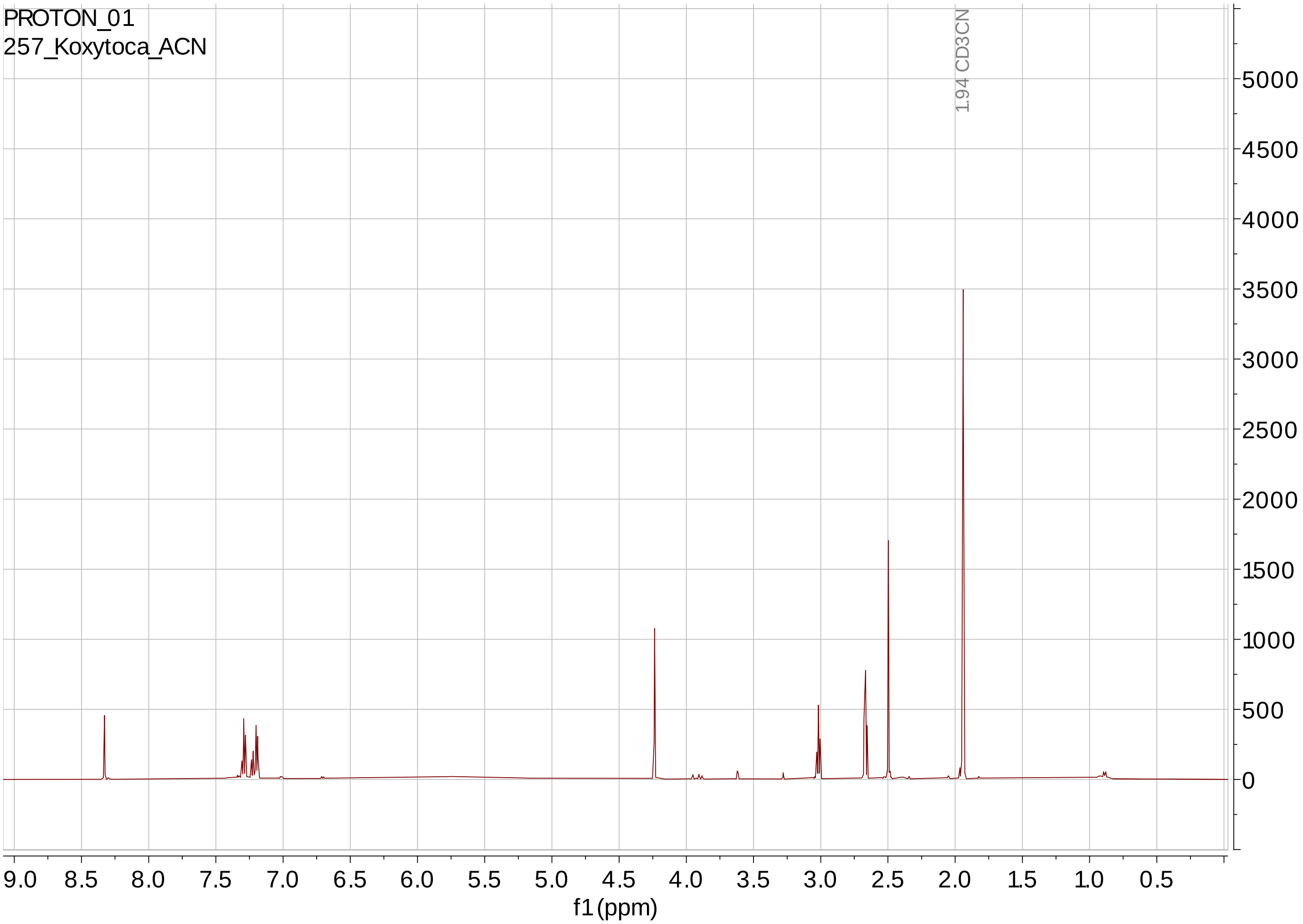

Figure S87. gCOSY spectrum of **7** (600 MHz, acetonitrile-d_3).

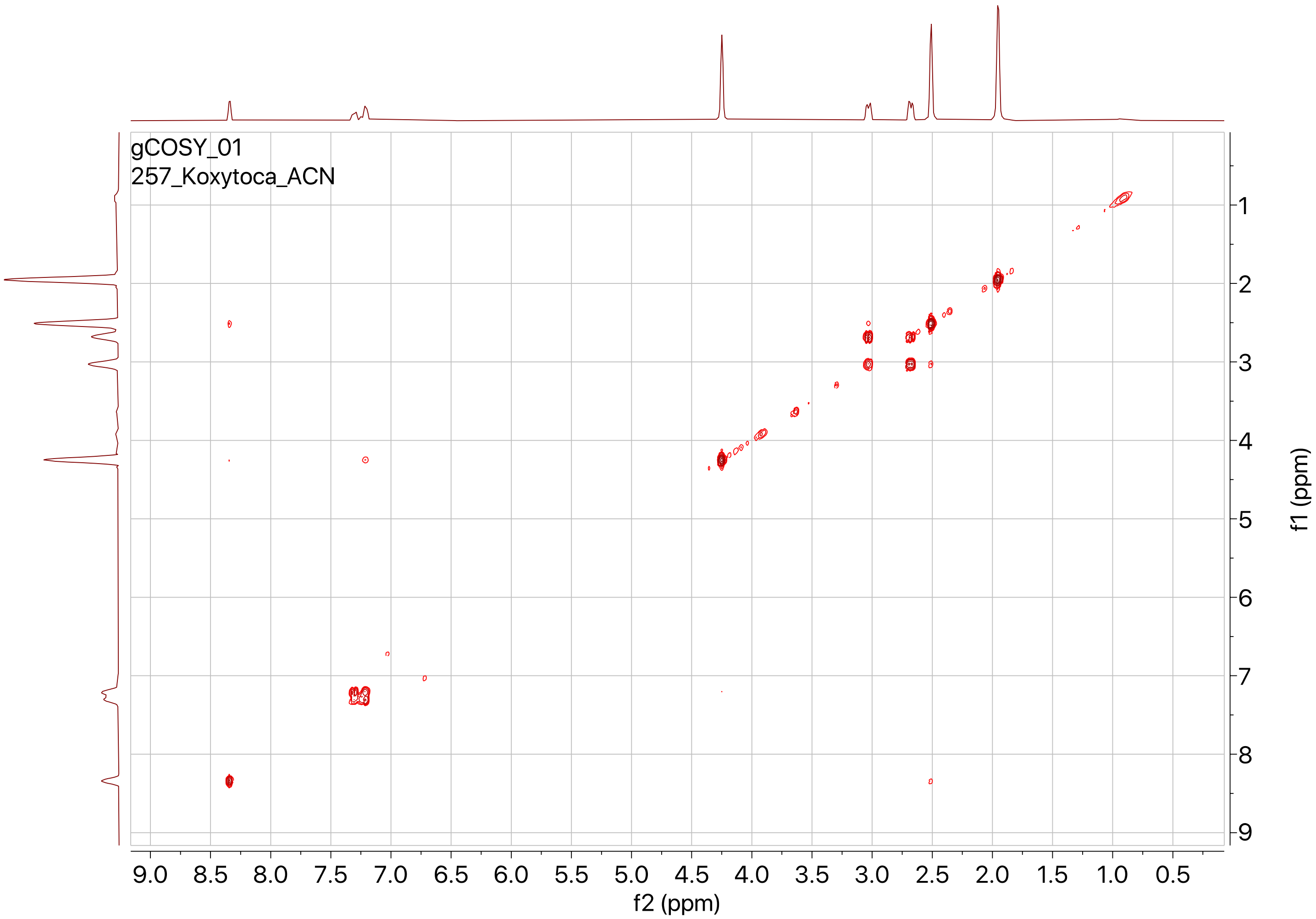

Figure S88. gHSQC spectrum of **7** (600 MHz, acetonitrile-d_3).

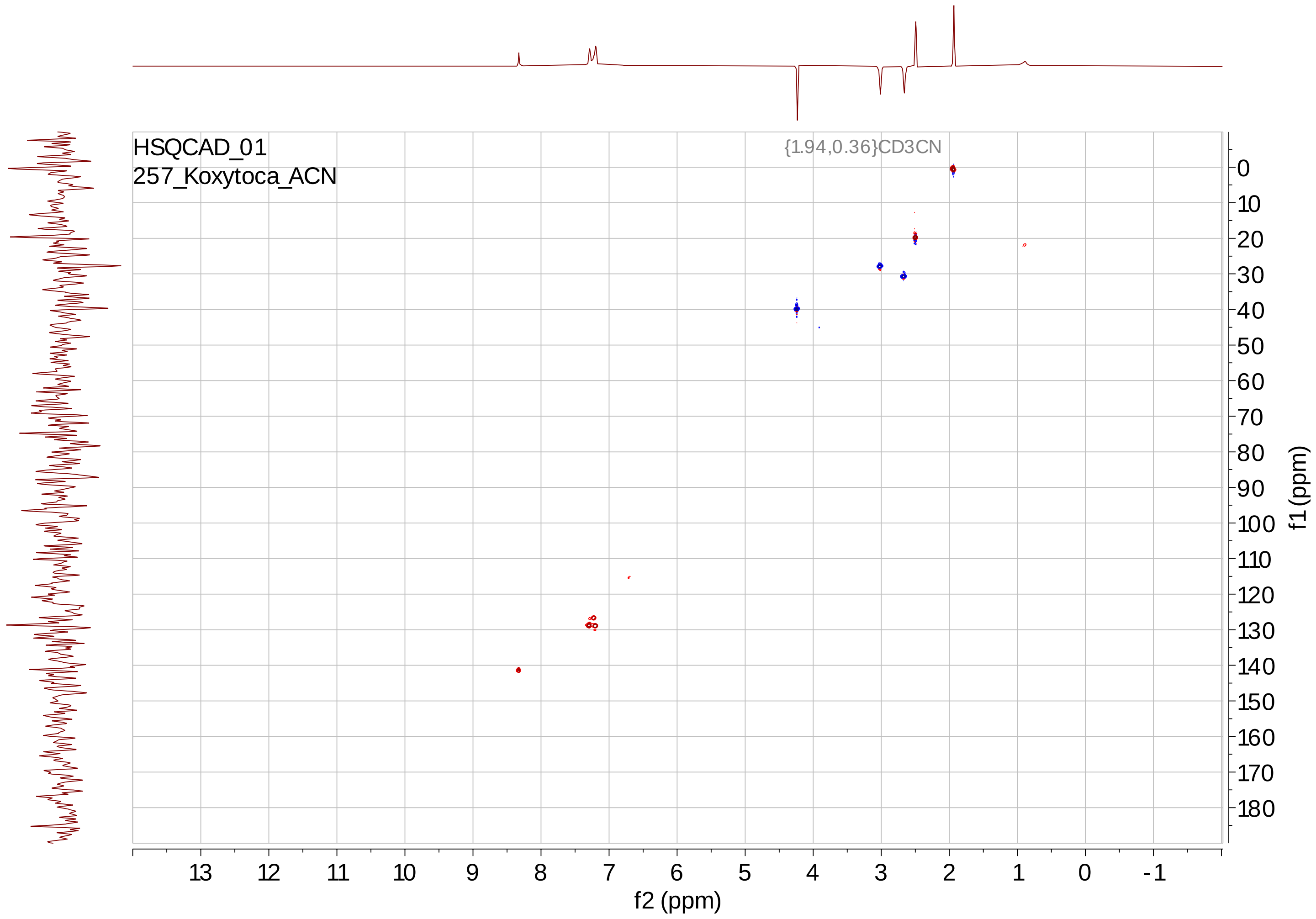

Figure S89. gHMBC spectrum of **7** (600 MHz, acetonitrile-d_3).

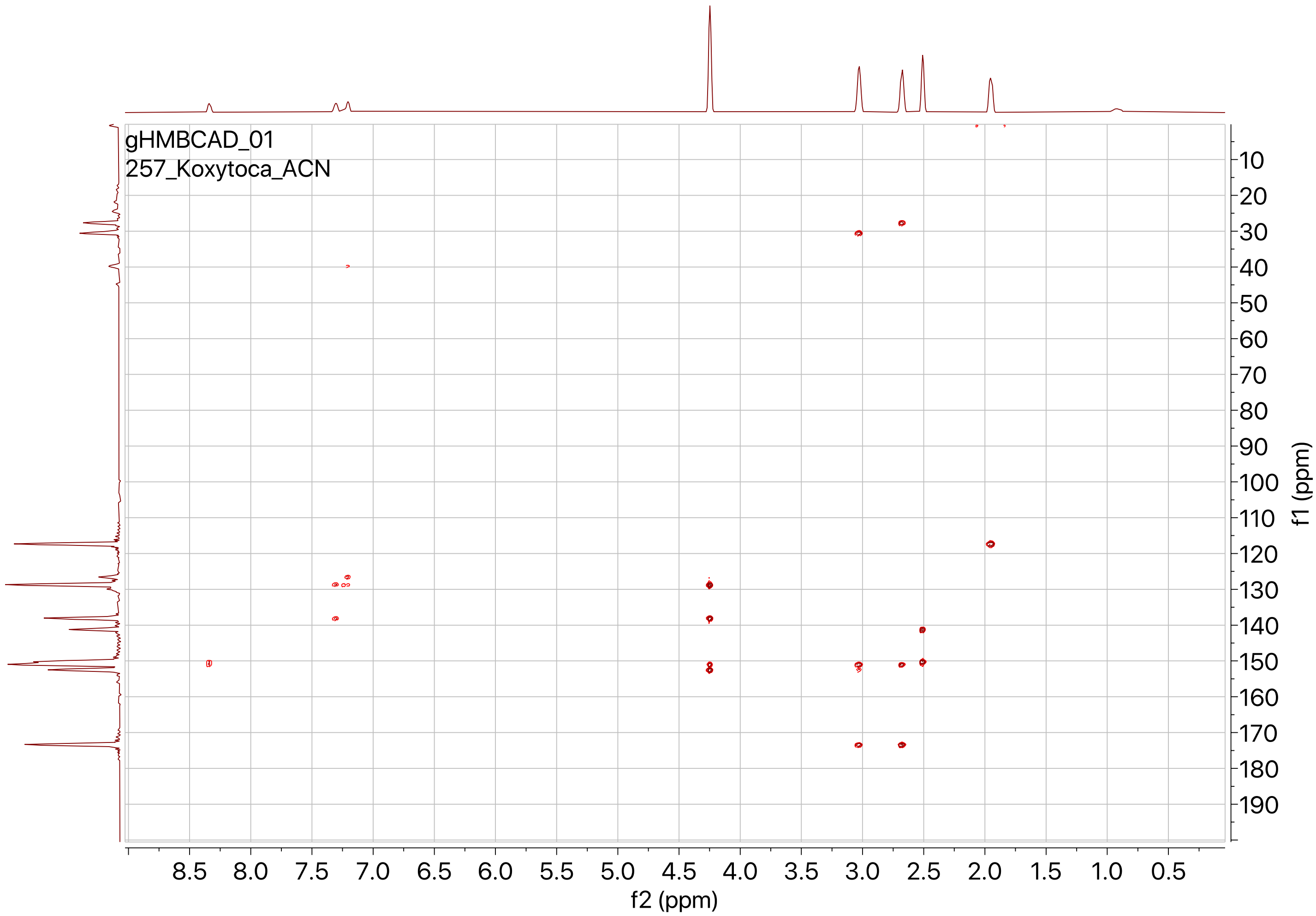

Figure S90. ^{1}H, ^{15}N-gHMBC spectrum of **7** (600 MHz, acetonitrile-d_3).

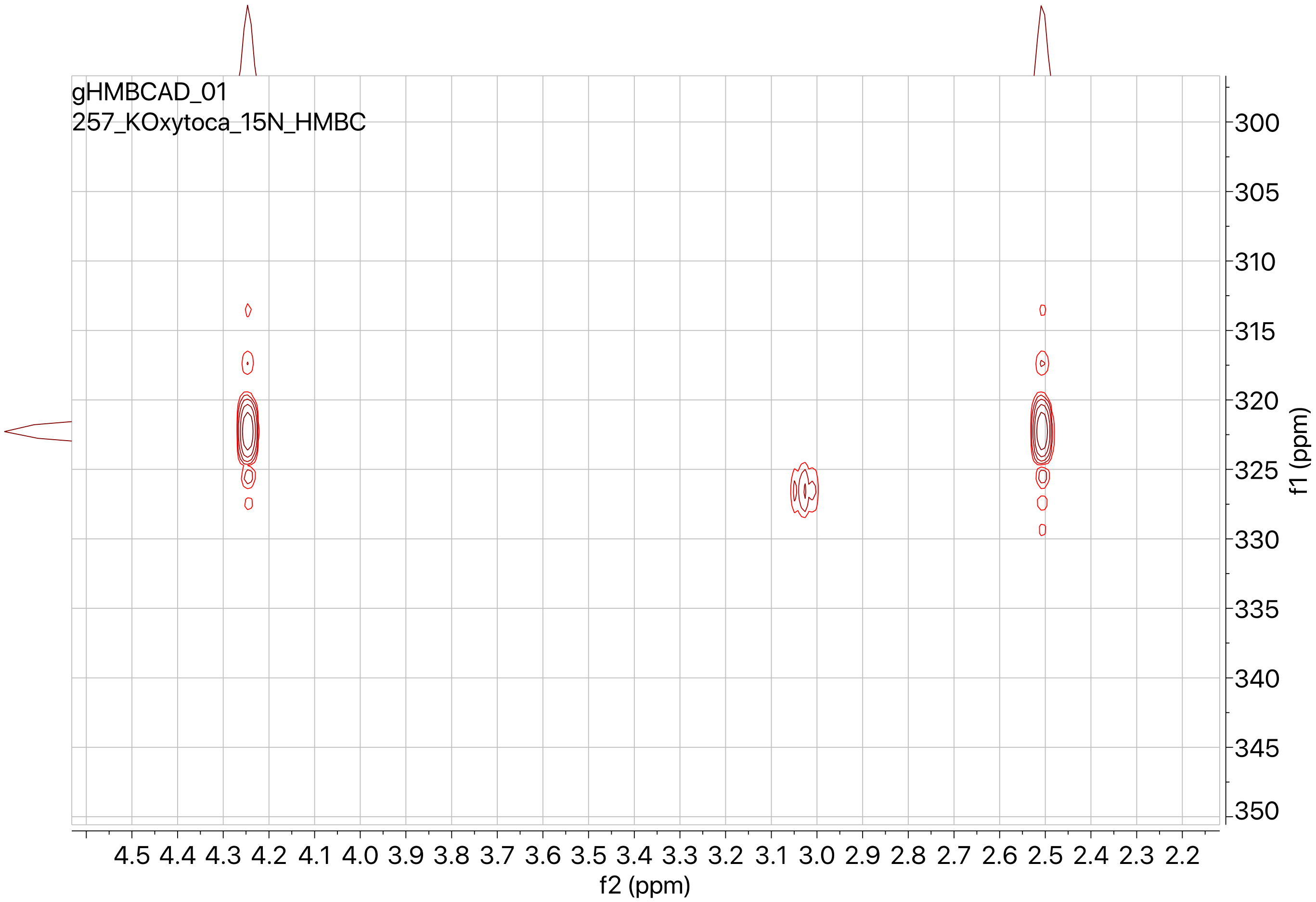

Figure S91. ^{1}H NMR spectrum of **9-Me** (600 MHz, acetonitrile-d_3).

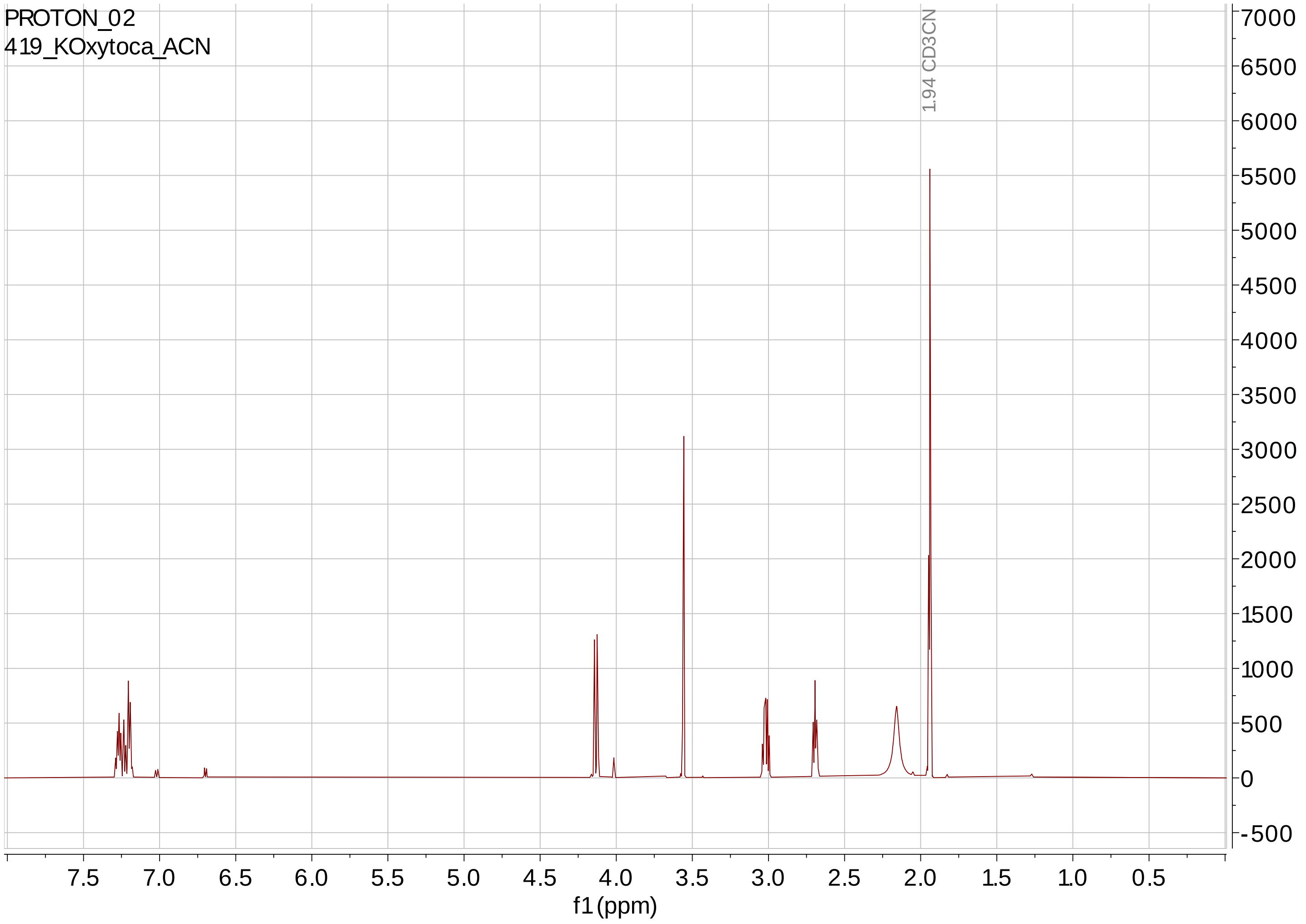

Figure S92. gCOSY spectrum of **9-Me** (600 MHz, acetonitrile-d_3).

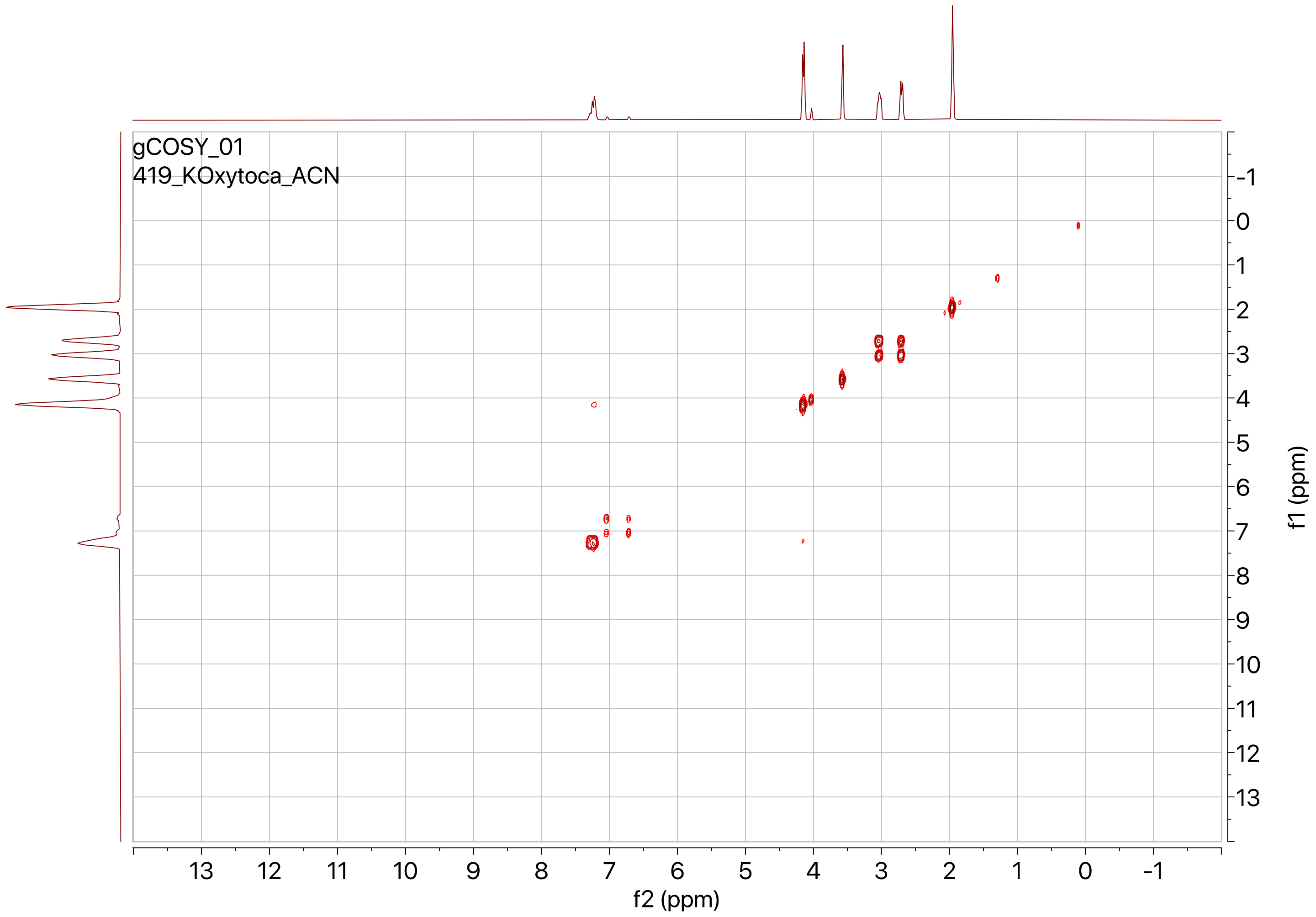

Figure S93. gHSQC spectrum of **9-Me** (600 MHz, acetonitrile-d_3).

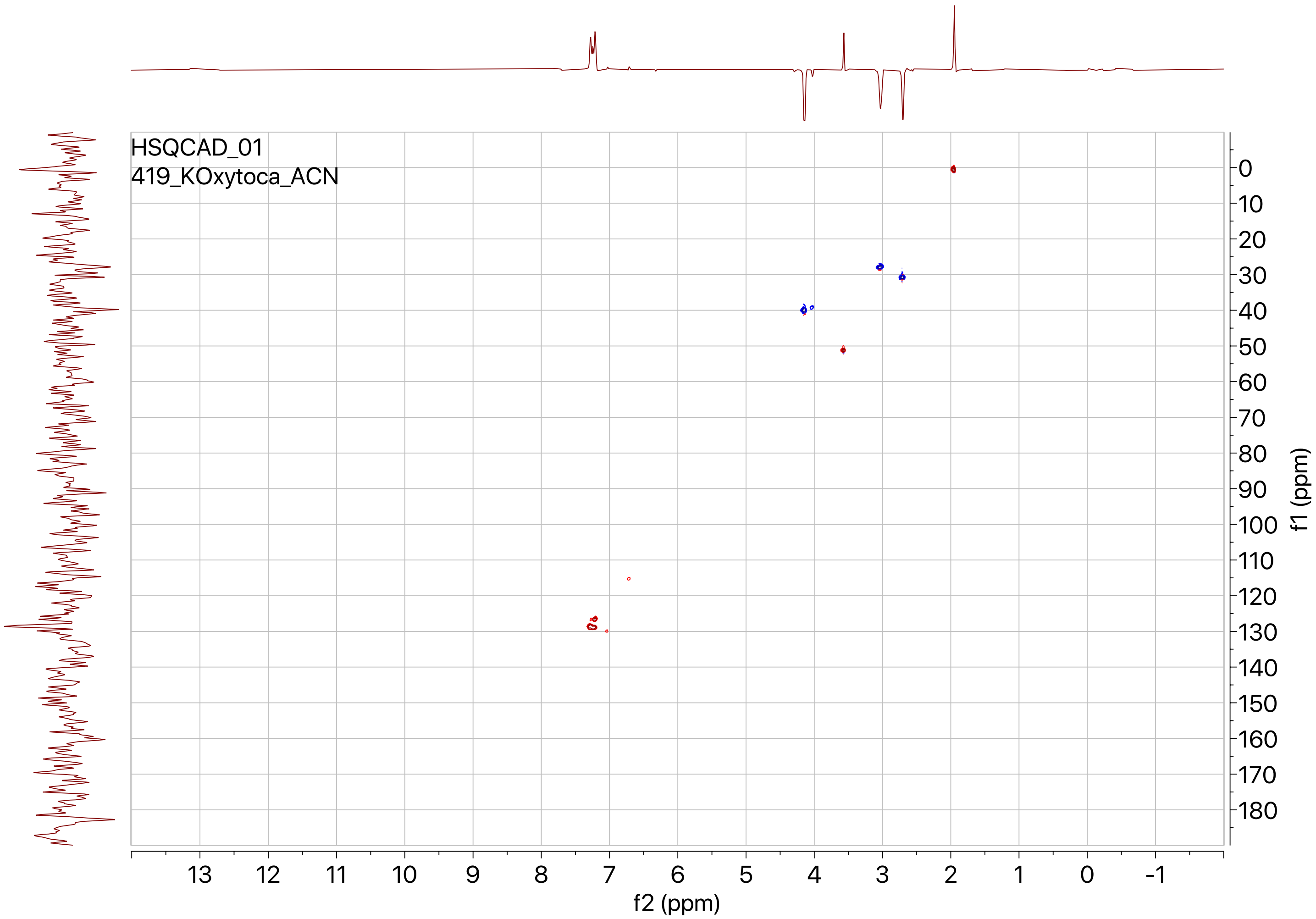

Figure S94. gHMBC spectrum of **9-Me** (600 MHz, acetonitrile-d_3).

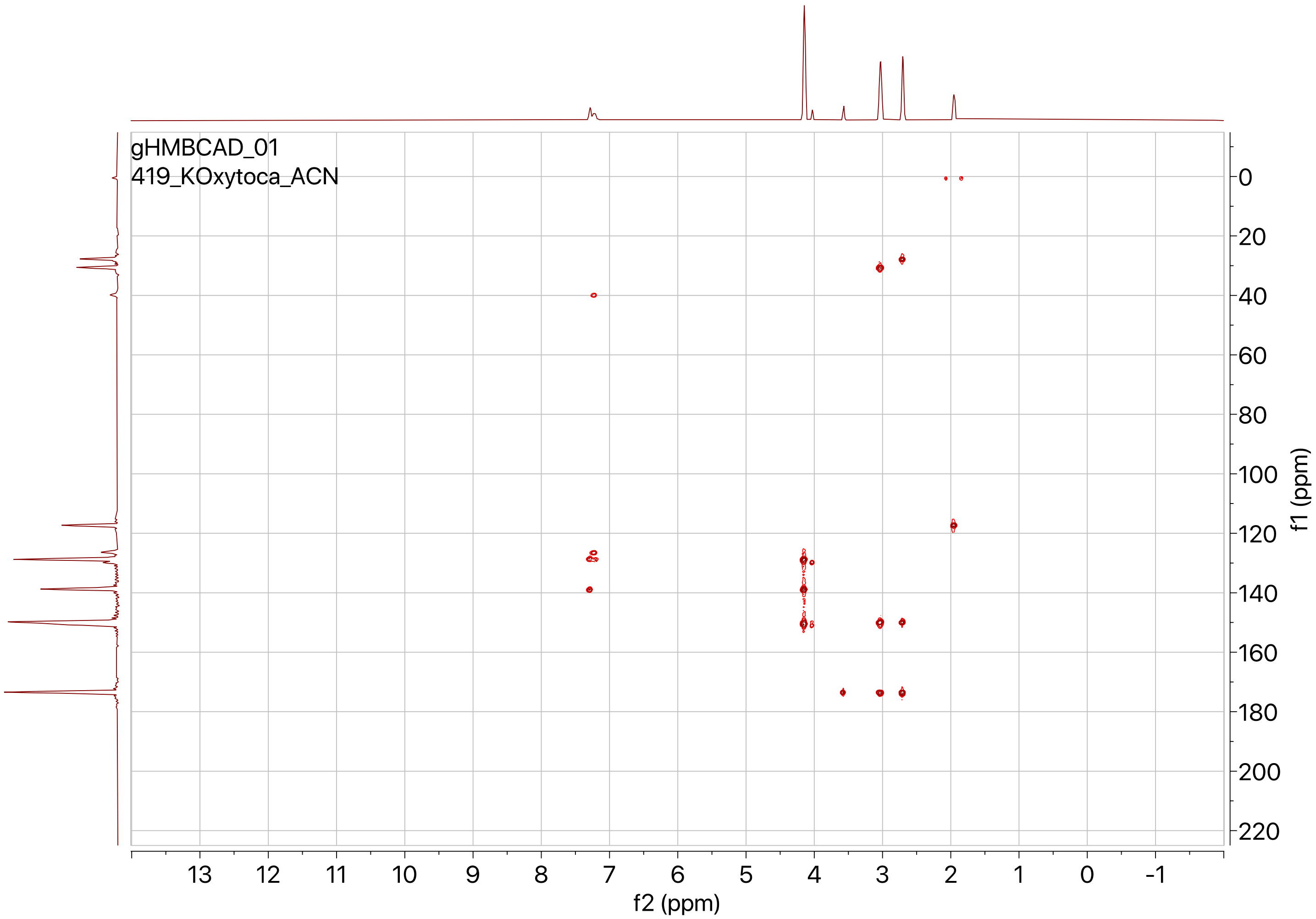

Figure S95. ^{1}H, ^{15}N-gHMBC spectrum of **9-Me** (600 MHz, acetonitrile-*d*$_3$).

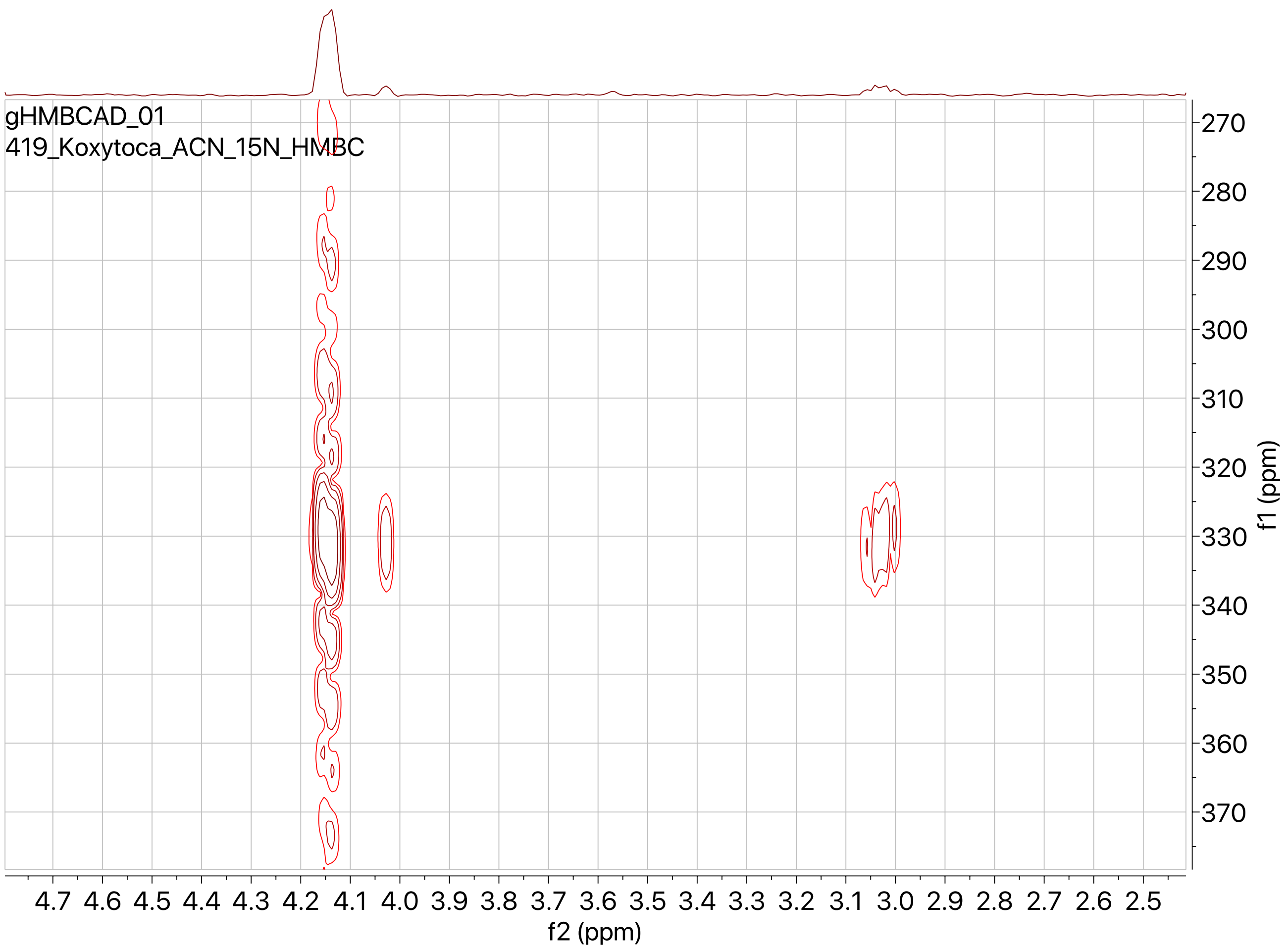

Figure S96. ^{1}H NMR spectrum of **18** (400 MHz, methanol-d_4).

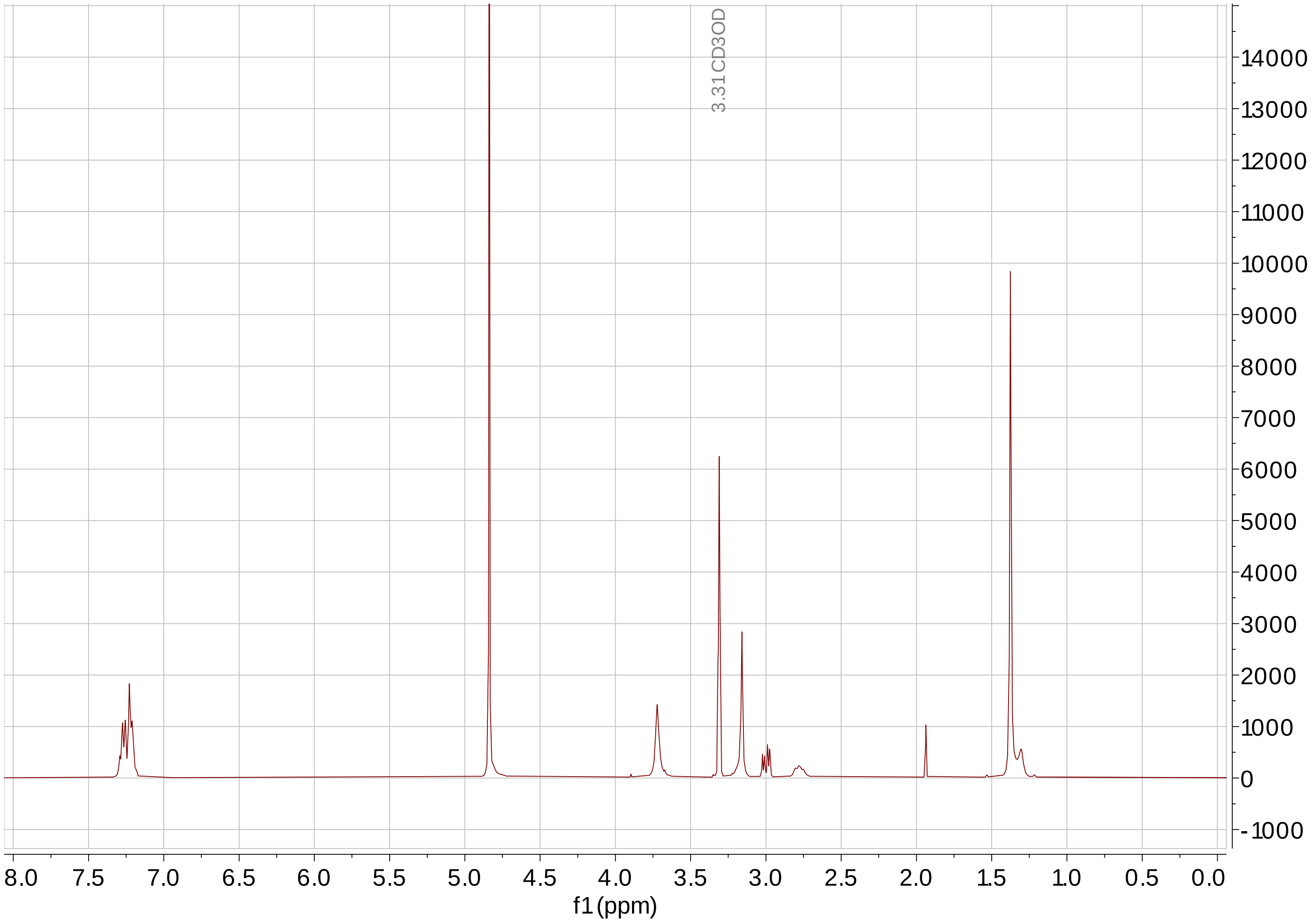

Figure S97. ^{13}C NMR spectrum of **18** (400 MHz, methanol-d_4).

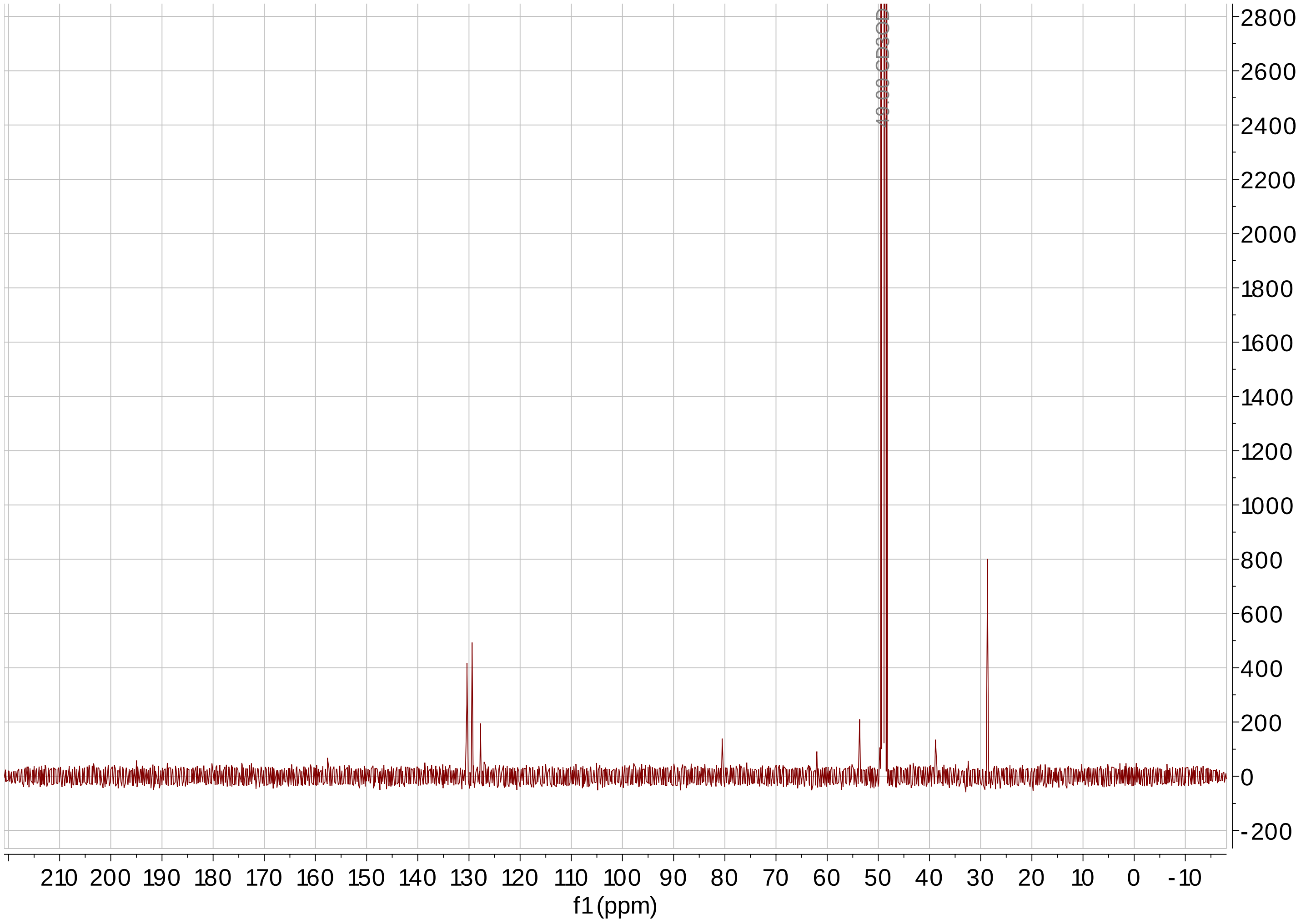

Figure S98. ^1H NMR spectrum of **19** (400 MHz, methanol-d_4).

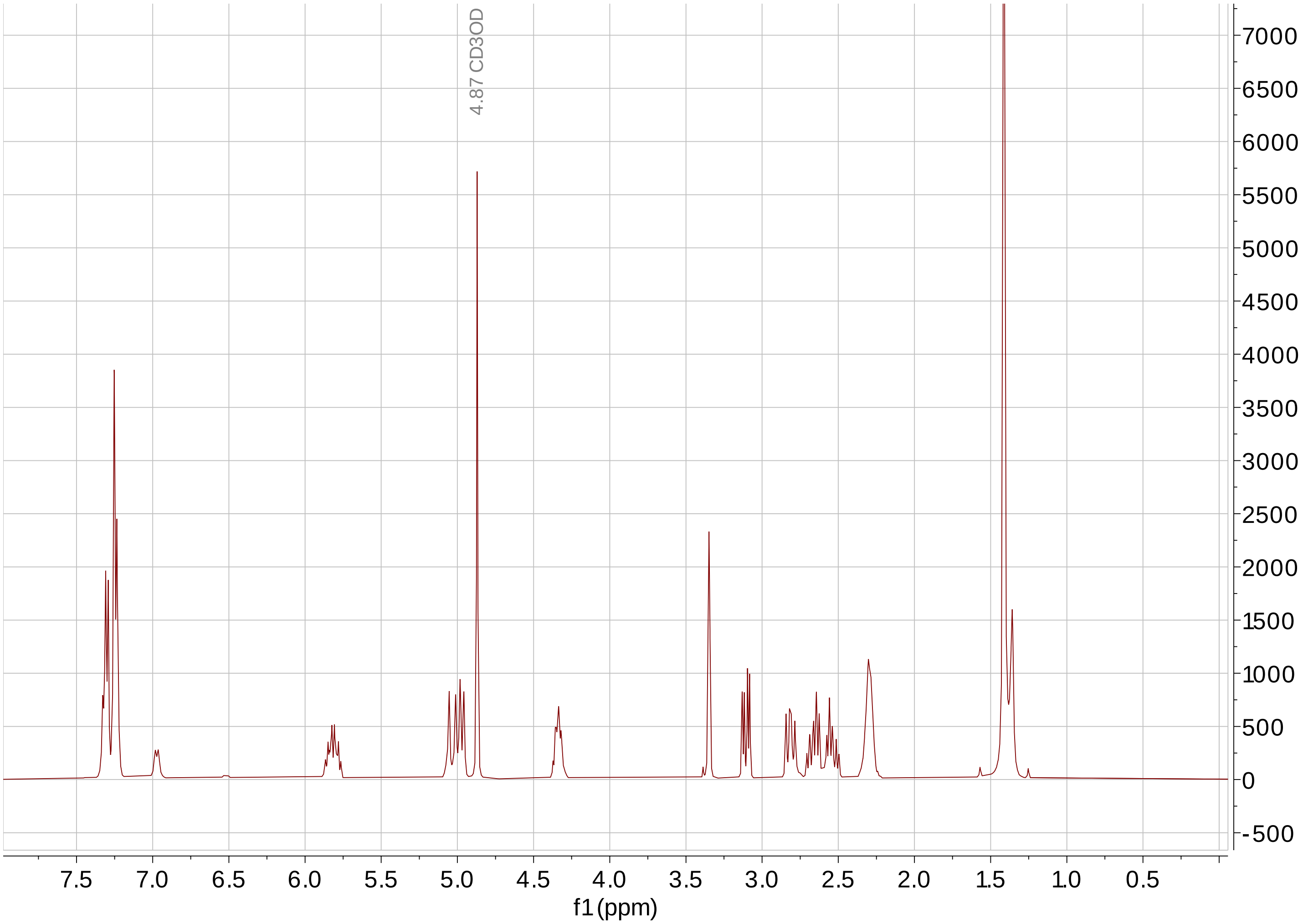

Figure S99. ^{13}C NMR spectrum of **19** (400 MHz, methanol-d_4).

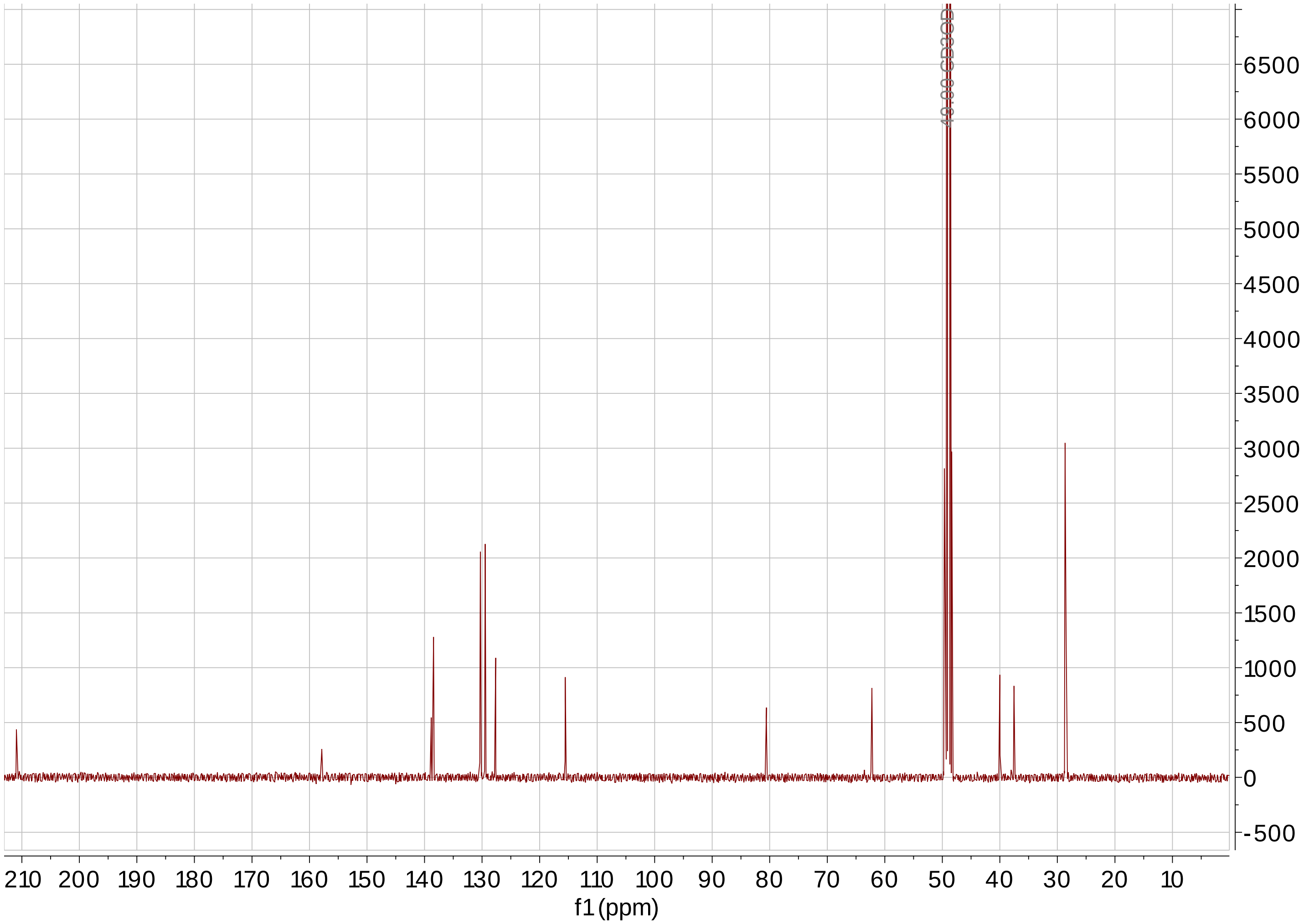

Figure S100. ^{1}H NMR spectrum of **20** (400 MHz, methanol-d_4).

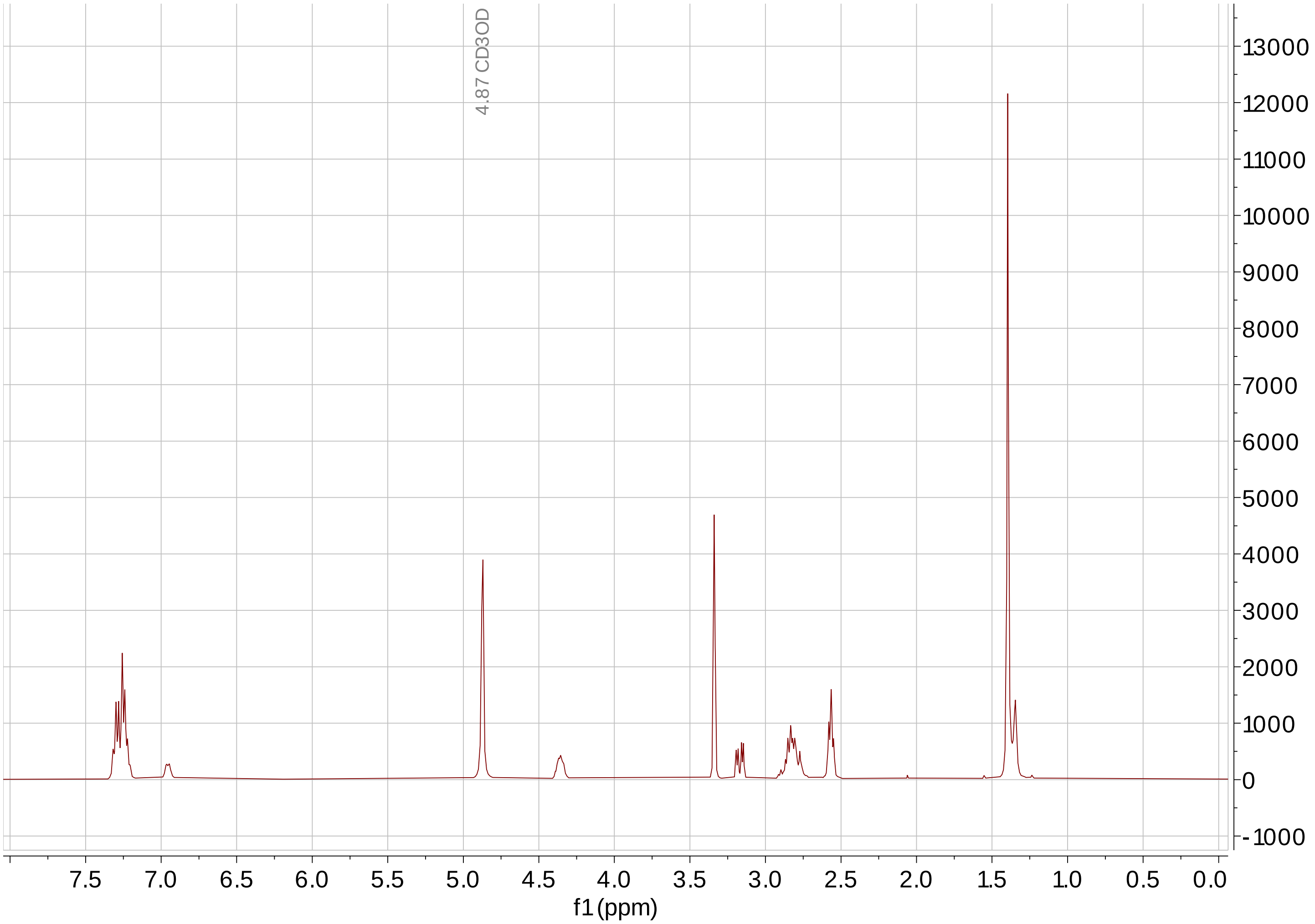

Figure S101. ^{13}C NMR spectrum of **20** (400 MHz, methanol-d_4).

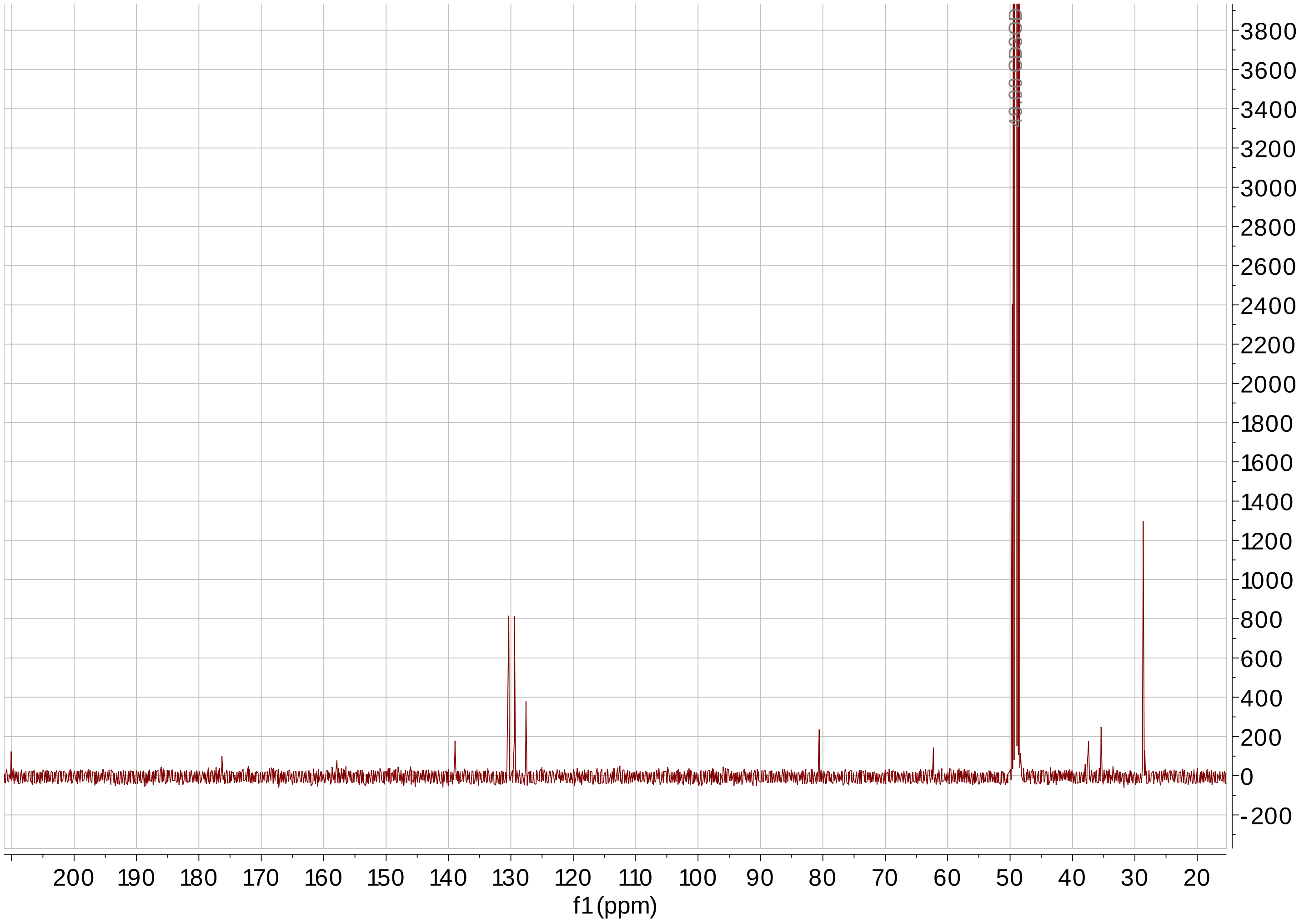

Figure S102. ^{1}H NMR spectrum of **12** (400 MHz, methanol-d_4).

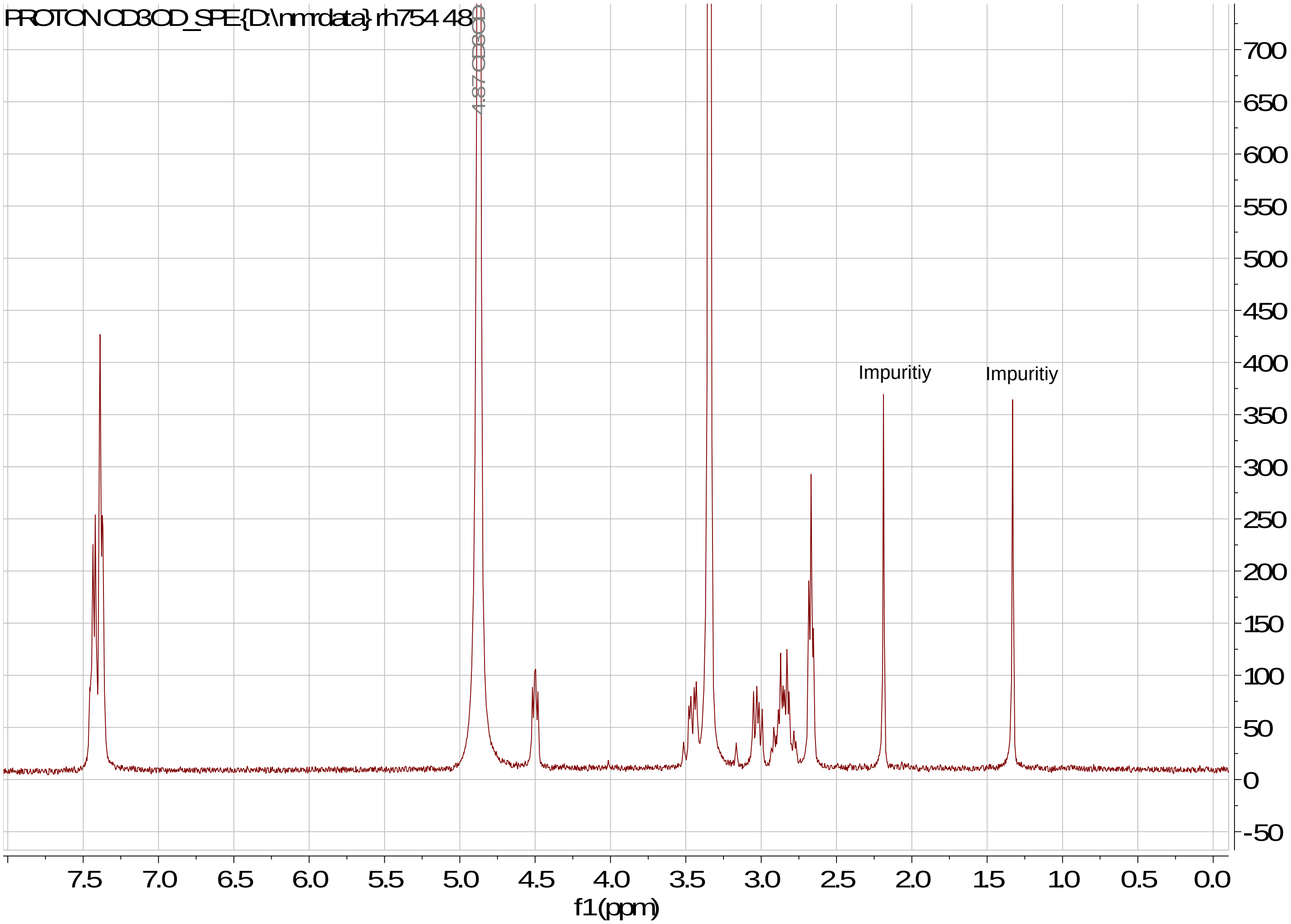

Figure S103. RNA-Seq volcano plot analysis of genes differentially regulated by pyrazine **6** relative to a DMSO vehicle control. Points marked in blue have a $-\log(P_{adj}) > 2$ and $abs(\log_2 FC) > 1$. Points marked in red are related to iron acquisition pathways. Table denotes red points.

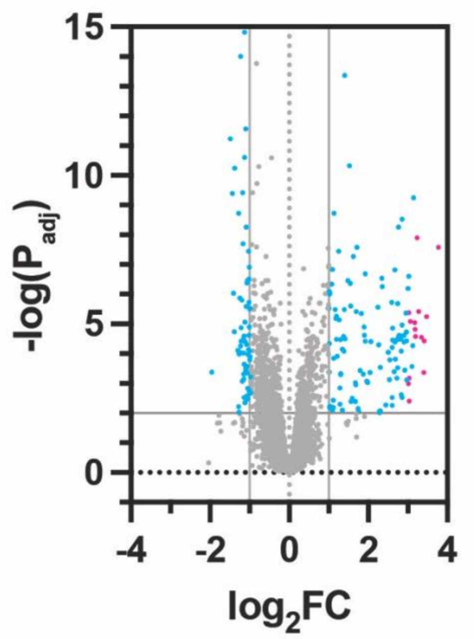

Gene	Log_2FC (**6**:DMSO)	$-\log(P_{adj})$
FepA	3.770261949	7.58191093
FecR	3.476640588	5.24147027
KOX_00800 (Iron-Related)	3.418506266	4.42855019
KOX_20750 (TonB-Dep. Recp.)	3.408493668	3.37257407
KOX_00795 (Iron-Related)	3.344593126	4.5546924
EfeB	3.270577057	5.41564714
EfeO	3.236816017	7.89575019
KOX_00790 (Iron-Related)	3.197170412	4.5864913
FecI	3.184433729	4.81653608
KOX_13900 (in *ent* Operon)	3.172959511	5.04823347
entE	3.065681297	5.08433709
KOX_13650 (Siderophore Recp.)	3.037639114	3.18060435
entF	3.024919848	5.38218324
KOX_13655 (enterochelin esterase-related enz.)	3.017669936	2.99200636

Figure S104. Relative abundances of enterobactin monomer, dimer, and linear trimer between wildtype and Δ*nanA* *K. oxytoca* strains when supplemented with Neu5Ac (n = 3). Statistical analyses performed through an unpaired two-tailed t-test; n.s. indicates a non-significant difference, * indicates p < 0.05; ** indicates p < 0.01, and *** indicates p < 0.001. Error bars indicate standard deviation.

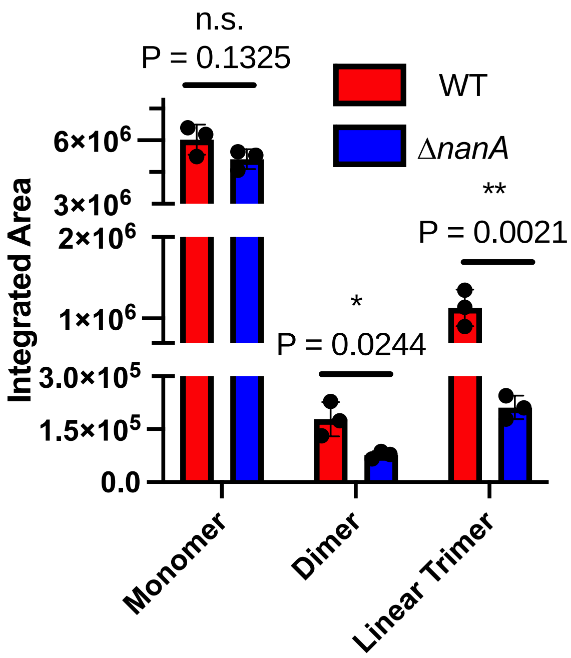

Supplement: Supplementary file 1 — Supplementary Information [file 41467_2024_53185_MOESM1_ESM.pdf]
