# Supplementary material for: Mucosal sugars delineate pyrazine vs pyrazinone autoinducer signaling in Klebsiella oxytoca
Source: Nat Commun. 2024 Oct 16;15:8902. doi: 10.1038/s41467-024-53185-6 (PMC11480411; doi:10.1038/s41467-024-53185-6)
Supplement: Supplementary file 2 — Reporting Summary [file 41467_2024_53185_MOESM2_ESM.pdf]

nature portfolio

Corresponding author(s): Jason M. Crawford

Last updated by author(s): Sep 23, 2024

Reporting Summary

Nature Portfolio wishes to improve the reproducibility of the work that we publish. This form provides structure for consistency and transparency in reporting. For further information on Nature Portfolio policies, see our Editorial Policies and the Editorial Policy Checklist.

Statistics

For all statistical analyses, confirm that the following items are present in the figure legend, table legend, main text, or Methods section.

n/a	Confirmed	
☐	☒	The exact sample size (*n*) for each experimental group/condition, given as a discrete number and unit of measurement
☐	☒	A statement on whether measurements were taken from distinct samples or whether the same sample was measured repeatedly
☐	☒	The statistical test(s) used AND whether they are one- or two-sided<br>*Only common tests should be described solely by name; describe more complex techniques in the Methods section.*
☒	☐	A description of all covariates tested
☒	☐	A description of any assumptions or corrections, such as tests of normality and adjustment for multiple comparisons
☐	☒	A full description of the statistical parameters including central tendency (e.g. means) or other basic estimates (e.g. regression coefficient) AND variation (e.g. standard deviation) or associated estimates of uncertainty (e.g. confidence intervals)
☐	☒	For null hypothesis testing, the test statistic (e.g. *F*, *t*, *r*) with confidence intervals, effect sizes, degrees of freedom and *P* value noted<br>*Give P values as exact values whenever suitable.*
☒	☐	For Bayesian analysis, information on the choice of priors and Markov chain Monte Carlo settings
☒	☐	For hierarchical and complex designs, identification of the appropriate level for tests and full reporting of outcomes
☒	☐	Estimates of effect sizes (e.g. Cohen's *d*, Pearson's *r*), indicating how they were calculated

Our web collection on statistics for biologists contains articles on many of the points above.

Software and code

Policy information about availability of computer code

Data collection	High-resolution electrospray ionization mass spectrometry (HRMS) was performed on an Agilent iFunnel 6550 quadrupole time-of-flight (QTOF) mass spectrometry instrument coupled to an Agilent 1290 Infinity HPLC system and a Kintex 5 µm C18 100 Å column (250 × 4.6 mm). Chromatography was performed with a linear water-acetonitrile gradient containing 0.1% formic acid at 0.7 mL/min over 30 minutes. The gradient proceeded from 5% acetonitrile to 50% acetonitrile, followed by either a 3- or 10-minute 100% acetonitrile wash. Mass spectra were recorded in positive ionization mode with a mass range of 100 m/z to 1700 m/z. Targeted MS2 analysis was performed with Iso width set to 1.3 m/z (narrow width) and a fixed collision energy of 20 CE. UV-vis spectra were obtained on an Agilent 1260 Infinity system equipped with a photo diode array (PDA) detector. NMR spectroscopy was recorded on either an Agilent 600 MHz NMR spectrometer (DD2) equipped with an inverse cold probe (3 mm), employing standard NMR pulse sequences, or a Bruker 400 MHz NMR spectrometer equipped with a broadband probe, also employing standard NMR pulse sequences.
Data analysis	For MS-based determination of leupeptin activation, wildtype K. oxytoca ATCC 8724 was cultured in 5 mL of M9 minimal medium supplemented with 5 g/L casamino acids and 0.4% Neu5Ac. A similar culture lacking Neu5Ac supplementation was also generated as a comparison. The cultures were incubated at 30 ̊C for 48 hours at 250 rpm and then dried at room temperature under reduced pressure (Genevac). The dried samples were resuspended in 200 µL of 1:1 methanol:water, and 3 µL of the samples were subjected to MS and MS2 profiling via HPLC-QTOF-MS (with PDA detector), as described above. The XCMS Online web-based platform54 was used to identify molecular features present in the Neu5Ac supplemented cultures. Furthermore, the GNPS online repository48 was used to link features with similar MS2 spectra in a molecular network. This chromatography and MS protocol was used for all MS experiments in this work. When sugars other than Neu5Ac were used to supplement cultures, they were present at 0.4%. To measure the area under the curves for specific molecular features, the exact monoprotonated [M+H]+ mass was calculated and extracted ion chromatographs (EICs) with a 10-ppm error were constructed. The peaks in the EICs were integrated with the Agilent Mass Hunter Qualitative Analysis software. UV-vis chromatographs were generated with a detection wavelength of 310 nm (bandwidth 4 nm).

Molecular networks were created using the online workflow (https://ccms-ucsd.github.io/GNPSDocumentation/) on the GNPS website (http://gnps.ucsd.edu). The data was filtered by removing all MS2 fragment ions within +/- 17 Da of the precursor m/z. MS2 spectra were window filtered by choosing only the top 6 fragment ions in the +/- 50 Da window throughout the spectrum. The precursor ion mass tolerance was set to .05 Da and a MS2 fragment ion tolerance of .1 Da. A network was then created where edges were filtered to have a cosine score above 0.7 and more than 2 matched peaks. Further, edges between two nodes were kept in the network if and only if each of the nodes appeared in each other's respective top 10 most similar nodes. Finally, the maximum size of a molecular family was set to 100, and the lowest scoring edges were removed from molecular families until the molecular family size was below this threshold. The spectra in the network were then searched against GNPS' spectral libraries. The library spectra were filtered in the same manner as the input data. All matches kept between network spectra and library spectra were required to have a score above 0.7 and at least 2 matched peaks. Nodes were annotated with the high-resolution masses of the molecular features fragmented in the metabolomic profiling experiment.
RNA-seq analysis was conducted by the Yale Center for Genome Analysis. Sequences were aligned to the K. oxytoca ATCC 8724 genome (NC_016612.1). Differential expression analysis was performed through DESeq2 and pathway enrichment analysis was performed through the Funage-Pro web-based platform.
The amino acid sequence of the K. oxytoca Pyr protein was used to BLAST "All genomes" on the DOE Integrated Microbial Genomes & Microbes (MG) database with an E-value cutoff of 1e-5. The resulting list of 459 homologs from this database encompassed 26 species of bacteria. Representative strains of the 26 species were selected and their operon architectures were plotted on a phylogenetic tree constructed with phyloT.
The sequence similarity network was generated via the Enzyme Similarity Tool67 using the amino acid sequence of the K. oxytoca Pyr protein with an E-value cutoff of 1e-50 and an alignment score of 100. The network was visualized with Cytoscape and nodes with >90% sequence homology were clustered.
For all statistical analysis and curve fitting, the software GraphPad Prism was used. For determining statistical significance, two-tailed unpaired t-tests were performed. * indicates $p < 0.05$; ** indicates $p < 0.01$; *** indicates $p < 0.001$.

For manuscripts utilizing custom algorithms or software that are central to the research but not yet described in published literature, software must be made available to editors and reviewers. We strongly encourage code deposition in a community repository (e.g. GitHub). See the Nature Portfolio guidelines for submitting code & software for further information.

Data

Policy information about availability of data

All manuscripts must include a data availability statement. This statement should provide the following information, where applicable:

- Accession codes, unique identifiers, or web links for publicly available datasets
- A description of any restrictions on data availability
- For clinical datasets or third party data, please ensure that the statement adheres to our policy

All data supporting the findings of this study are available within the manuscript and its Supplementary Information. The source data generated in this study have been deposited in the Figshare database under accession code https://doi.org/10.6084/m9.figshare.25301908. The RNA-seq data generated in this study have been deposited in the NCBI database under accession code PRJNA1124412. The metabolomics data generated in this study have been deposited in the MassIVE database under accession code MSV000095925.

Research involving human participants, their data, or biological material

Policy information about studies with human participants or human data. See also policy information about sex, gender (identity/presentation), and sexual orientation and race, ethnicity and racism.

Reporting on sex and gender	N/A
Reporting on race, ethnicity, or other socially relevant groupings	N/A
Population characteristics	N/A
Recruitment	N/A
Ethics oversight	N/A

Note that full information on the approval of the study protocol must also be provided in the manuscript.

Field-specific reporting

Please select the one below that is the best fit for your research. If you are not sure, read the appropriate sections before making your selection.

☒ Life sciences ☐ Behavioural & social sciences ☐ Ecological, evolutionary & environmental sciences

For a reference copy of the document with all sections, see nature.com/documents/nr-reporting-summary-flat.pdf

Life sciences study design

All studies must disclose on these points even when the disclosure is negative.

Sample size	All experiments were performed in triplicates except for the experiments shown in Fig 2e and Supplementary Data Figure 2b which were performed in duplicates.
Data exclusions	No data were excluded from any analysis.
Replication	All experimental were repeated at least twice and could be replicated. The findings from our RNA-Seq analysis was validated through mass spectrometry at least twice.
Randomization	Randomization was not relevant in this study.
Blinding	Blinding was not relevant in this study.

Reporting for specific materials, systems and methods

We require information from authors about some types of materials, experimental systems and methods used in many studies. Here, indicate whether each material, system or method listed is relevant to your study. If you are not sure if a list item applies to your research, read the appropriate section before selecting a response.

Materials & experimental systems

n/a	Involved in the study	
☒	☐	Antibodies
☐	☒	Eukaryotic cell lines
☒	☐	Palaeontology and archaeology
☒	☐	Animals and other organisms
☒	☐	Clinical data
☒	☐	Dual use research of concern
☒	☐	Plants

Methods

n/a	Involved in the study	
☒	☐	ChIP-seq
☒	☐	Flow cytometry
☒	☐	MRI-based neuroimaging

Eukaryotic cell lines

Policy information about cell lines and Sex and Gender in Research

Cell line source(s)	HTLA cell is a HEK293T cell derived cell line that stably express b-arrestin-TEV and tTA-Luciferase, which is a kind gift from Gilad Barnea (Brown University)
Authentication	HTLA cell line is maintained in medium supplemented with hygromycin B and puromycin to select for the essential construct for GPCR activity report. And the response of the cell line is confirmed by positive controls.
Mycoplasma contamination	Cell line was negative for mycoplasma.
Commonly misidentified lines (See ICLAC register)	N/A

Plants

Seed stocks	N/A
Novel plant genotypes	N/A
Authentication	N/A
